# Rhodamine–Hoechst positional isomers for highly efficient staining of heterochromatin†
†Electronic supplementary information (ESI) available. See DOI: 10.1039/c8sc05082a


**DOI:** 10.1039/c8sc05082a

**Published:** 2018-12-12

**Authors:** Jonas Bucevičius, Jan Keller-Findeisen, Tanja Gilat, Stefan W. Hell, Gražvydas Lukinavičius

**Affiliations:** a Department of NanoBiophotonics , Max Planck Institute for Biophysical Chemistry , Am Fassberg 11 , Göttingen , 37077 , Germany . Email: grazvydas.lukinavicius@mpibpc.mpg.de

## Abstract

Hoechst conjugates to fluorescent dyes are popular DNA stains for live-cell imaging, but the relationship between their structure and performance remains elusive. This study of carboxyrhodamine–Hoechst 33258 conjugates reveals that a minimal change in the attachment point of the dye has dramatic effects on the properties of the final probe. All tested 6′-carboxyl dye-containing probes exhibited dual-mode binding to DNA and formed a dimmer complex at high DNA concentrations. The 5′-carboxyl dye-containing probes exhibited single-mode binding to DNA which translated into increased brightness and lower cytotoxicity. Up to 10-fold brighter nuclear staining by the newly developed probes allowed acquisition of stimulated emission depletion (STED) nanoscopy images of outstanding quality in living and fixed cells. Therefore we were able to estimate a diameter of ∼155 nm of the heterochromatin exclusion zones in the nuclear pore region in living cells and intact chicken erythrocytes and to localize telomeres relative to heterochromatin in living U-2 OS cells. Employing the highly efficient probes for two-color STED allowed visualization of DNA and tubulin structures in intact nucleated erythrocytes – a system where imaging is greatly hampered by high haemoglobin absorbance.

## Abstract 2

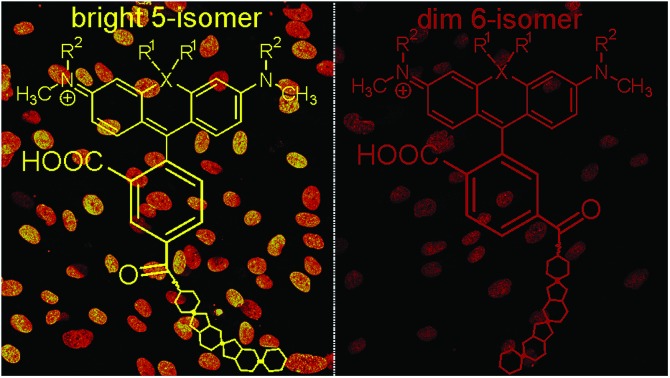
Nuclei of living cells fluoresce brighter after staining with rhodamine–Hoechst 5-isomers.

## Introduction

Chromatin is a complex structure composed of proteins, RNA and DNA. Cell biology studies often involve fluorescence microscopy of chromatin in living cells, allowing observation of cell division, apoptosis, necrosis and other important events.^1^ Many commercialized assays use DNA-specific blue fluorescent dyes DAPI and Hoechst. These stains remain the first choice for many applications, due to simplicity in usage, low cost and no requirement for genetic modification.^2^ Living cells are generally stained with Hoechst 33342 since it offers greater cell permeability and lower cytotoxicity.^3^ Recently, the bisbenzimide core of Hoechst 33342 was exploited as a DNA-targeting ligand for other fluorescent dyes.^4^ One of the newest cell-permeable DNA probes SiR-Hoechst (commercial name “SiR-DNA”) is composed of Hoechst attached to 6′-carboxy silicon-rhodamine (SiR),^5^ which allows imaging of the cell nucleus in the far-red region of the spectrum with high resolution and low cytotoxicity.^6^ However, staining of a number of cell lines revealed its weak points: efflux from cells by multidrug resistance pumps and relatively low brightness compared to the original Hoechst 33342.^7^ Further improvement of the properties is hindered by the poor understanding of how the structure of Hoechst-dye conjugates is related to their performance. To address this, we carried out an in-depth mechanistic study on Hoechst conjugates to xanthene type dyes: tetramethylrhodamine (TMR), two carbopyronines (580CP and 610CP), silicon-rhodamine (SiR) and germano-rhodamine (GeR).^5^,^8^ Systematic characterization of their photophysical properties, affinity to DNA, staining performance and cytotoxicity provided fundamentally new insights into the relationship between the structure and performance of Hoechst-derived DNA probes. All mentioned fluorophores can exist as 5′- or 6′-carboxy isomers, depending on the position of the second carboxylic group on the benzene ring, which is used for the formation of a covalent bond between the fluorophore and the recognition unit.^9^ Taking into account their almost identical photophysical properties, the importance of which regioisomer is used for the labelling is usually overlooked and the choice of the exact regioisomer is rarely explained. To date, there have been only a few reports where the performance of both regioisomers is compared and essential differences revealed.^10^ The final conjugates of such regioisomers can differ in molecular geometry and, most importantly, rotational/conformational freedom. This may significantly influence the binding or reactivity of the probe and manifest in different efficiencies of labelling, toxicity or quality of images. Herein, we demonstrate the importance of the choice of the regioisomer used and provide mechanistic explanations of the properties of Hoechst-based DNA probes. Our systematic *in vitro* and *in cellulo* studies revealed considerably different complexation of 5′- and 6′-regioisomer derived probes with the target DNA. The probes derived from 5′-carboxy fluorophores showed a single site binding mode, an up to 10× higher fluorescence signal in the cell nucleus and less cytotoxicity compared to 6′-regioisomers. This knowledge allowed us to obtain unprecedented quality of super-resolution images of nuclear DNA and measure heterochromatin exclusion zone (HEZ) diameter in living cells or intact chicken erythrocytes. Taking into consideration these and previously published results, we were encouraged to explore different regioisomers during the optimisation stage of construction of carboxyrhodamine based fluorescent probes.

## Experimental methods

### Estimation of absorbance and fluorescence increase upon SDS or hpDNA addition

Fluorescence increase of the probes upon SDS or hpDNA addition was measured by preparing 2 μM probe solution in PBS buffer (Lonza, Cat. No. BE17-516F) with or without 0.1% SDS (Acros Organics). The samples prepared in 3 ml glass bottles with caps were incubated at room temperature for 2 h before measurements.

The fluorescence increase of Hoechst-based probes binding to hpDNA was estimated using the following procedure: the probe from 1 mM DMSO stock solution was diluted to the final concentration of 2 μM in PBS buffer containing 30 μM of hpDNA (5′-CGCGAATTCGCGTTTTCGCGAATTCGCG-3′).

Absorption and fluorescence were measured on a multiwell plate reader Spark® 20M (Tecan) in glass bottom 96-well plates (MatTek, Cat. No. PBK96G-1.5-5-F) at room temperature (25 °C). Absorption of solutions was recorded from 320 nm to 850 nm with a wavelength step size of 1 nm. The background absorption of the glass bottom plate was measured in wells containing only buffer and subtracted from the spectra of the samples. The fluorescence emission of the free dyes or final probes was recorded from 520 nm to 850 nm (for TMR, 495 nm exc., bandwidth 15 nm), 560 nm to 850 nm (for 580CP, 530 nm exc., bandwidth 15 nm), 600 nm to 850 nm (for 610CP, 570 nm exc., bandwidth 15 nm), and 620 nm to 850 nm (for SiR/GeR, 595 nm exc., bandwidth 15 nm) with a 5 nm emission bandwidth and 2 nm step size.

All samples were prepared in technical triplicates, which was repeated three times as three independent experiments performed on different days.

### Determination of quantum yields and lifetimes

The fluorescence quantum yields (absolute values) were obtained with a Quantaurus-QY absolute PL quantum yield spectrometer (model C11347-12, Hamamatsu) according to the manufacturer's instructions. Fluorescence lifetimes were measured with a Quantaurus-Tau fluorescence lifetime spectrometer (model C11367-32, Hamamatsu) according to the manufacturer's instructions. All measurements were performed in air-saturated PBS buffer containing 2 μM probe and 30 μM hpDNA after incubation for 2 h at room temperature.

### Determination of *K*_d_

*K*_d_ measurements were performed by titrating Hoechst or its derivatives in PBS (Lonza) with increasing concentrations of the 28 bp hpDNA in a 96-well plate and measuring the increase in fluorescence on a plate reader after 1 h incubation at room temperature. Hoechst 33342, TMR, 580CP, 610CP, GeR and SiR dyes were excited at 360 nm, 540 nm, 570 nm, 570 nm, 640 nm and 640 nm while recording emission at 480 nm, 580 nm, 610 nm, 640 nm, 670 nm and 670 nm respectively. The excitation and emission bandwidths were 10 nm for SiR/GeR and 15 nm for all other dyes. The *K*_d_ values were determined by plotting the emission signal *vs.* hpDNA concentration and fitting the curve in GraphPad Prism 6 to the “Two site-binding + offset” function:1
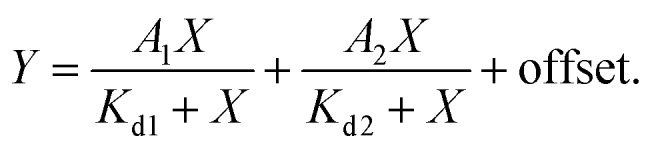
where *A*_1_ and *A*_2_ – fluorescence change after probe binding to the 1st and 2nd site respectively, *X* – target hpDNA concentration, *K*_d1_ and *K*_d2_ – dissociation constant of the probe from the 1st and 2nd site respectively, and offset – background fluorescence signal or “Single site binding” function:2
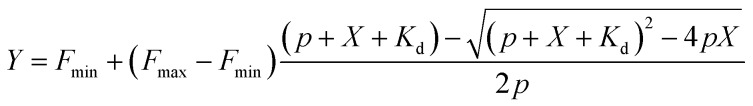
where *F*_min_ – fluorescence of the probe without the target, *F*_max_ – fluorescence of the probe at the saturating concentration, *p* – probe concentration, *X* – target hpDNA concentration, and *K*_d_ – dissociation constant of the probe. All measurements performed 3 times on different days, each time technical triplicates were measured.

### STED microscope with a 775 nm depletion laser

All confocal and STED images were acquired on an Abberior STED 775 QUAD scanning microscope (Abberior Instruments GmbH, Germany) equipped with 561 nm and 640 nm 40 MHz pulsed excitation lasers, a pulsed 775 nm 40 MHz STED laser, and an UPlanSApo 100×/1.40 Oil objective. The following detection windows were used: TMR/Cy3 channel 615/20 nm and Cy5 channel 685/70 nm. The voxel size was 15–30 nm in the *xy* plane and 150 nm in the *z*-axis for STED images acquired using this setup. The pixel size was 50–150 nm in the *xy* plane for confocal images acquired using this setup. Laser powers were optimized for each sample.

### Molecular docking

The DNA-Hoechst 33258 complex structure was downloaded from the PDB database (PDB ID: 8BNA).^11^ Only DNA molecules were used for the docking experiments. Ligands were drawn using ChemDraw Professional 15.1 and prepared for docking with AutoDock Tools version 1.5.6 (ref. 12). The docking simulation was performed using Vina Autodock version 1.1.2 (ref. 13). Twenty binding modes were generated starting from random configurations of ligands that had fully flexible torsional degrees of freedom.

### Image analysis

The methods used for the image analysis are described in the ESI.†

## Results and discussion

### Chemical synthesis of Hoechst derivatives

**Scheme 1:**
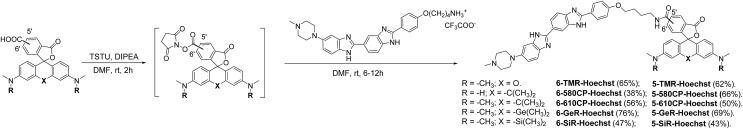
Synthesis of positional isomers of heteroatom substituted rhodamine–Hoechst conjugates.

**Fig. 1:**
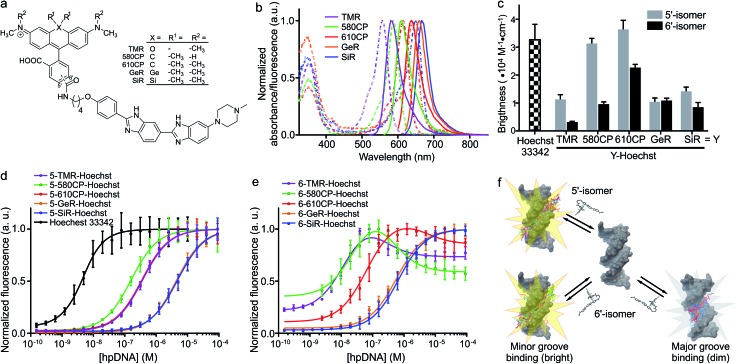
Spectral and binding characteristics of Hoechst-based probes. (a) Structures of the Hoechst derivatives analyzed in this work. (b) Absorption (dashed line) and emission (solid line) spectra of dyes used for the generation of the DNA probes. (c) Brightness of Hoechst 33342 and its derivatives after binding to specific DNA. Measurements performed in PBS containing 2 μM probe and 30 μM hairpin DNA (hpDNA). (d) Titration of 4 nM Hoechst 33342 and 10 nM Hoechst 5′-regioisomer conjugates with hpDNA. The data points are fitted to a single site binding equation. (e) Titration of 10 nM Hoechst 6′-regioisomer conjugates with hpDNA. **5-GeR-Hoechst** and **6-SiR-Hoechst** data points are fitted to a single site binding equation. In contrast, **6-TMR-Hoechst**, **6-580CP-Hoechst** and **6-610CP-Hoechst** data points can be fitted to the two site-binding equation. All data points are presented as mean ± s.d, *N* ≥ 3. (f) Proposed model of rhodamine–Hoechst conjugate interaction with the target DNA. Minor groove binding results in a brighter complex compared to major groove binding. The docking results show 19 conformations of 610CP-Hoechst: all 5′-regioisomer conformers are positioned in the minor groove, 3 out of 19 6′-regioisomer conformers are positioned in the major groove. Docking models were built using the DNA structure taken from PDB ID: ; 8BNA.

The bisbenzimide core of Hoechst 33342 has been recently exploited as a DNA-targeting ligand for several classes of dyes.^4^,^6^,^14^ The general synthesis procedure of Hoechst derivatives consists of three steps: alkylation of Hoechst 33258 with Boc-protected 4-bromobutan-1-amine, protection group removal and coupling to the NHS ester of the corresponding dye (Scheme 1 and Fig. 1a). The overall yield of convergent synthesis was in the 20–40% range. To increase biocompatibility, we selected dyes that operate in the spectral range from 550 nm up to 650 nm and are compatible with STED nanoscopy. The isomerically pure 6′-regioisomers of the fluorophores were obtained using previously described regioselective synthesis procedures.^5^,^8^ Analogously, isomerically pure 5′-regioisomers of fluorescent dyes were obtained by adapting similar synthetic approaches by using protected 4-bromoisophthalic acid derivatives instead of the protected 2-bromoterephthalic acid intermediates (Schemes S1–S4†).

### Spectroscopic properties of the DNA stains

Both 5′- and 6′-carboxyrhodamine regioisomers have almost identical absorption, fluorescence emission spectra and quantum yield (QY) in phosphate buffered saline (PBS), with slightly higher quantum yields of 5′-regioisomer if 0.1% sodium dodecyl sulphate (SDS) surfactant is added (Table S1†). After coupling of dyes to the Hoechst moiety, we evaluated the spectroscopic properties of the new DNA probes by measuring their absorbance and fluorescence spectra as well as QY in PBS (Fig. 1a, b, S1 and Table S2†). Conjugation of the fluorophores to the Hoechst moiety induced a bathochromic shift of the absorbance, ranging from 11 to 23 nm, and the emission maxima, ranging from 1 to 28 nm, compared to the unconjugated dyes (Tables S1 and S2†). The QY after conjugation of the dyes was dramatically decreased (Table S2†). One possible explanation for such fluorescence quenching is self-aggregation of the lipophilic probes.^5^,^15^ Indeed, addition of 0.1% SDS recovers both the absorbance and the fluorescence, which is consistent with dissolving aggregates (Fig. S2 and Table S2†). The increase in fluorescence QY was much higher compared to the increase in the absorbance extinction coefficient confirming the main mechanism of fluorescence quenching by intermolecular aggregation and π–π interaction. We evaluated the aggregation extent by spinning down the aggregates with a bench-top centrifuge and measured the distribution of probes between the pellet and supernatant (Fig. S2†). We noticed that the 5′-regioisomer based probes are less prone to aggregation compared to 6′-regioisomer probes. The addition of DNA also recovers the absorbance and fluorescence of the probes, but to a lower extent compared to SDS. We assumed that the absorbance in PBS containing 0.1% SDS corresponds to the highest attainable absorbance for the probe in the aqueous solution, and we equated it to the extinction coefficients of free dyes (Tables S1 and S2†).

After addition of target DNA, the extinction coefficients close to the maximum were obtained for 580CP and 610CP probes (Tables S2 and S3†). In contrast, SiR and GeR dyes containing probes could recover approximately half of their maximal extinction. The QY for all probes in the presence of SDS was similar (0.5–0.6). However, the addition of target DNA yielded variable results: the highest QY (0.43) was recorded for **5-610CP-Hoechst** and the lowest (0.12) for **6-580CP-Hoechst**. Interestingly, all 5′-regioisomers showed a consistently higher QY compared to 6′-regioisomers. Comparison of the extinction coefficient and QY increase after target DNA addition indicated that most of the probe's fluorogenicity results from the change of QY (Table S3†). Finally, we calculated the brightness of each probe and identified the **5-610CP-Hoechst** probe as the brightest (Fig. 1c and Table 1).

**Table 1:** Properties of probe–hpDNA complexes

Probe name	*λ* abs max (nm)	*λ* em max (nm)	Brightness^*a*^ (M^–1^ cm^–1^)	Complex stability	
				*K*_d1_ (nM)	*K*_d2_ (nM)
**Hoechst 33342**	352	455	32 460 ± 5480	2.6 ± 0.4	—
**5-TMR-Hoechst**	558	586	11 300 ± 1700	365 ± 12	—
**6-TMR-Hoechst**	562	585	3210 ± 240	14 ± 1	168 ± 23
**5-580CP-Hoechst**	588	613	31 330 ± 1800	155 ± 14	—
**6-580CP-Hoechst**	594	619	9600 ± 830	27 ± 3	362 ± 37
**5-610CP-Hoechst**	614	641	36 400 ± 3230	347 ± 22	—
**6-610CP-Hoechst**	618	644	22 660 ± 1180	65 ± 7	4408 ± 1810
**5-GeR-Hoechst**	641	660	10 440 ± 1340	4374 ± 192	—
**6-GeR-Hoechst**	643	662	10 860 ± 850	572 ± 26	—
**5-SiR-Hoechst**	651	672	14 210 ± 1490	4799 ± 201	—
**6-SiR-Hoechst**	654	677	8500 ± 1690	669 ± 26	—

^*a*^Calculated using the equation: *ε* × QY; in PBS + hpDNA. Data presented as the mean value with standard deviation, *N* = 3.

### *In vitro* binding of Hoechst derivatives

We evaluated Hoechst conjugate's interaction with the target by measuring the fluorescence increase upon addition of hairpin DNA (hpDNA). Binding of Hoechst 33342 to hpDNA can be described with a single site binding model of *K*_d_ = 2.6 nM (Fig. 1d). We found that all 5′-regioisomers’ binding curves could be fitted to a single site binding model as well, but **6-TMR-Hoechst**, **6-580CP-Hoechst** and **6-610CP-Hoechst** displayed two site binding (Fig. 1d and e). The high affinity complex appears brighter than the low affinity complex resulting in the fluorescence increase at low concentrations followed by the decrease of signal at high concentrations of the target DNA (Fig. 1d, e and Table 1). To confirm dual mode binding, we measured the fluorescence lifetime of all Hoechst derivatives. Indeed, the fluorescence decay of Hoechst 33342 and all 5′-regioisomers could be fitted to a single exponent model (*τ* ∼ 2.9 ns), but all 6′-regioisomers, including **6-GeR-Hoechst** and **6-SiR-Hoechst**, displayed double exponential fluorescence decay (*τ*_1_ ∼ 3.1 ns and *τ*_2_ ∼ 1.1 ns) (Fig. S3 and Table S2†). Appearance of a shorter lifetime is consistent with the formation of a different lower QY complex. We hypothesize that in the case of **6-GeR-Hoechst** and **6-SiR-Hoechst**, we could not resolve two *K*_d_ in the titration experiment because they are too close to each other. In addition to binding to a minor groove, previous studies reported several alternative modes of Hoechst interaction with DNA – intercalation or major groove binding.7b,^11^,^16^ The docking experiments of Hoechst conjugates onto DNA suggest that in 5′-regioisomer probes minor groove binding predominates, while derivatives containing the 6′-regioisomer are also able to interact with the major groove (Fig. 1f and S4†). We assume that such an interaction was detected in the titration experiments as the second site binding event with ∼10-fold higher *K*_d_ and appears as the second fluorescence decay exponent in the lifetime measurements.

### Staining of the nuclei in living and fixed cells

**Fig. 2:**
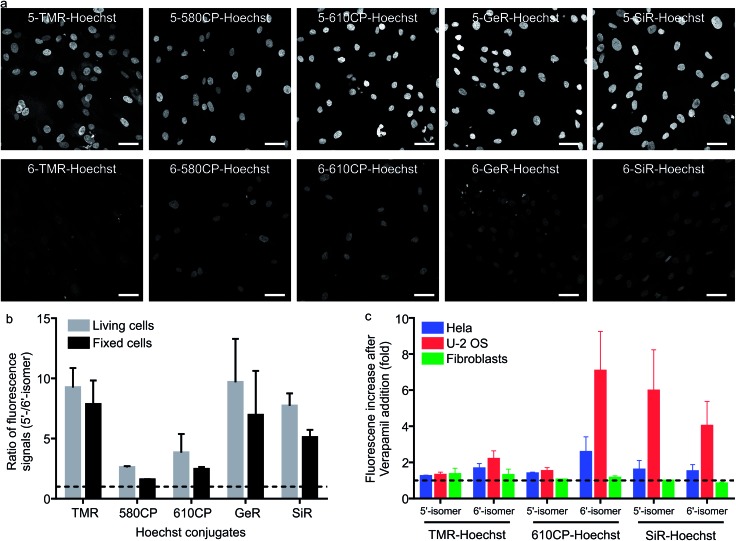
Performance of 5′-regioisomer and 6′-regioisomer probes. (a) Representative confocal images of human fibroblasts stained with 1 μM Hoechst derivatives for 1 h at 37 °C in DMEM growth medium. Cells were washed with HBSS and imaged in DMEM. Scale bars: 50 μm. (b) Quantification of the nuclear staining signal. Nuclei were automatically segmented and the signal of each staining was calculated by subtracting the mean fluorescence signal in the nucleus region from the narrow cytoplasmic area surrounding it. The median of the ratio of the fluorescence signal is presented. The dashed line indicates a ratio equal to 1. Bars show mean with the s.d. of three independent biological replicates; ≥50 cells per replicate. (c) Effect of efflux pump inhibition on nuclei staining. Cells were incubated with a mixture of 1 μM DNA probes and 10 μM verapamil in DMEM for 1 h at 37 °C, washed with HBSS, imaged in DMEM and the nuclear staining signal quantified. The dashed line indicates a ratio equal to 1. Bars show mean with the s.d. of three independent biological replicates; ≥50 cells per replicate.

Having characterized the DNA probe's properties *in vitro*, we moved to experiments in living cells. We stained human fibroblasts with Hoechst derivatives and found that the nuclei of cells stained with all 5′-regioisomers were up to 10-fold brighter compared to the 6′-regioisomer labelled nuclei (Fig. 2a and b). A similar situation was observed if cells were stained after methanol fixation (Fig. 2a and b), suggesting that plasma membrane permeability is not the major cause of the nuclei staining differences. Next, we investigated influence of efflux pumps on the performance of our probes. Cancer cell lines are known to express efflux pumps avoiding the toxic effect of anti-cancer drugs. We selected two human cancer cell lines: adenocarcinoma HeLa and osteosarcoma U-2 OS. Human fibroblasts served as the control cell line with minimal efflux activity. The probe efflux was inhibited by simultaneous addition of 10 μM verapamil – a broad spectrum efflux pump inhibitor.^17^ We observed that staining with **5**/**6-TMR-Hoechst** and **5-610CP-Hoechst** showed little dependency on the verapamil addition, suggesting little interference from efflux pumps. In contrast, **6-610CP-Hoechst** and both SiR-based probes appeared to be efficiently removed from the cells (Fig. 2c and S5†). Taken together, these results rule out the possibility that dramatically improved staining by 5′-regioisomer probes results from the impaired efflux or improved plasma membrane permeability. Instead, they are consistent with the absence of the second, dimmer complex, where the probe interacts with the DNA major groove. Even if this complex is of considerably lower affinity, the 6′-regioisomer probes can adopt lower QY conformation in the nucleus, where the concentration of DNA is extremely high, reaching up to 30 mM.^18^

### Cytotoxicity of the DNA probes

Cytotoxicity is an important parameter for all the fluorescent probes and depends on the concentration of the probe in the growth media. We monitored Hela population distribution over cell cycles after exposing them to several different concentrations of the DNA stains after 24 h (Fig. S6a†). Hoechst 33342 started to show cytotoxicity at concentration > 0.4 μM which was detected by accumulation of cells in S and sub G1 phases (Fig. S6b†). The most cytotoxic Hoechst derivatives were **5-TMR-Hoechst**, **6-580CP-Hoechst** and **6-610CP-Hoechst** (Fig. S6c†), while both regioisomers of GeR and SiR probes showed little toxicity (Fig. S6c†). Interestingly, the presence or absence of the second binding mode has a big effect on probe cytotoxicity. We found that, with the exception of **5-TMR-Hoechst**, 5′-regioisomer probes perturb the cell cycle much less than 6′-regioisomer probes. This can be explained by the docking results which show alternative major groove binding conformations. Most regulatory proteins recognizing specific DNA sequences interact with DNA from the major groove side,^19^ and thus probes binding to the same side will interfere significantly stronger with protein–DNA interaction. Furthermore, 5′-regioisomers will interfere less with the minor groove interacting proteins because of their lower affinity to the target DNA as compared to 6′-regioisomers (Table S4†). However, interactions with other biomolecules might also contribute to the cytotoxic effects as exemplified by the high cytotoxicity of **5-TMR-Hoechst**. Nevertheless, the more intense signal of the 5′-regioisomers allows achieving sufficient contrast in the images using a lower concentration of the probes. This offers the opportunity to use these probes at concentrations below the toxicity threshold and without addition of verapamil (Fig. S7†). To this end, we identified **5-610CP-Hoechst** as the brightest probe of low cytotoxicity whose staining is minimally affected by the efflux pumps.

### Super-resolution imaging of heterochromatin stained with Hoechst derivatives

**Fig. 3:**
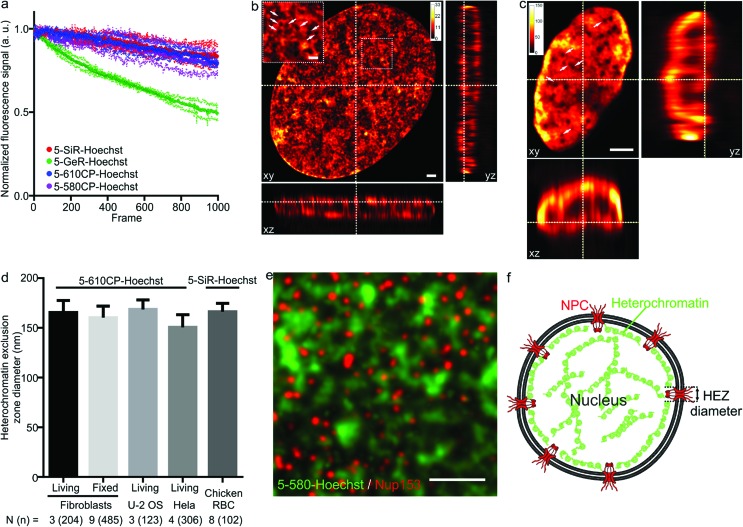
STED nanoscopy images of nuclei in living cells. (a) Photostability of the indicated Hoechst derivatives under STED imaging conditions measured on the nuclei of living cells stained with corresponding 1 μM DNA stain in DMEM growth medium at 37 °C for 1 h. Data presented as mean (large circles) with the s.d. (small circles), *N* = 3 nuclei. (b) *xy*, *xz* and *yz* slices of a deconvolved STED *z*-stack of a living fibroblast stained with 1 μM **5-610CP-Hoechst** in DMEM growth medium. The sample was washed once before imaging. Arrows indicate heterochromatin exclusion zones (HEZs). Scale bar: 1 μm in the large image and 0.5 μm in the inset. (c) *xy*, *xz* and *yz* slices of a deconvolved STED *z*-stack of an intact chicken red blood cell (RBC), stained with 1 μM **5-SiR-Hoechst** in RBC buffer. The sample was imaged without washing. Arrows indicate HEZs. Scale bar: 1 μm. (d) Comparison of the estimated HEZ diameters (see Materials and Methods) in the indicated cell types stained with **5-610CP-Hoechst** or **5-SiR-Hoechst**. See ESI† for details about the estimation of HEZ diameters. Data presented as mean with the s.d., *N* and *n* indicate the number of analyzed cells and zones, respectively. (e) Two-color STED nanoscopy of **5-580CP-Hoechst** stained and mouse anti-Nup153 antibody. Max intensity projection of 3 STED *z*-stack planes of a Hela cell nucleus stained with **5-580CP-Hoechst**, mouse anti-Nup153 and anti-mouse-Abberior STAR 635. Image was deconvolved with SVI Huygens software. Scale bar: 1 μm. (f) Proposed model of nuclear staining. Hoechst-based probes bind to the AT-rich heterochromatin after entering the nucleus. Heterochromatin is excluded from the location of nuclear pore complex (NPC) forming HEZs visible in the super-resolution microscopy images.

As illustrated by staining living human fibroblasts, the superior brightness of 5′-regioisomer probes allows achieving much brighter specific staining at probe concentrations well below the toxicity threshold, thus demonstrating their practical advantage over the 6′-regioisomers (Fig. S7†). Human fibroblasts stained with the best performing dyes – **5-580CP-Hoechst**, **5-610CP-Hoechst** and **5-SiR-Hoechst** – show significant improvement in resolving chromatin structures in STED nanoscopy images (Fig. S8†). The photostability of these dyes is sufficient to acquire more than 1000 STED frames and compatible with *z*-stack imaging (Fig. 3a). The analysis of *z*-stacks showed a strong fluorescence signal in the periphery of the nucleus. Hoechst 33342 is reported to stain AT-rich heterochromatin regions located close to the nuclear membrane.^20^ The nuclear pore complex protrudes into this layer of heterochromatin resulting in the exclusion zones. We could clearly observe heterochromatin exclusion zones in the peripheral regions of the nucleus after the deconvolution of images (Fig. 3b, c and S9–S11†). For the first time to our knowledge, the acquired *z*-stack of STED images allowed us to measure the diameter of HEZs to be ∼155 nm in living fibroblasts, Hela cells, U-2 OS cells and intact chicken erythrocytes (Fig. 3d). This example highlights the advantage of small molecule fluorescent probes being applicable to multiple cell types and species. We confirmed the nature of these exclusion zones by co-staining with an antibody against the nuclear pore complex marker Nup 153 (Fig. 3e, f and S12†).^21^ The estimated diameter of exclusion zones showed little variation depending on the probe and the cell type (Fig. 3d and Table S5†). We also imaged the time-course of the heterochromatin dynamics at prophase (Video S1†).

### Two color STED nanoscopy of living cells and intact erythrocytes

**Fig. 4:**
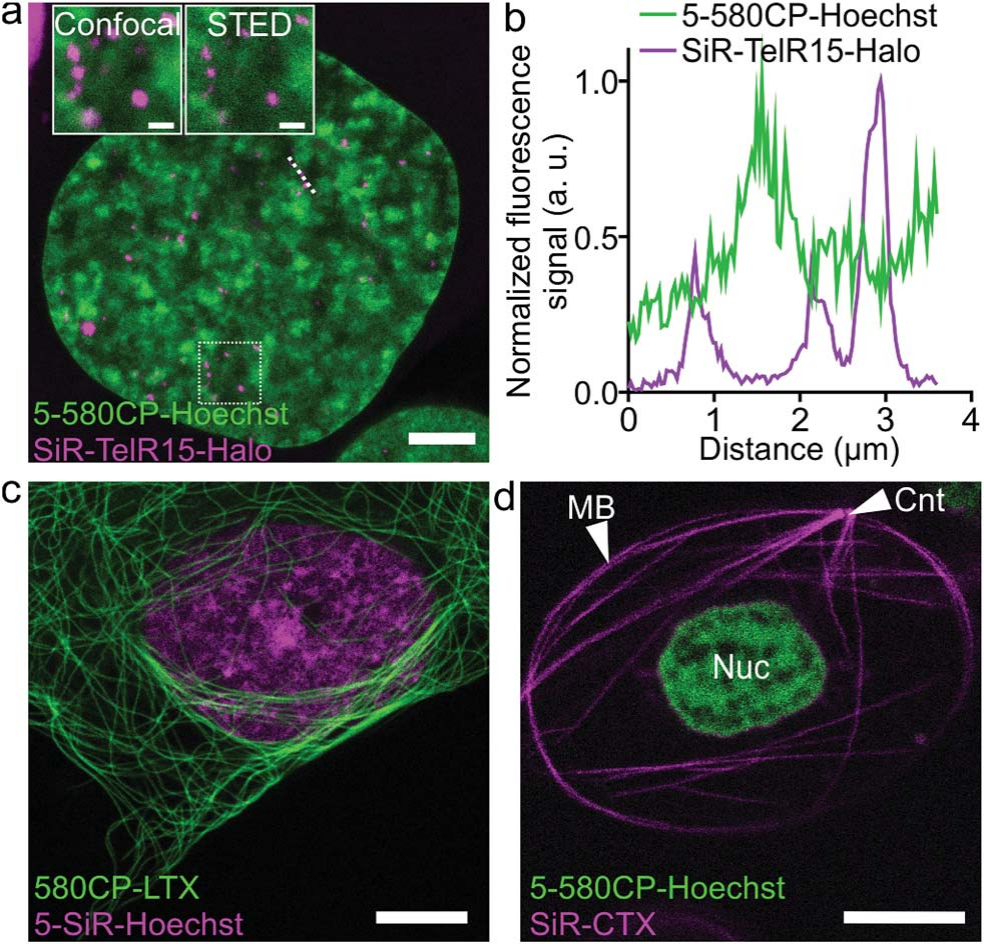
Two-color STED nanoscopy images of living cells. (a) Two-color STED image of U-2 OS cells expressing TelR15-Halo stained with 1 μM **5-580CP-Hoechst** and 1 μM SiR-Halo for 1 h at 37 °C in DMEM growth medium. The inset shows the zoom-in image of the region within a white rectangle. Scale bars: 5 μm in the large field of view and 1 μm in the inset. (b) Profile of fluorescence signals measured along the dotted line depicted in (a). TelR15-Halo and **5-580CP-Hoechst** signals are anti-correlating. (c) Two-color image of human fibroblasts stained with 1 μM 580CP-LTX (tubulin probe) and 1 μM **5-SiR-Hoechst** for 1 h at 37 °C in the complete DMEM growth medium. Scale bar: 5 μm. (d) Two-color image of a *Xenopus laevis* erythrocyte stained with 1 μM **5-580CP-Hoechst** and 1 μM SiR-CTX (tubulin probe) for 1 h at RT in RBC buffer. Note: CNT indicates the centrosome, MB – marginal band, and Nuc – nucleus. No washing steps were applied before imaging in RBC buffer. Scale bar: 5 μm.

The palette of characterized DNA stains allows setting up multicolor imaging experiments for standard and super-resolution microscopy. Several recently published examples showed a successful use of 580CP and SiR for two-color STED nanoscopy.8b,15a,^22^ We expressed Halo-tagged transcription activator-like effector (TALE) TelR15 and marked telomeric sequences in U-2 OS cells.^23^ Staining with SiR-Halo and **5-580CP-Hoechst** allowed localization of telomeres with respect to AT-rich regions of the cell nucleus (Fig. 4a and b). We observed that telomeres are excluded from regions stained by **5-580CP-Hoechst**. This observation is consistent with the fact that telomeres are GC-rich.^24^

Many highly specialized cell lines are sensitive to the transfection reagents, and show low transfection efficiency and large transgene expression level variations.^25^ For example, primary fibroblasts or erythrocytes of various species expressing tagged proteins cannot be easily obtained. We were able to acquire two-color STED nanoscopy *z*-stacks of images of the human primary fibroblasts and the nucleated erythrocytes from chicken (*Gallus gallus*) and frog (*Xenopus laevis*)^26^ stained with **5-SiR-Hoechst** and our recently developed tubulin probe 580CP-LTX (Fig. 4c, d and S13†).15*a* For erythrocyte imaging, we exploited 580CP excitation at 561 nm, which targets haemoglobin absorbance minimum allowing minimal cell perturbations. The excitation at 640 nm, STED laser at 775 nm and emission of both dyes are positioned outside the haemoglobin absorbance region (Fig. S13a†). Such a configuration allowed us to obtain high quality two-color STED nanoscopy images of DNA and tubulin structures in intact chicken and frog erythrocytes (Video S2†). We could easily identify the nucleus and the marginal band composed of microtubules in both species (Fig. S13b, c and Video S2†). In addition, the centrosome could be easily identified in the frog erythrocytes.^27^ For the first time, this example demonstrates the feasibility of two-color STED nanoscopy in intact erythrocytes containing high haemoglobin concentrations. The newly generated DNA probes show wide applicability to different types of cells and excellent performance even in extremely difficult to image cell types such as erythrocytes.

## Conclusions

In conclusion, our study demonstrates – for the first time to our knowledge – that the choice of the carboxyrhodamine regioisomer can influence the performance of the final fluorescent probe by changing its interaction with the target DNA. We show that probes with 5′-regioisomers bind DNA with lower affinity, but yield up to 10-fold brighter nuclear staining in living and fixed cells and are less cytotoxic than probes with 6′-isomers. This is explained by the absence of the lower affinity interaction with the DNA major groove, which in the case of the 6′-regioisomer-containing DNA probes considerably decreases staining brightness and is associated with higher cytotoxicity. The generality of this observation is supported by the same trend observed with all tested dyes: 5′-regioisomers always appear brighter compared to 6′-regioisomers. Our work strongly underlines the importance of including both structural isomers of carboxyrhodamines in the optimization step of fluorescent probes for living cell imaging.

## Conflicts of interest

G. L. has filed a patent application on the SiR fluorophore. S. W. H. owns shares in Abberior Instruments GmbH manufacturing Abberior STED 775 QUAD scanning microscopes.

## Supplementary Material

Supplementary information

Supplementary movie

Supplementary movie
