# Supplementary material for: Rhodamine–Hoechst positional isomers for highly efficient staining of heterochromatin
Source: Chem Sci. 2018 Dec 12;10(7):1962–70. doi: 10.1039/c8sc05082a (PMC6385482; doi:10.1039/c8sc05082a)

Electronic Supplementary Material (ESI) for Chemical Science.
This journal is © The Royal Society of Chemistry 2018

Supplementary information

Rhodamine-Hoechst positional isomers for highly efficient staining of heterochromatin

Jonas Bucevičius, Jan Keller-Findeisen, Tanja Gilat, Stefan W. Hell and Gražvydas Lukinavičius*

Max Planck Institute for Biophysical Chemistry, Department of NanoBiophotonics, Am Fassberg 11, 37077 Göttingen, Germany

*e-mail: grazvydas.lukinavicius@mpibpc.mpg.de

Supplementary inventory

Supplementary Schemes and figures 4
Scheme S1. Synthetic route of 5-SiR-COOH dye. 4
Scheme S2. Synthetic route of 5-610CP-COOH dye. 4
Scheme S3. Synthetic route of 5-580CP-COOH dye. 5
Scheme S4. Synthetic route of 5-GeR-COOH dye. 6
Figure S1. Spectral properties of the DNA probes. 7
Figure S2. Quantification of the Hoechst derivatives aggregation. 8
Figure S3. Fluorescence decay pofiles of 610CP-Hoechst regioisomers. 9
Figure S4. Models of TMR- and 580CP-Hoechst probes docked to the target DNA. 10
Figure S5. Confocal images of living (a) Hela or (b) U-2 OS cells stained with the fluorescent DNA probes. 11
Figure S6. Cell cycle perturbation induced by fluorescent DNA probes. 12
Figure S7. Probe concentration influence on the DNA staining in human fibroblasts. 13
Figure S8. Confocal and STED (775 nm) images of living human fibroblasts nuclei stained with the Hoechst derivatives. 14
Figure S9. Sections through STED z-stack of nuclei of fibroblasts (a) and intact chicken erythrocyte (b). 15
Figure S10. Sections through STED z-stacks of nuclei of Hela cells stained with 5-SiR-Hoechst (a,b) or with 5-610CP-Hoechst (c,d). 16
Figure S11. Sections through STED z-stacks of nuclei of U2OS cells nuclei with 5-SiR-Hoechst (a,b) and with 5-610CP-Hoechst (c,d). 17
Figure S12. Two-color STED nanoscopy of 5-580CP-Hoechst stained and mouse anti-Nup153 antibody. 18
Figure S13. Two-color STED nanoscopy of avian and frog erythrocytes. 19
Supplementary Movies 20
Video S1. Timelapse movie of the nucleus in living U-2 OS cell. 20
Video S2. Rotating maximum intesity projection of the intact Xenopus laevis erythocyte. 20
Supplementary Tables 21
Table S1. Photophysical properties of rhodamine fluorescent dyes used in the study. 21
Table S2. Photophysical properties of DNA probes. 22
Table S3. Absorbance and fluorescence changes of DNA probes upon interaction with DNA or SDS 23
Table S4. Cytotoxicity of Dye-Hoechst DNA probes 23
Table S5. Heterochromatin exclusion zone diameter near the nuclear pore complexes. . 23
Computation, molecular biology and biochemical methods 24
Estimation of the probe aggregation 24
Preparation of hairpin DNA 24
Plasmid construction 24
Maintenance and preparation of cells 24
Preparation of erythrocytes 25
Cell cycle analysis by imaging flow cytometry 25
Processing and visualization of acquired images 26
General experimental information and synthesis 28
2,2'-(4-bromo-1,3-phenylene)bis(4,4-dimethyl-4,5-dihydro-1,3-oxazole) (SI-1) 28
5-SiR-COOH 29
5-610CP-COOH 30
Di-*tert*-butyl 4-bromoisophtalate (SI-4) 30
Tert-butyl 3,6-bis{[*tert*-butyl(dimethyl)silyl]oxy}-10,10-dimethyl-3'-oxo-3'*H*,10*H*-spiro[anthracene-9,1'-[2]benzofuran]-5'-carboxylate (SI-6) 31
Tert-butyl 3,6-dihydroxy-10,10-dimethyl-3'-oxo-3'*H*,10*H*-spiro[anthracene-9,1'-[2]benzofuran]-5'-carboxylate (SI-7) 31
Tert-butyl 10,10-dimethyl-3'-oxo-3,6-bis[(trifluoromethanesulfonyl)oxy]-3'*H*,10*H*-spiro[anthracene-9,1'-[2]benzofuran]-5'-carboxylate (SI-8) 32

Tert-butyl 3,6-bis[(*tert*-butoxycarbonyl)(methyl)amino]-10,10-dimethyl-3'-oxo-3'*H*,10*H*-spiro[anthracene-9,1'-[2]benzofuran]-5'-carboxylate SI-9 32
5-580CP-COOH 33
5-GeR-COOH 33
Tert-butyl (3-{4-[5-(4-methylpiperazin-1-yl)-1H,1'H-[2,5'-bibenzimidazol]-2'-yl]phenoxy}butyl)carbamate 34
4-{4-[5-(4-methylpiperazin-1-yl)-1*H*,1'*H*-[2,5'-bibenzimidazol]-2'-yl]phenoxy}butan-1-amine (SI-13) 35
General procedure for the Dye-Hoechst conjugates: 35
6-580CP-Hoechst 36
5-580CP-Hoechst 36
6-TMR-Hoechst 37
5-TMR-Hoechst 38
6-SiR-Hoechst 38
5-SiR-Hoechst 39
6-CP610-Hoechst 40
5-CP610-Hoechst 40
6-GeR-Hoechst 41
5-GeR-Hoechst 42
Supplementary references 43
NMR copies 44

Supplementary Schemes and figures

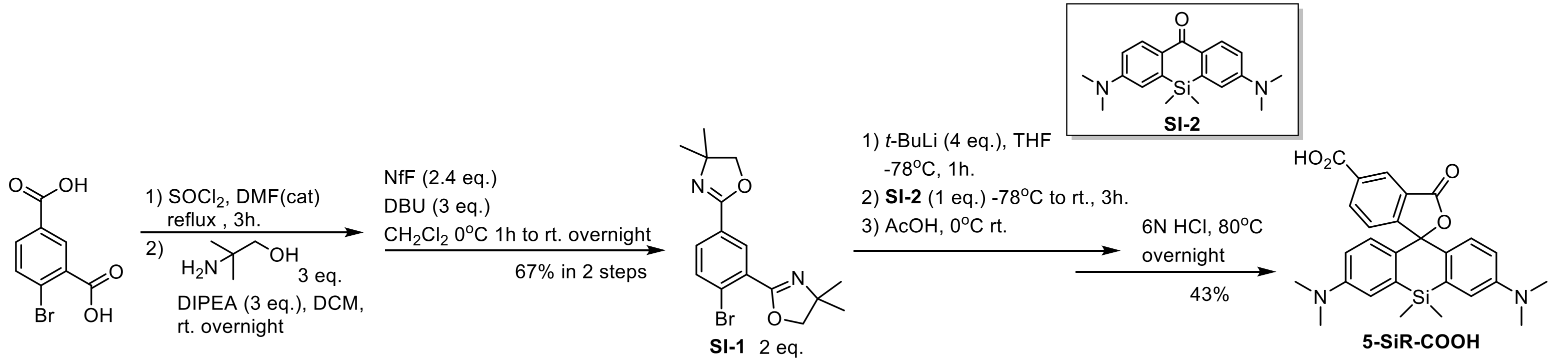

Scheme S1. Synthetic route of 5-SiR-COOH dye.

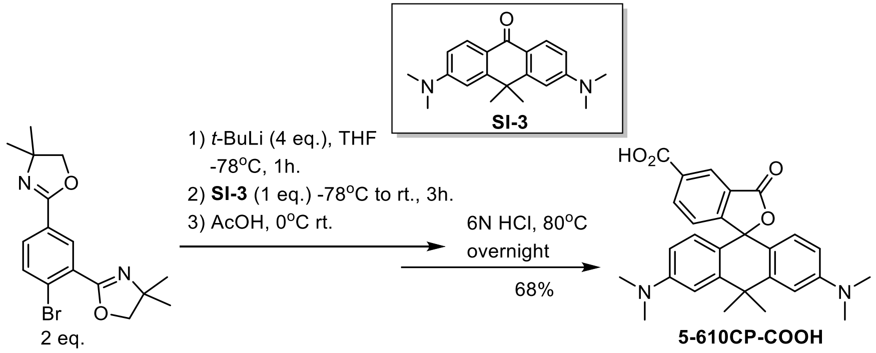

Scheme S2. Synthetic route of 5-610CP-COOH dye.

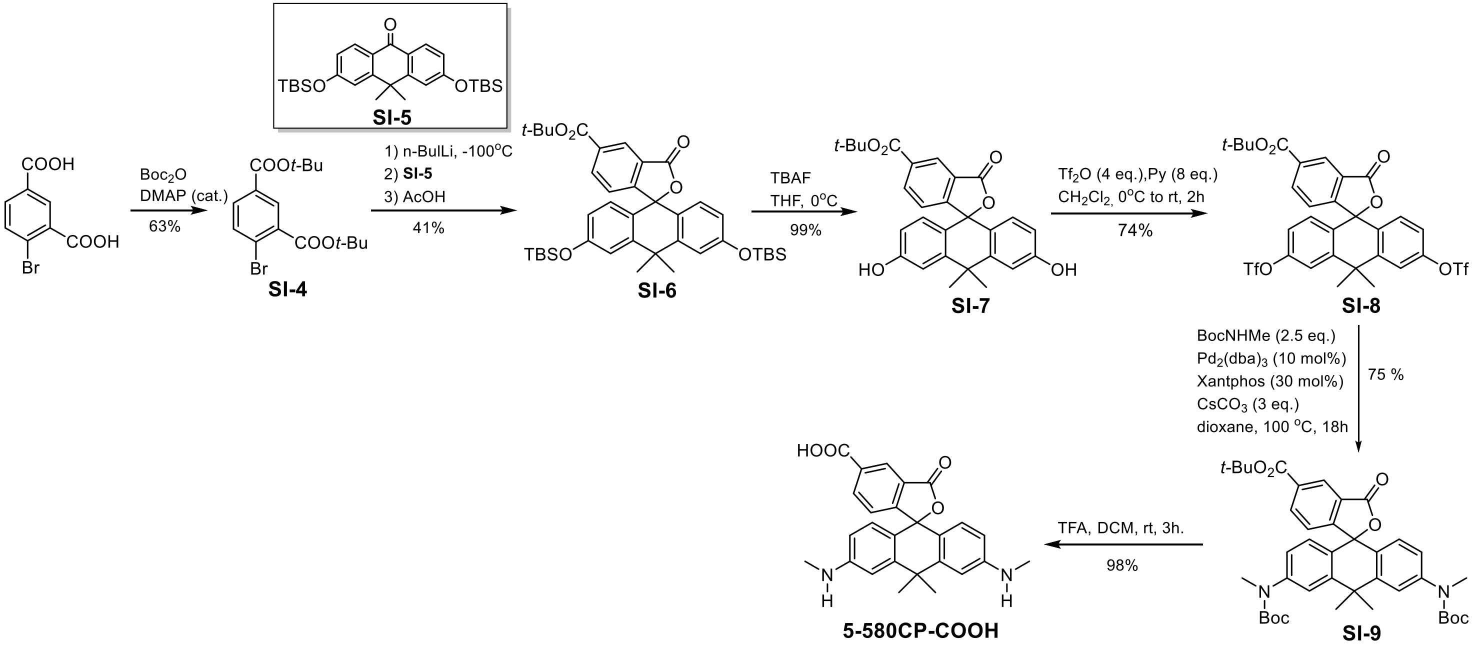

Scheme S3. Synthetic route of 5-580CP-COOH dye.

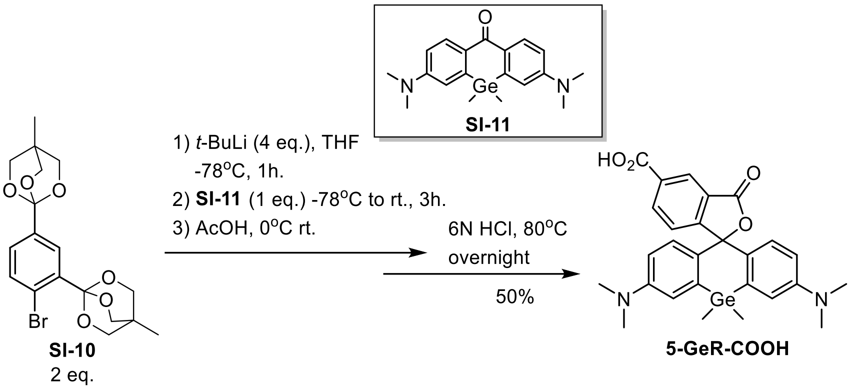

Scheme S4. Synthetic route of 5-GeR-COOH dye.

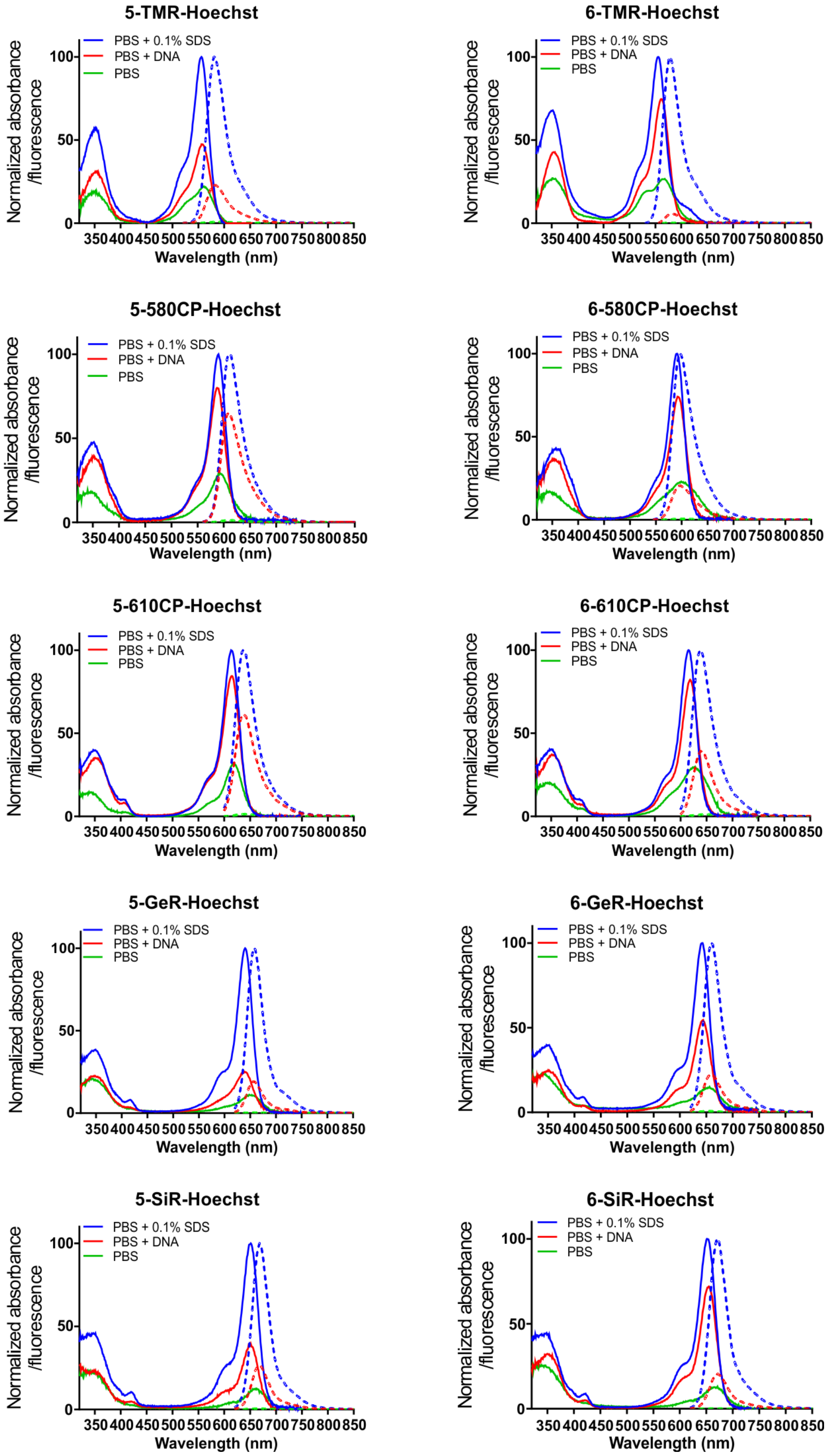

Figure S1. Spectral properties of the DNA probes. Absorbance (solid line) and fluorescence (dotted line) spectra of DNA probes in PBS (green), PBS containing 30 µM hpDNA oligonucleotide (red) and PBS containing 0.1% SDS (blue). 2 µM probes were incubated at room temperature for 2 h before measurements. Spectra are normalized to the samples containing 0.1% SDS and presented as averages of three independent experiments.

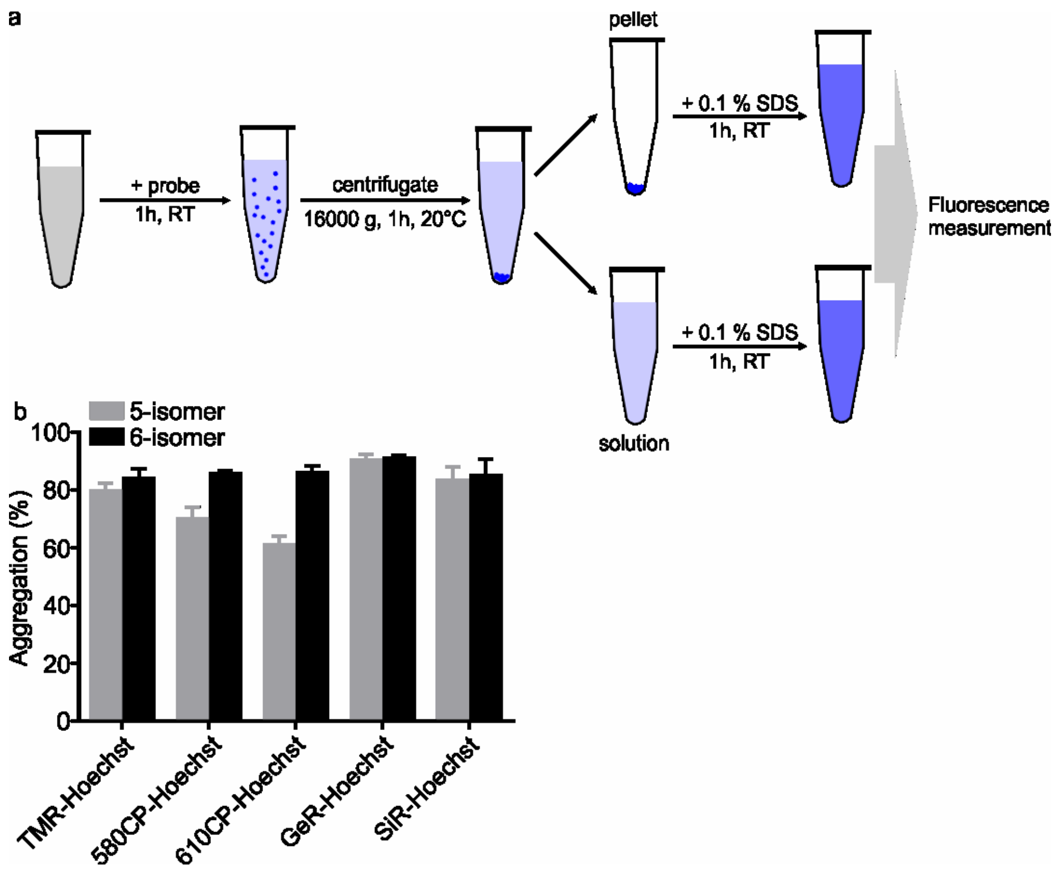

Figure S2. Quantification of the Hoechst derivatives aggregation. (**a**) The principle of assay used for aggregation extent estimation. (**b**) Measured aggregation of DNA stains. Data presented as mean with s. d., N = 3.

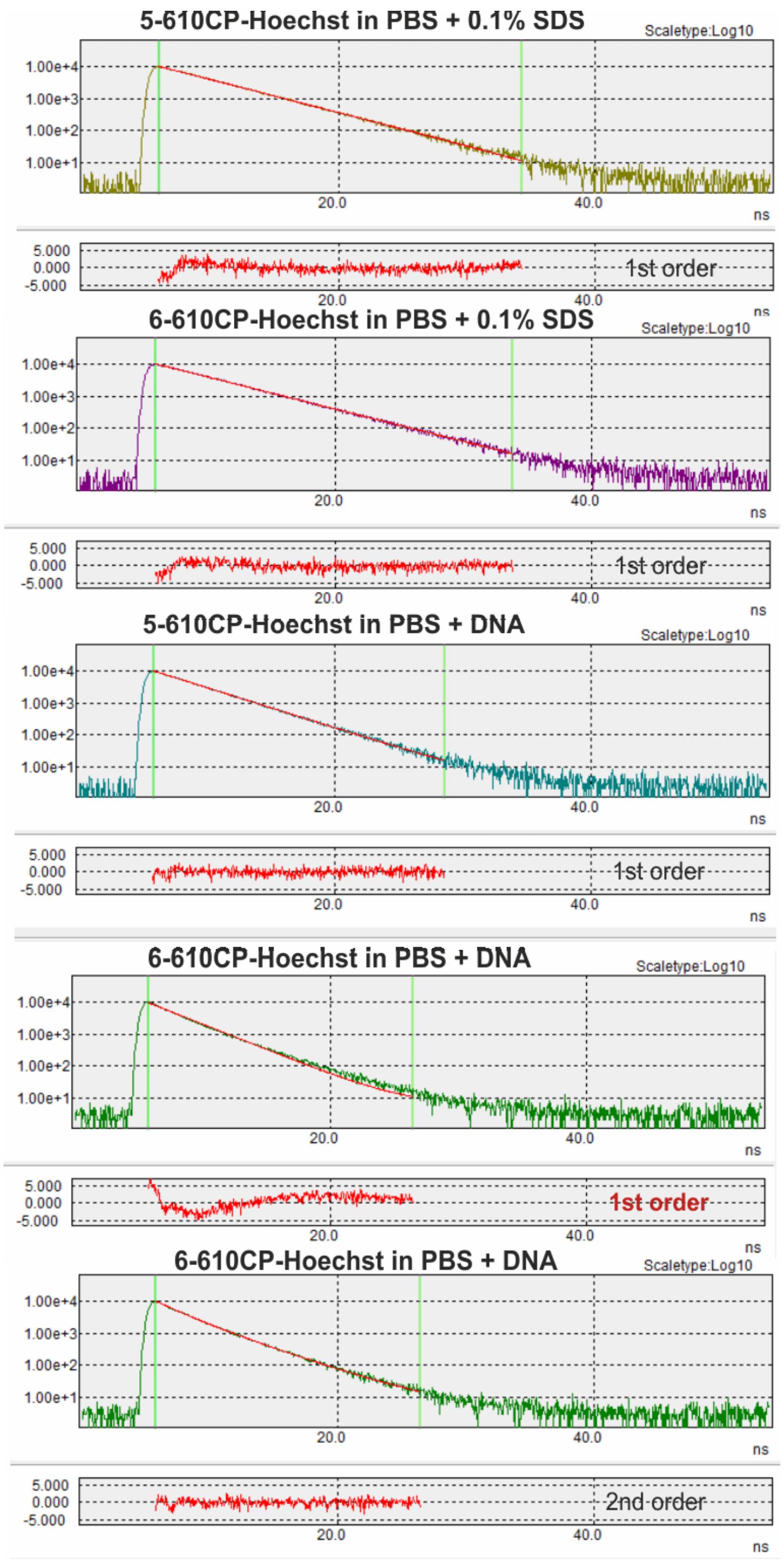

Figure S3. Fluorescence decay pofiles of 610CP-Hoechst regioisomers. Fluorescence lifetime of 5-610CP-Hoechst and 6-610CP-Hoechst recorded in PBS + 0.1% SDS and PBS + DNA. Data points were fitted to the first or second order exponential decay functions. The residuals from a fitting are shown below each fitting.

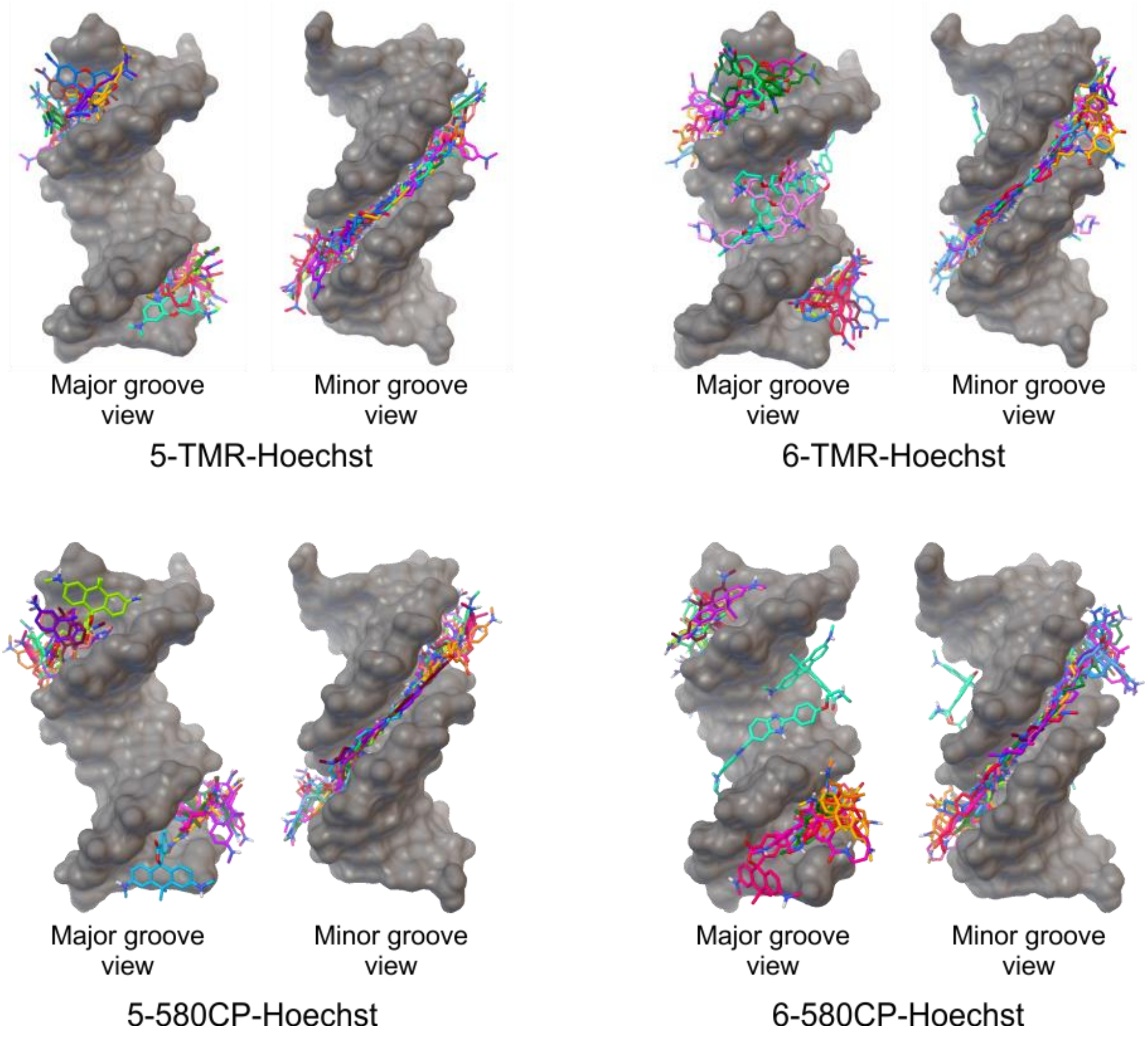

Figure S4. Models of TMR- and 580CP-Hoechst probes docked to the target DNA. Coordinates taken from PDB ID: 8BNA[1]. Docking result shows possible conformations of the probes: all conformers of isomer-5 are positioned in the minor groove, but some of the isomer-6 conformers are predicted to be positioned in the major groove.

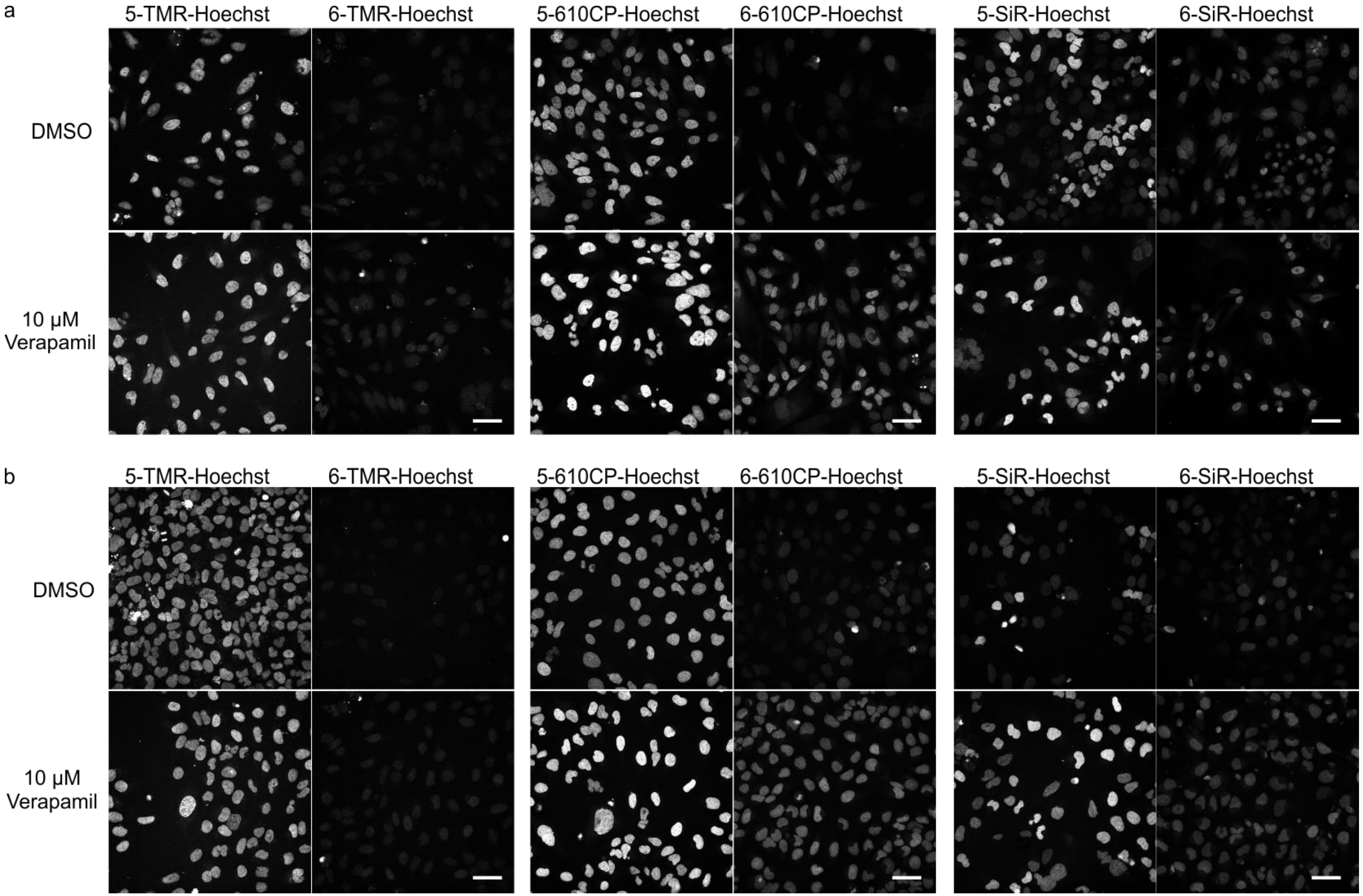

Figure S5. Confocal images of living (a) Hela or (b) U-2 OS cells stained with the fluorescent DNA probes. Living cells were stained with 1 µM DNA probe alone or a mixture of DNA probe and 10 µM Verapamil in DMEM growth medium containing 10% FBS. The cells were incubated at 37 °C for 1 h and washed once with HBSS and imaged using the same excitation powers for the four represented images: two isomers with and without Verapamil. Scale bars: 50 µm.

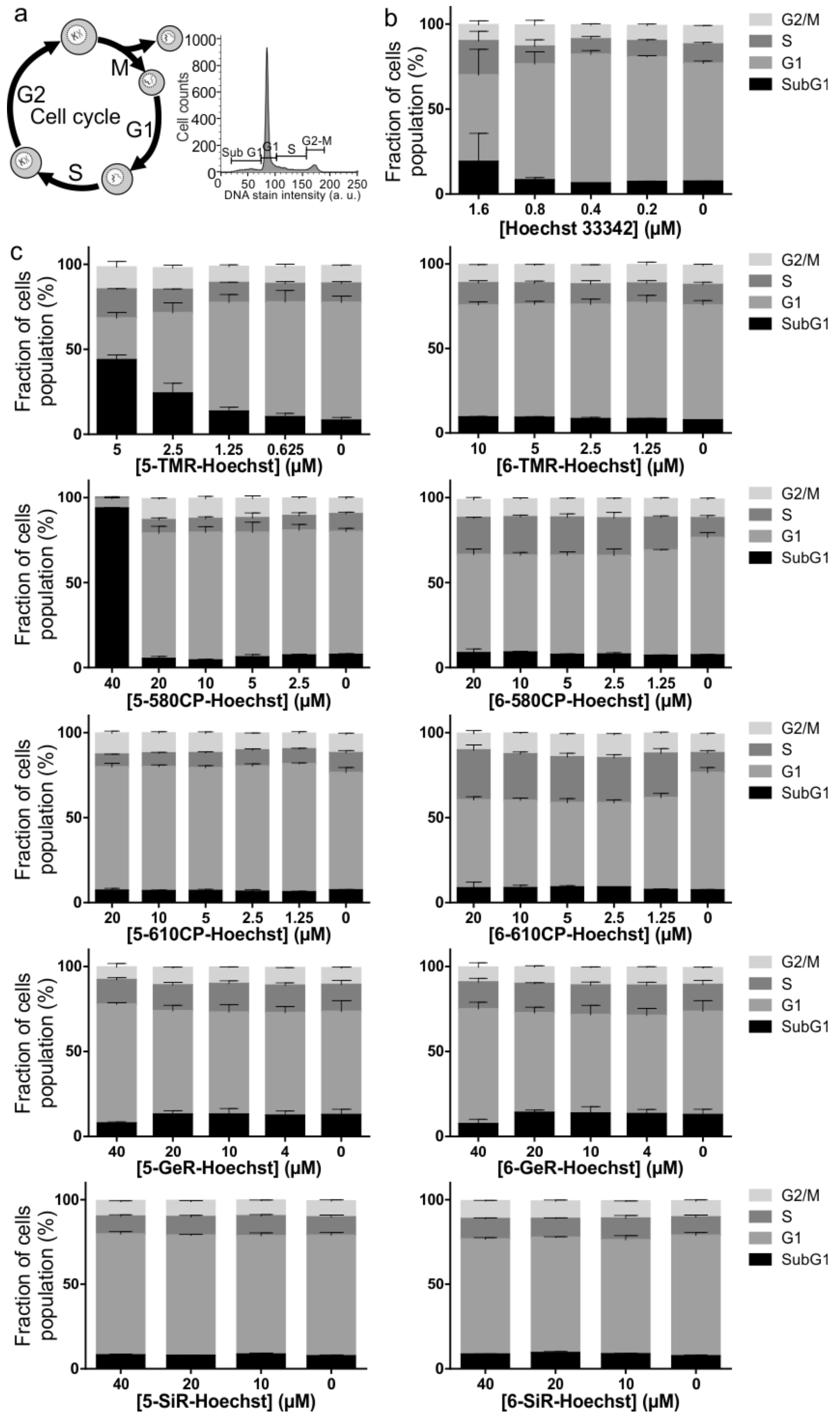

Figure S6. Cell cycle perturbation induced by fluorescent DNA probes. (a) On the left, cytotoxicity of DNA probes results from the interference with DNA synthesis and/or protein-DNA complex formation. This can induce DNA damage responses and G2 arrest. On the right, A representative histogram of DNA content distribution in Hela cell populations treated with DMSO only. Indicated cell cycle phases (Sub G1, G1, S and G2-M) are identified by the amount of DNA in the measured cells. (b) Outcome of control experiment when cells were treated with the indicated concentrations of Hoechst 33342. (c) Cytotoxicity measurements of DNA probes. Hela cells were incubated with the indicated concentrations of the DNA probes at 37 °C for 24 h in a humidified 5% CO2 incubator. Experimental data are averages of the three independent experiments (N=3) and presented as means with standard deviations.

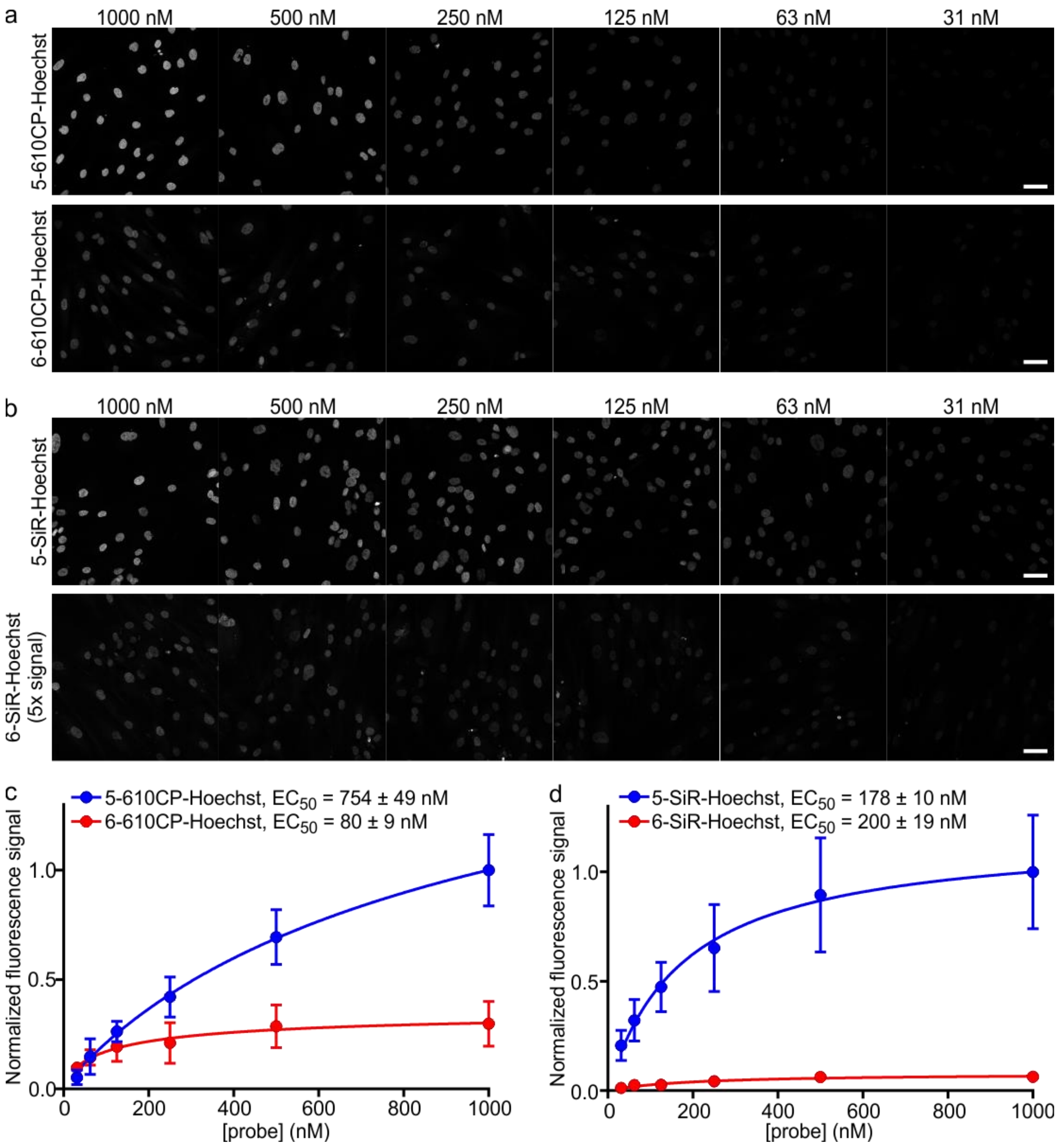

Figure S7. Probe concentration influence on the DNA staining in human fibroblasts. (a) Confocal images of living human fibroblasts stained with the indicated concentrations of 5-610CP-Hoechst or 6-610CP-Hoechst probes in DMEM at 37°C for 1h. Cells were washed once with HBSS and imaged in DMEM. Scale bar 50 µm. (b) Confocal images of living human fibroblasts stained with the indicated concentrations of 5-SiR-Hoechst or 6-SiR-Hoechst probes in DMEM at 37°C for 1h. Cells were washed once with HBSS and imaged in DMEM. Note that fluorescence signal is multiplied by 5 in the 6-SiR-Hoechst images. Scale bar 50 µm. Quantification of fluorescence signal of the stained nucleus in living cells stained with (c) 610CP-Hoechst probes or (d) SiR-Hoechst probes. Normalized fluorescence signal data points are presented as means with standard deviations, N > 50 measured nuclei for each condition in three different fields of view. Data points are fitted to the three parameter dose response equation using GraphPad Prism 6.0.

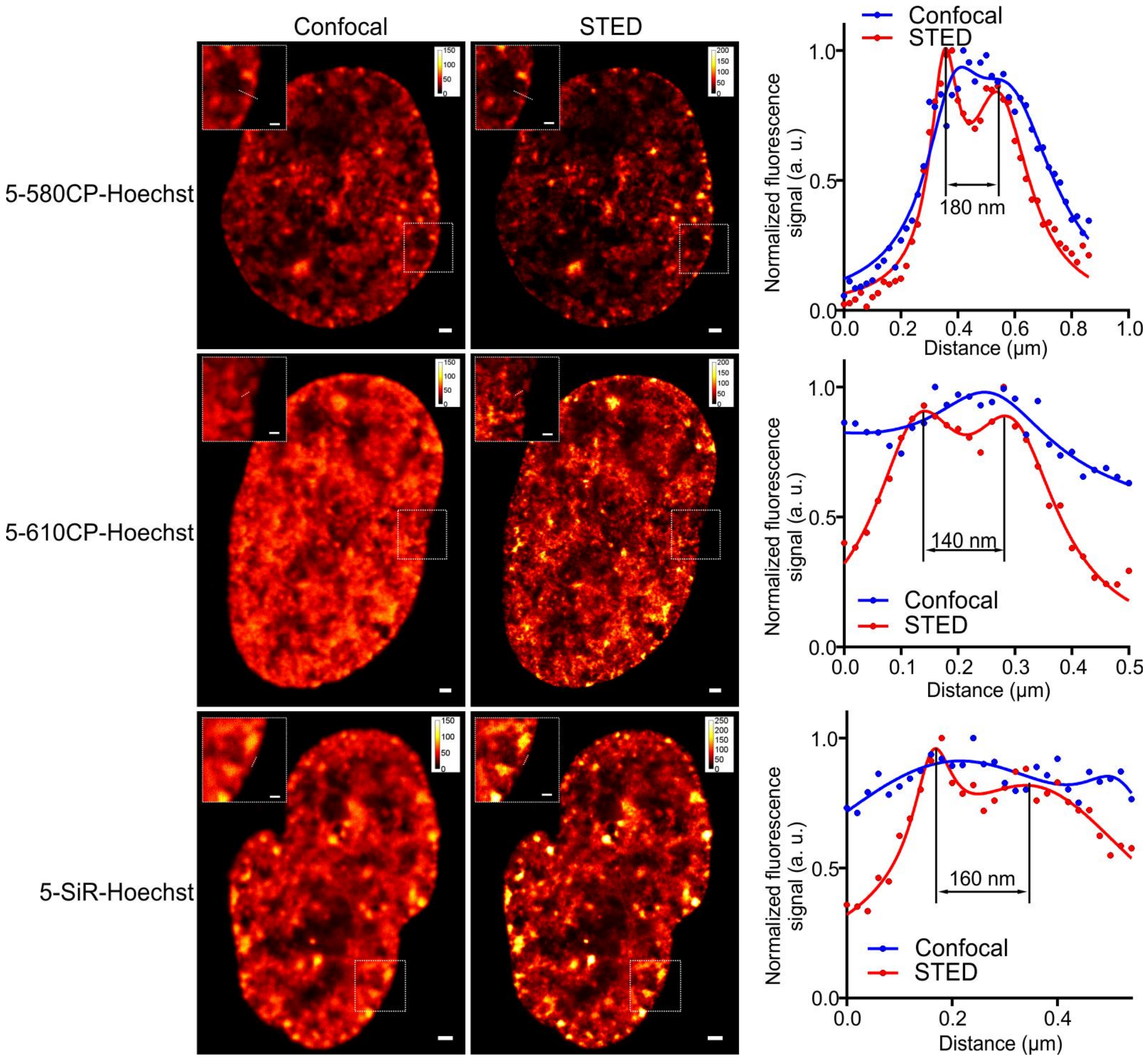

Figure S8. Confocal and STED (775 nm) images of living human fibroblasts nuclei stained with the Hoechst derivatives. Living human fibroblasts were incubated with 1 µM DNA probes in DMEM at 37 °C for 1 h, washed once with HBSS and imaged. Fluorescence signal profiles shown on the right are taken at the positions indicated in the corresponding inserts in the images on the left by the dotted lines. Scale bars: 1 µm in the large field of view and 0.5 µm in the zoom-in insert.

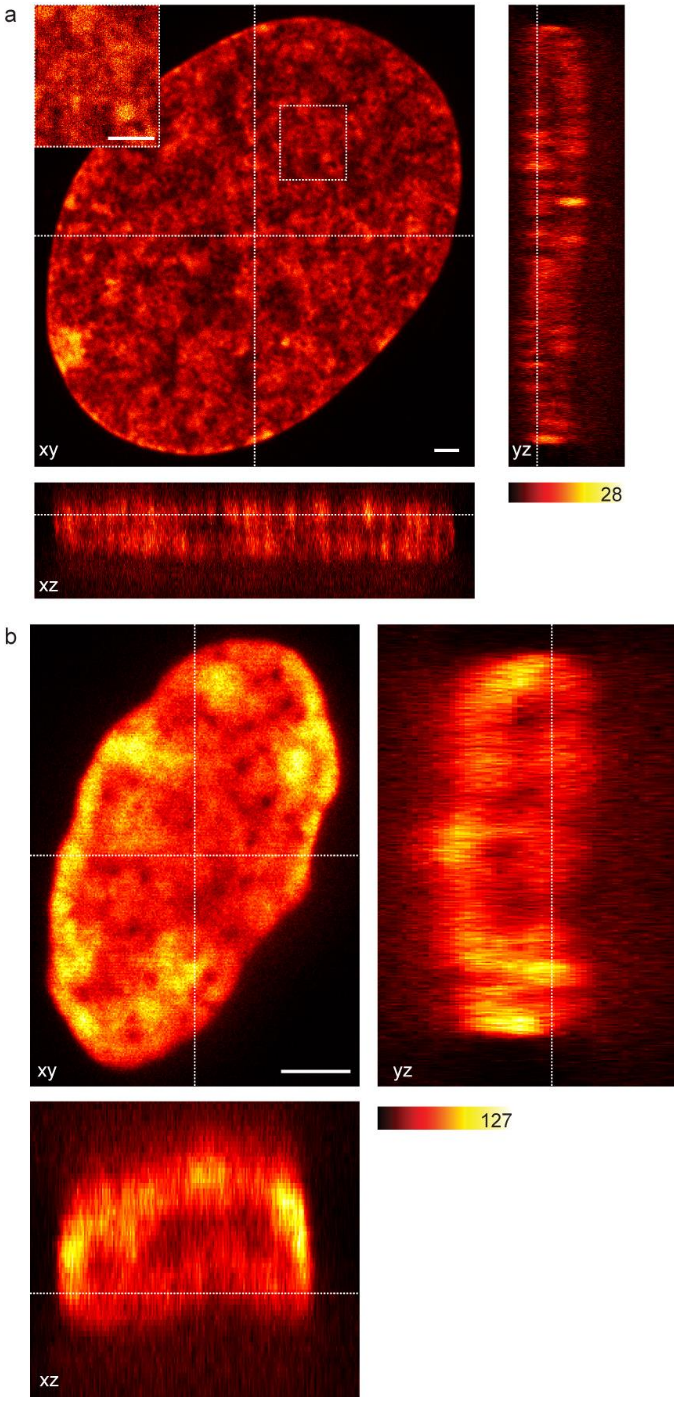

Figure S9. Sections through STED z-stack of nuclei of fibroblasts (a) and intact chicken erythrocyte (b). Fibroblasts were stained with 1 µM 5-610CP-Hoechst in DMEM growth media for 1 h at 37°C and washed once with HBSS before imaging in DMEM growth media. Chicken RBC stained with 1 µM 5-SiR-Hoechst and imaged in RBC buffer without washing. Scale bars 1µm.

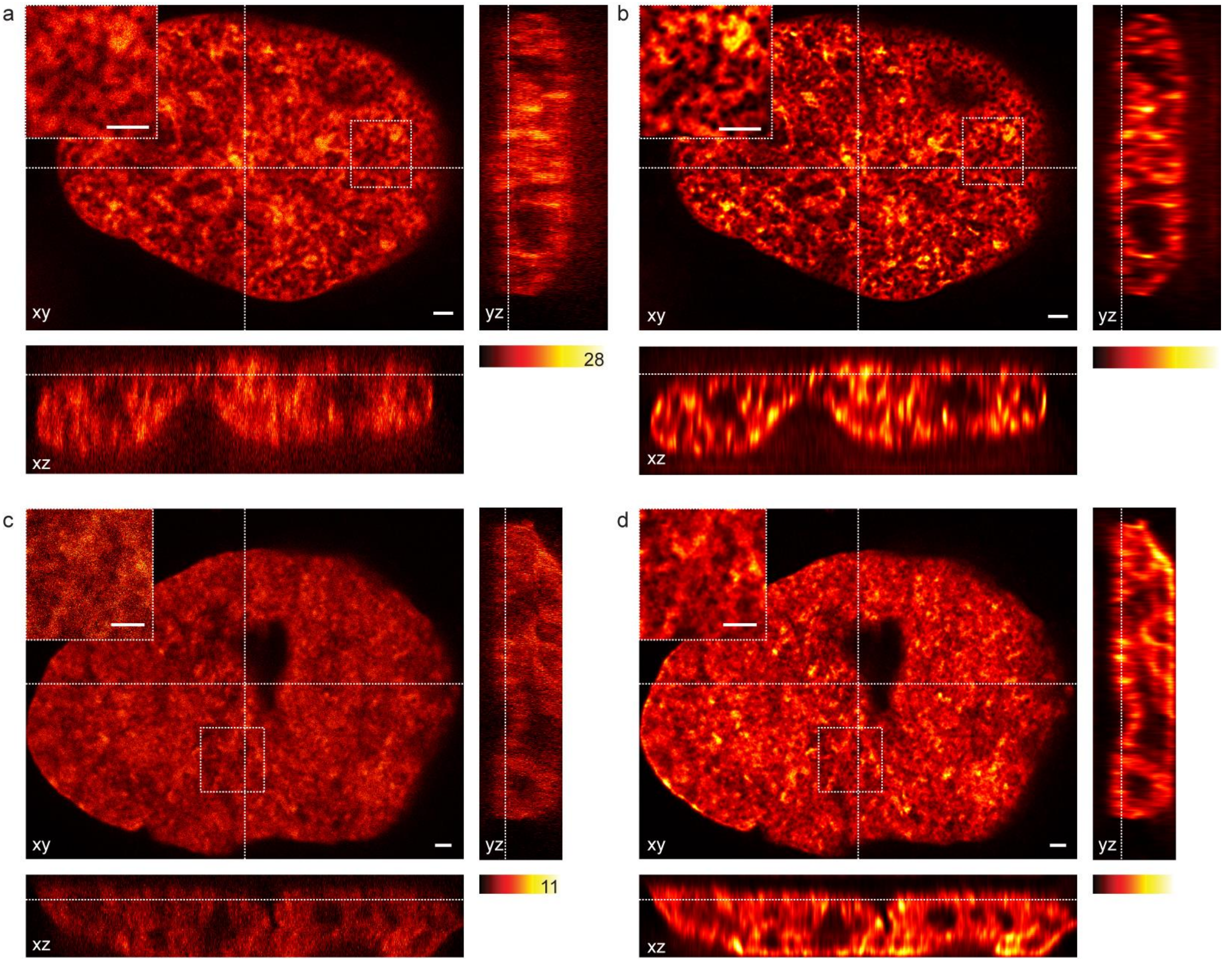

Figure S10. Sections through STED z-stacks of nuclei of Hela cells stained with 5-SiR-Hoechst (a,b) or with 5-610CP-Hoechst (c,d). (a,c) Raw data after drift correction, (b,d) Deconvolved data, Zooms in left upper corners of the xy images are taken from the respective dotted rectangle. The dotted lines indicate locations of the xy, xz, yz sections within the other sections. Cells stained with 1 µM probes in DMEM growth media for 1 h at 37°C and washed once with HBSS before imaging in DMEM growth media. Scale bars 1µm.

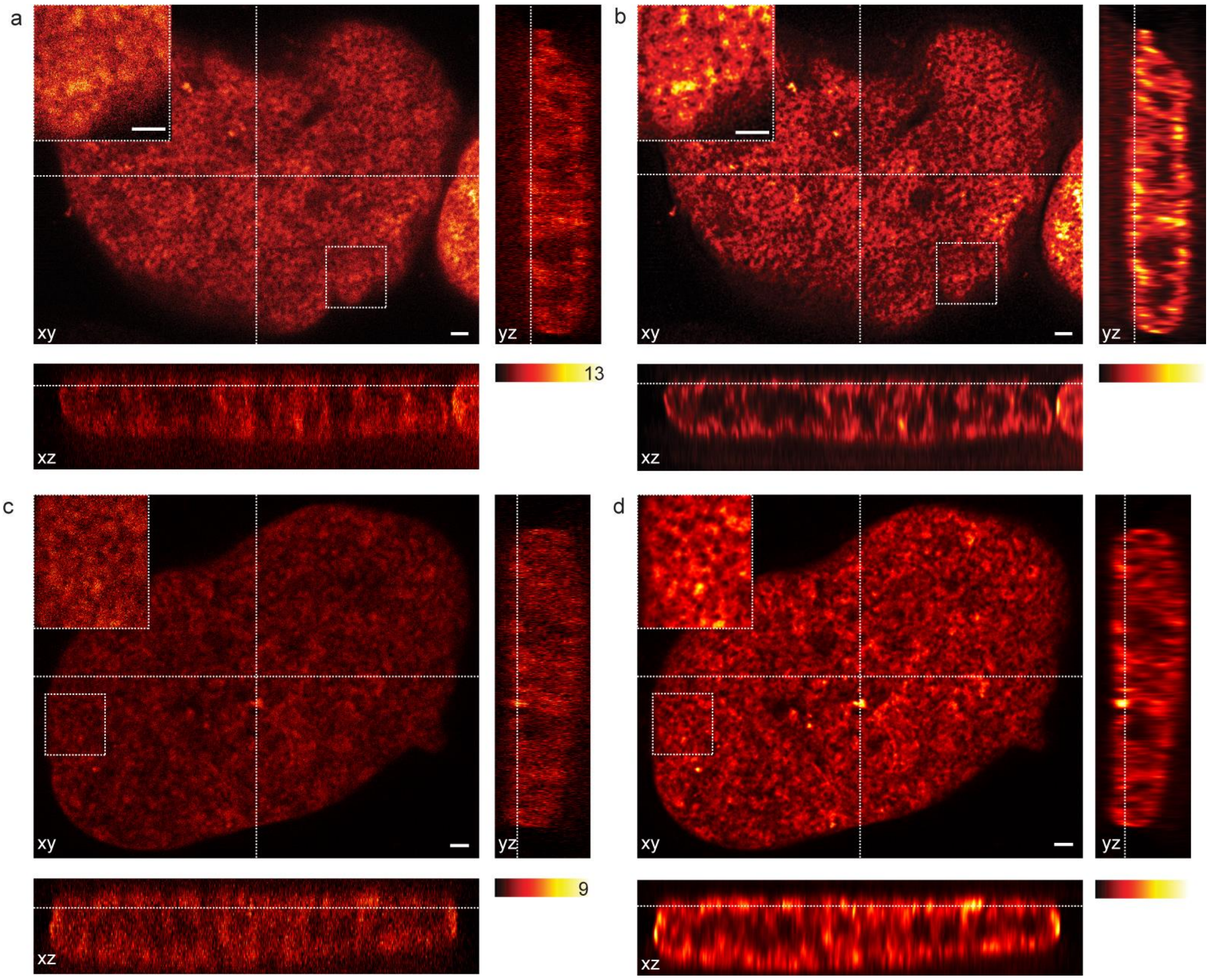

Figure S11. Sections through STED z-stacks of nuclei of U2OS cells nuclei with 5-SiR-Hoechst (a,b) and with 5-610CP-Hoechst (c,d). (a,c) Raw data after drift correction, (b,d) Deconvolved data, Zooms in left upper corners of the xy images are taken from the respective dotted rectangle. The dotted lines indicate locations of the xy, xz, yz sections within the other sections. Cells stained with 1 µM probes in DMEM growth media for 1 h at 37°C and washed once with HBSS before imaging in DMEM growth media. Scale bars 1µm.

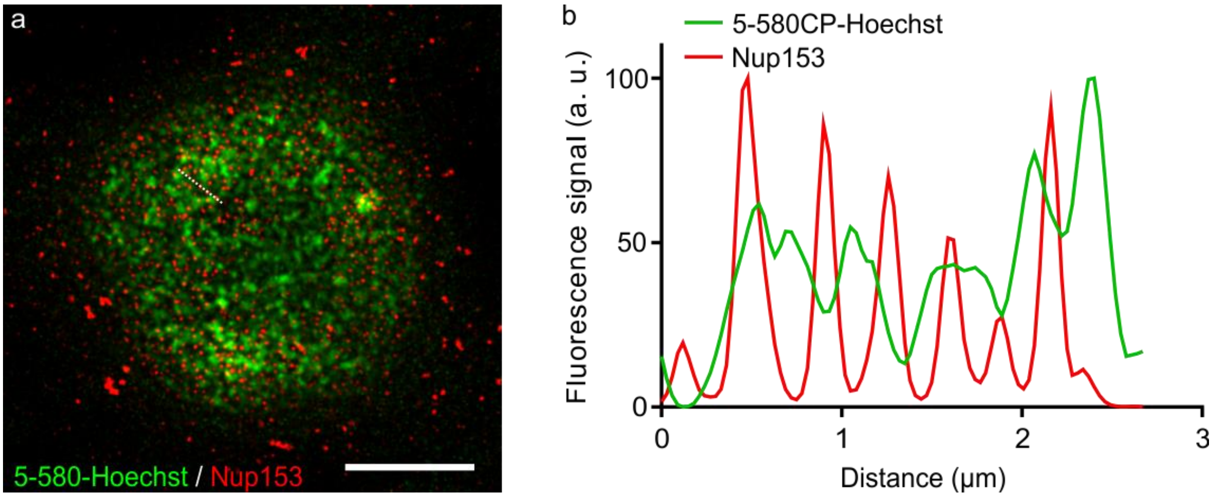

Figure S12. Two-color STED nanoscopy of 5-580CP-Hoechst stained and mouse anti-Nup153 antibody. (a) Max intensity projection of 3 STED z-stack planes of Hela cell nucleus stained with 5-580CP-Hoechst, mouse anti-Nup153 and anti-mouse-Abberior STAR 635. Image was deconvolved with SVI Huygens software. Scale bar 5 µm. (b) Line profile of fluorescence signal measured along the dotted line depicted in (a).

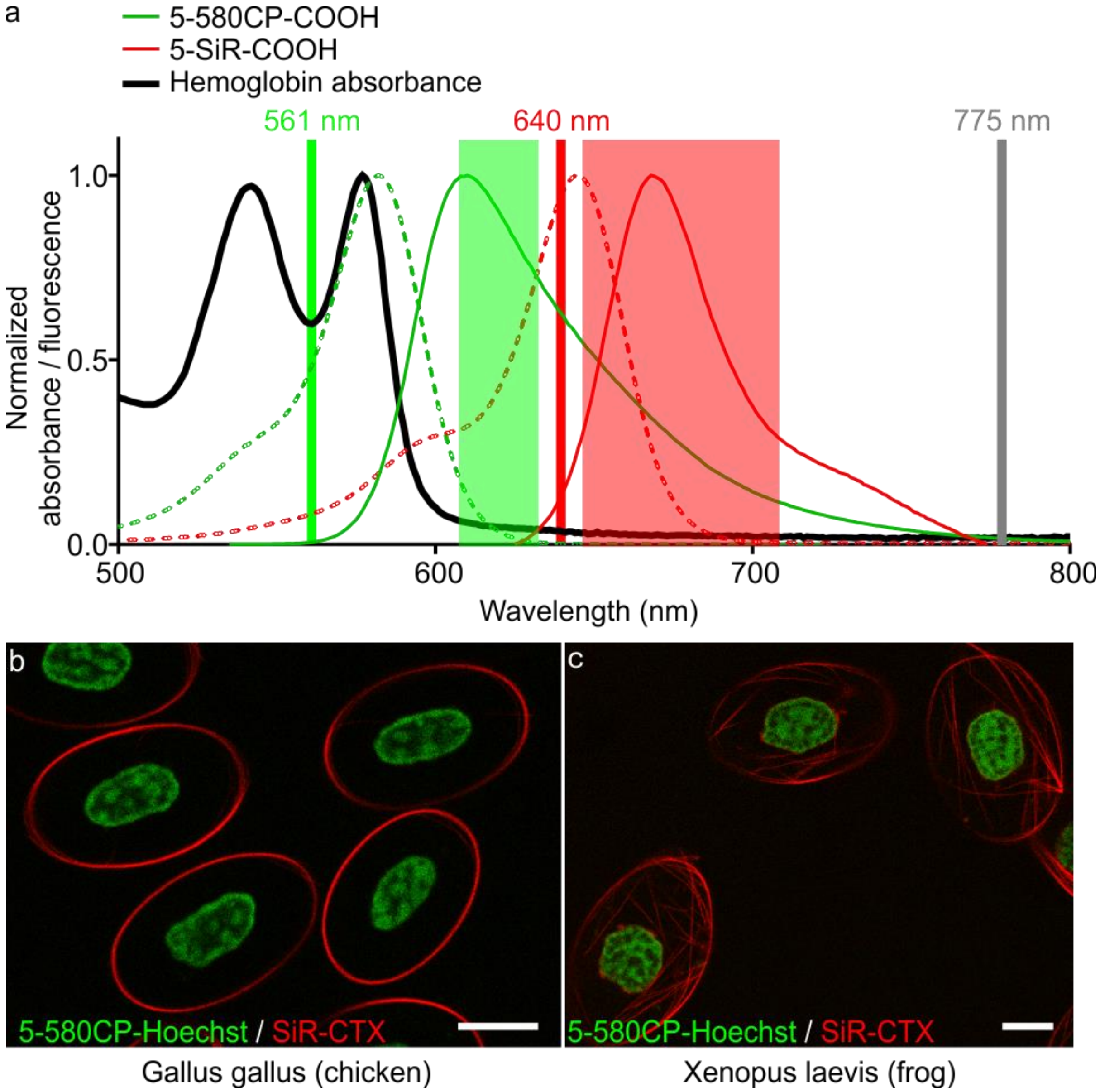

Figure S13. Two-color STED nanoscopy of avian and frog erythrocytes. (a) Two color microscopy setup used for imaging of 580CP and SiR probes. Note that 561 nm excitation laser is located in the haemoglobin absorbance minimum and 775 nm STED depletion laser is far from haemoglobing and fluorescent dye absorbance minimizing bleaching and reexcitation effects. (b) Two-colour STED image of chicken (Gallus gallus) erythrocytes labelled with 1 µM 5-580CP-Hoechst and 1 µM SiR-CTX (tubulin probe) at RT for 1 h in the RBC buffer. No washing step was applied. Scale bar 5 µm. (c) Two-colour STED image of frog (Xenopus laevis) erythrocytes labelled with 1 µM 5-580CP-Hoechst and 2 µM SiR-CTX (tubulin probe) at RT for 1 h in the RBC buffer. No washing step was applied. Scale bar 5 µm.

Supplementary Movies

Video S1. Timelapse movie of the nucleus in living U-2 OS cell. Cells were stained with 1 µM 5-610CP-Hoechst. Aquired movie deconvolved using SVI Huygens. Scale bar 5 µm.

Video S2. Rotating maximum intesity projection of the intact Xenopus laevis erythocyte. The sample of whole blood was stained with 1µM SiR-CTX (red, tubulin) and 1 µM 5-580CP-Hoechst (green, nucleus) at RT for 1 h in the RBC buffer. No washing step was applied. Aquired z-stack was deconvolved using SVI Huygens.

Supplementary Tables

Table S1. Photophysical properties of rhodamine fluorescent dyes used in the study.

Dye	Solvent	λ_{max}^{abs} (nm)	λ_{max}^{em} (nm)	ε (m^{-1} cm^{-1})[a]	QY[a]	τ (ns)[a]
**5-TMR-COOH**	PBS	550	579	83000 ± 1600	0.411 ± 0.005	2.14 ± 0.02
	PBS + 0.1%SDS	552	580	83600 ± 1100	0.51 ± 0.02	2.80 ± 0.02
**6-TMR-COOH**	PBS	550	576	83300 ± 4200	0.359 ± 0.003	1.97 ± 0.02
	PBS + 0.1%SDS	550	576	86600 ± 4800	0.399 ± 0.002	2.05± 0.02
**5-580CP-COOH**	PBS	582	610	100400 ± 6100	0.563 ± 0.004	3.47± 0.01
	PBS + 0.1%SDS	586	613	104600 ± 5400	0.650 ± 0.003	4.07 ± 0.01
**6-580CP-COOH**	PBS	582	608	100300 ± 2500	0.584 ± 0.008	3.64 ± 0.03
	PBS + 0.1%SDS	583	609	100200 ± 4400	0.614 ± 0.006	3.63 ± 0.66
**5-610CP-COOH**	PBS	608	636	98200 ± 4000	0.471 ± 0.003	2.99 ± 0.01
	PBS + 0.1%SDS	608	637	106000 ± 5500	0.48 ± 0.01	2.99 ± 0.02
**6-610CP-COOH**	PBS	608	635	102000 ± 2500	0.483 ± 0.007	2.97 ± 0.01
	PBS + 0.1%SDS	609	636	101000 ± 2300	0.541 ± 0.005	3.11 ± 0.02
**5-GeR-COOH**	PBS	634	656	99200 ± 2800	0.414 ± 0.002	2.46 ± 0.01
	PBS + 0.1%SDS	638	660	96000 ± 3900	0.558 ± 0.007	3.46 ± 0.02
**6-GeR-COOH**	PBS	634	656	111000 ± 1900	0.427 ± 0.004	2.57 ± 0.01
	PBS + 0.1%SDS	635	657	101800 ± 600	0.47 ± 0.01	2.71 ± 0.01
**5-SiR-COOH**	PBS	645	670	97000 ± 1900	0.399 ± 0.005	2.61 ± 0.01
	PBS + 0.1%SDS	649	672	101000 ± 2000	0.595 ± 0.005	3.55 ± 0.01
**6-SiR-COOH**	PBS	645	668	99600 ± 5000	0.406 ± 0.007	2.69 ± 0.01
	PBS + 0.1%SDS	646	668	90000 ± 4000	0.459 ± 0.003	2.82 ± 0.01

Note: [a] Data presented as mean value with standart deviation, N = 3.

Table S2. Photophysical properties of DNA probes.

Probe name	Free Probe				Probe + hpDNA[a]					Probe + 0.1% SDS				
	λ_{max}^{Abs} (nm)	λ_{max}^{em} (nm)	ε ($M^{-1}cm^{-1}$)	QY	λ_{max}^{Abs} (nm)	λ_{max}^{em} (nm)	ε ($M^{-1}cm^{-1}$)	QY	τ (ns)	λ_{max}^{Abs}, (nm)	λ_{max}^{em} (nm)	ε[b] ($M^{-1}cm^{-1}$)	QY	τ (ns)
**Hoechst 33258**	339	508	31800 ± 1600	0.018 ± 0.001	350	455	36000 ± 1600	0.823 ± 0.004	2.80	349	484	42000 ± 400	0.497 ± 0.006	2.15 (51%)<br>4.39 (49%)
**Hoechst 33342**	340	510	35700 ± 1200	0.026 ± 0.001	352	455	40270 ± 6760	0.805 ± 0.001	2.79	349	489	42300 ± 2700	0.541± 0.002	2.59 (42%)<br>4.46 (58%)
**5-TMR-Hoechst**	562	587	20700 ± 8260	0.007 ± 0.002	558	586	54200 ± 7800	0.209 ± 0.001	2.68	556	585	83600 ± 1100	0.505 ± 0.001	3.55
**6-TMR-Hoechst**	566	578	26700 ± 6070	0.005 ± 0.002	562	585	62100 ± 3180	0.052 ± 0.001	0.6 (79%)<br>2.78 (21%)	555	581	86600 ± 4800	0.52 ± 0.002	3.49
**5-580CP-Hoechst**	593	618	39100 ± 8300	0.027 ± 0.009	588	613	84200 ± 4400	0.372 ± 0.002	3.29	589	614	104600 ± 5400	0.583 ± 0.002	4.18
**6-CP580-Hoechst**	605	633	34400 ± 9700	0.011 ± 0.004	594	619	77600 ± 6300	0.124 ± 0.001	0.93 (65%)<br>3.22 (35%)	590	616	100200 ± 4400	0.598 ± 0.002	4.27
**5-610CP-Hoechst**	619	643	35000 ± 9800	0.052 ± 0.004	614	641	84300 ± 7400	0.432 ± 0.001	3.45	614	641	106000 ± 5500	0.576 ± 0.004	4.22
**6-610CP-Hoechst**	626	654	38500 ± 4600	0.033 ± 0.003	618	644	80300 ± 2600	0.282 ± 0.005	1.78 (50%)<br>3.05 (50%)	615	642	101000 ± 2300	0.614 ± 0.002	4.32
**5-GeR-Hoechst**	654	664	12100 ± 3150	0.054 ± 0.019	641	660	26600 ± 2900	0.392 ± 0.008	2.87	640	661	96000 ± 3900	0.591 ± 0.003	3.83
**6-GeR-Hoechst**	654	659	15400 ± 480	0.033 ± 0.006	643	662	52400 ± 3700	0.207 ± 0.002	0.98 (31%)<br>2.63 (69%)	642	663	101800 ± 600	0.538 ± 0.003	3.76
**5-SiR-Hoechst**	659	671	13000 ± 300	0.007 ± 0.001	651	672	38000 ± 3400	0.374 ± 0.006	2.99	651	673	101000 ± 2000	0.538 ± 0.004	3.93
**6-SiR-Hoechst**	667	673	14500 ± 2540	0.003 ± 0.002	654	677	54600 ± 10300	0.156 ± 0.002	1.07 (53%)<br>2.72 (47%)	652	676	90000 ± 4000	0.524 ± 0.001	3.94

Note: [a] For the spectroscopy studies hairpin-forming oligonucleotide 5'-CGCGAATTCGCGTTTTCGCGAATTCGCG were used; probe concentration - 2µM, oligonucleotide concentration - 30 µM; probes were incubated for 2h at room temperature before the measurements. Data presented as mean value with standart deviation, N = 3. [b] Extinction coeficients of probes were equated to the maximal extinction coficients of fluorescent dyes

Table S3. Absorbance and fluorescence changes of DNA probes upon interaction with DNA or SDS.

Probe name	$\varepsilon_{max}^{DNA}/\varepsilon_{max}^{SDS}$ [a]	$\varepsilon_{max}^{DNA}/\varepsilon_{max}^{PBS}$ [a]	QY_{DNA}/QY_{PBS} [a]
**Hoechst 33342**	0.95 ± 0.22	1.1 ± 0.2	30 ± 1
**5-TMR-Hoechst**	0.65 ± 0.10	2.6 ± 1.4	30 ± 9
**6-TMR-Hoechst**	0.72 ± 0.08	2.3 ± 0.7	10 ± 4
**5-580CP-Hoechst**	0.81 ± 0.08	2.2 ± 0.6	14 ± 5
**6-CP580-Hoechst**	0.77 ± 0.10	2.3 ± 0.8	11 ± 4
**5-610CP-Hoechst**	0.78 ± 0.11	2.4 ± 0.9	8 ± 1
**6-610CP-Hoechst**	0.80 ± 0.04	2.1 ± 0.3	9 ± 1
**5-GeR-Hoechst**	0.28 ± 0.04	2.2 ± 0.8	7 ± 3
**6-GeR-Hoechst**	0.52 ± 0.04	3.4 ± 0.4	6 ± 1
**5-SiR-Hoechst**	0.38 ± 0.03	2.9 ± 0.3	53 ± 9
**6-SiR-Hoechst**	0.61 ± 0.12	3.8 ± 1.4	52 ± 35

Note: [a] Data presented as mean value with standart deviation, N = 3.

Table S4. Cytotoxicity of Dye-Hoechst DNA probes

Probe name	Cytotoxicity threshold (μM)
**Hoechst 33342**	0.4
**5-TMR-Hoechst**	0.063
**6-TMR-Hoechst**	>10
**5-580CP-Hoechst**	2.5
**6-580CP-Hoechst**	<1.25
**5-610CP-Hoechst**	2.5
**6-610CP-Hoechst**	<1.25
**5-GeR-Hoechst**	20
**6-GeR-Hoechst**	20
**5-SiR-Hoechst**	>40
**6-SiR-Hoechst**	>40

Table S5. Heterochromatin exclusion zone diameter near the nuclear pore complexes.

Probe name	Fibroblasts (nm)		U-2 OS (nm)	Hela (nm)	Chicken erythrocytes (nm)
	Living	Fixed			
**5-580CP-Hoechst**	164 ± 38	-	-	-	-
**5-610CP-Hoechst**	165 ± 12	160 ± 12	168 ± 10	151 ± 13	-
**5-GeR-Hoechst**	169 ± 12	-	-	-	-
**5-SiR-Hoechst**	139 ± 24	-	148 ± 11	144 ± 23	166 ± 8

Note: Data presented as mean value with standard deviation. See “Processing and visualization of acquired images” section for details about the estimation of the exclusion zome diameter.

Computation, molecular biology and biochemical methods

Estimation of the probe aggregation

The estimation of aggregation extent was performed using a centrifugation-based assay. The 2 µM solutions of probes were prepared by adding 1mM DMSO stock solution to PBS (Lonza,Cat. no. BE17-516F). After 1 h incubation at RT, the solutions were centrifuged for 1h at 20 ^{o}C in a centrifuge set at 16000g. The pellet and solution fractions were separated, the pellet was suspended in PBS + 0.1% SDS and the supernatant was supplemented with SDS to 0.1% concentration. After 1h incubation at RT, the pellet was fully dissolved, and fluorescence was measured for both supernatant and pellet fractions with a multiwell plate reader Spark® 20M (Tecan) in a glass bottom 96-well plate (MatTek, Cat. no. PBK96G-1.5-5-F) at room temperature. The previously described settings were used for excitation and emission. Aggregation percentage was calculated using the following formula S1:

$$Aggregation\ (\%) = \frac{F_{\mathrm{pellet}}}{F_{\mathrm{pellet}} + F_{\mathrm{solution}}} \times 100\% \quad \text{(S1)}$$

Preparation of hairpin DNA

For the DNA binding studies hairpin forming oligonucleotide 5'-CGCGAATTCGCGTTTTCGCGAATTCGCG-3' (28 bp) was purchased from Sigma-Aldrich. Previously, this hairpin DNA has been used for structural studies of the interaction of Hoechst 33342 with DNA[2]. Synthetic oligonucleotides were dissolved in PBS (Lonza, pH 7.4) at 1 mM concentration. Hairpin formed by putting the tube with hpDNA solution into boiling water bath which was slowly cooled down to room temperature.

Plasmid construction

pEBTet GW_Halo plasmid was constructed from pEBTet GW_SNAP plasmid which was characterized previously[3, 4]. Plasmids contain cytomegalovirus-type 2 tetracycline operator (tetO2)-tetO2 promoter which is "switched on" by addition of doxycycline. Halo-tag gene was amplified by PCR using pH6HTN His6HaloTag® T7 (Promega, Cat. No. G8031) as template (introducing R.NheI and R.XhoI sites), digested with R.NheI and R.XhoI and ligated into pEBTet GW_SNAP vector precut with the same restriction endonucleases. TelR15 coding gene was PCR amplified from pT7CFE1-TelR15-mTagBFP2 template (Addgene, Cat. no. 49643), and cloned into pEBTet GW_Halo vector by single step BP/LR recombination. Final construct sequence has been verified by seqencing.

Maintenance and preparation of cells

HeLa and U-2 OS cells were purchased from American Type Culture Collection (ATCC). Adult human dermal fibroblasts were puchased from Lonza. All cells were cultured in high-glucose DMEM (Life Technologies, Cat. No. 31053-028) supplemented with GlutaMAX-1 (Life

Technologies, Cat. No. 35050-038) and 10% fetal bovine serum (FBS, Life Technologies, Cat. No. 10270-106) in a humidified 5% CO_2 incubator at 37 °C. The cells were split every 3-4 days or at confluence. Cells were seeded in glass bottom 12-well plates (MatTek Corporation, Cat. No. P12G-1.0-14-F).

Cells were stained with the probes in DMEM (Thermo Fisher Scientific, Cat. No. 31053-028) supplemented with 10% FBS (Thermo Fisher Scientific, Cat. No. 10082139) at 37 °C and 5% CO_2. Afterwards, the cells were washed 2 times with HBSS (Hanks' balanced salt solution, Lonza, Cat. No. BE10-527F). Imaging was performed in DMEM with 10% FBS.

Inducible U-2 OS cell line was generated by transiently transfecting cells with pEBTet TelR15-Halo expression vector at ~70% confluence using Lipofectamine 2000 (Thermo Fisher Scientific, Cat. No. 11668027) following manufacturer's recomendations. Transfected cells were cultivated in DMEM (Thermo Fisher Scientific, Cat. No. 31053-028) supplemented with 10% FBS (Thermo Fisher Scientific, Cat. No. 10082139) and 1 µg/ml puromycin (Sigma, Cat. No. P9620) for 2 weeks untill growth rate of the cells reached similar rate to untransfected cells in media without antibiotics. Thereafter, selected cells were frozen in 10% DMSO and stored at -80°C. Expression of transgene was induced using 0.1 µg/ml doxycycline (Sigma, Cat. no. D9891) for 48 - 72 h before imaging experiment.

Preparation of erythrocytes

Sodium citrate treated whole blood from frog or chicken was diluted 1:500 with RBC buffer (10 mM HEPES pH 7.4, 150 mM NaCl, 0.1% glucose) in eppendorf tubes. Probes from a 1mM DMSO stock were added to the final concentration of 1 µM and incubated for 60 min at room temperature. The suspension was transferred to a poly-lysine coated 35 mm glass bottom dish (MatTek Corporation, cat. P35GC-1.5-10-C) and imaged directly without washing.

Cell cycle analysis by imaging flow cytometry

HeLa cells were grown in 6-well plates (250.000 cells per well) for 24 h in the presence of the fluorescent probe in variable concentrations. The probes were dissolved in DMSO to concentration corresponding to 1000 - 250x of final concentration in the growth media, the control samples were prepared by accordingly adding 0.25 - 0.1% DMSO. We found that HeLa cells do not adhere strongly to the plastic bottom of the 6-well plate and thus the trypsination step could be omitted. The cells were simply washed off and suspended in 1 ml of the growth medium by intensively pipetting up and down. Next, the cells were processed according to the NucleoCounter® NC-3000™ two-step cell cycle analysis protocol. In particular, ~500,000 cells were harvested by centrifuging at room temperature for 5 min at 400*g*. Afterwards, the cells were resuspended in 250 µl lysis solution (Solution 10, Chemometec Cat. No. 910-3010) supplemented with 10 µg/ml DAPI (Solution 12, Chemometec Cat. No. 910-3012), incubated at 37 °C for 5 min. Then 250 µl of stabilization solution (Solution 11, Chemometec Cat. No.

910-3011) was added. Cells were counted on a NucleoCounter® NC-3000™ in NC-Slide A2™ slides (Chemometec, Cat. No. 942-0001) loaded with ~30 µl of each of the cell suspensions into the chambers of the slide. Each time, ~10,000 cells in total were measured, and the obtained cell cycle histograms were analysed with ChemoMetec NucleoView NC-3000 software, version 2.1.25.8. All experiments were repeated three times and the results are presented as means with standard deviations. The obtained mean values were compared by running multiple t-tests on GraphPad Prism version 6.0 software.

Processing and visualization of acquired images

All acquired or reconstructed images were processed and visualized using Fiji[5]. Line profiles were measured using the "straight line" tool with the line width set to 3 pixels.

For the signal measurements, image files were converted to TIF file using Fiji and analyzed with CellProfiler 3.1.5 (ref.[6]), where the pipeline identified the nuclear region and measured the mean signal in this region. Background signal was measured in the region which is 3 pixels (450 nm) away from the nulear border and 7 pixels (1050 nm) wide. The background substracted signal was processed with GraphPad Prism 6.

The estimation of the diameter of heterochromatin exclusion zones near nuclear pore complexes was performed with custom written Matlab (Mathworks, Natick, US) routines:

Correction of drift during image acquisition

Frame to frame pixel shifts were estimated by computing all possible 2D cross-correlations between frames of the 3D data stacks that were not more than 450nm apart (well within the FWHM of the PSF along z). The pixel shift was taken as the position of the maximum amplitude of a 2D cross-correlation relative to the origin of the coordinate system.

Then a smooth drift curve was estimated, independently for x and y directions, that realizes the estimated pixel shifts as closely as possible. The minimization metric was a weighted least square distance between drift and estimated pixel shifts. An additional regularization term proportional to the total variation of the drift was introduced that additionally smoothed the drift curve. The drift in the data was then corrected by shifting data frames by whole pixels in the opposite direction of the estimated drift.

Deconvolution of 3D image z-stacks

Drift corrected image stacks were deconvolved with a measured point spread function (PSF) using the Richardson-Lucy algorithm[7] for 10-20 iterations on each measured 3D data set. The Richardson-Lucy method is a nonlinear image restoration approach taking into account the non-negativity of the sample.

The measured PSF consisted of the average 3D image of about 50 small beads (40 nm diameter, filled with fluorescent dye) distributed sparsely in a layer, which were excited at 640 nm and 60 mW STED light power was applied. The averaged bead image exhibited a lateral

FWHM of about 80 nm and an axial FWHM of about 650 nm and showed the characteristic tail distribution outside the focal plane resulting from inefficient STED suppression far away from the focal point. Using such a PSF allows to remove out-of-focus background in the recorded images during the deconvolution. In general, it can be expected that the widths of the true PSFs deviate somewhat from this measured PSF, depending on the dye in use (in particular its cross-section for stimulated emission) and the applied STED light power.

Estimation of nuclear pore exclusion zone diameters

The nuclear pore exclusion zones were visible as characteristic dark, symmetric holes in the image frames near the bottom and upper side of the nucleus. The visibility of these zones was enhanced quite significantly in the deconvolved images, while the raw data was often dominated by noise.

A certain number of frames (2-3 corresponding to an axial extent of 300-450nm), usually at the bottom side of the nucleus, of the deconvolved image stack were added up. In the resulting image, isolated, well visible zones were selected manually, between 15 and 100 per cell. The modulation of the depth of the hole was typically about 20-30% of the surrounding image intensity. The intensity distribution surrounding an exclusion zone was typically very inhomogeneous. Therefore, patches of 400 x 400 nm^2 area size were taken centered on the manually selected positions and the data of all cells of the same imaging condition was pooled. Then randomly 15 patches were selected repeatedly, averaged to deliver a more symmetric hole with a flat surrounding and a symmetric 2D Gaussian peak was fitted to it using least squares minimization. The estimated FWHM was recorded and the procedure was repeated multiple times. If n patches were available for a particular condition 20n/log(n) times 15 patches were drawn randomly and the average image of these patches was fitted.

General experimental information and synthesis

NMR spectra were recorded at 25 °C with an Agilent 400-MR spectrometer at 400.06 MHz (^{1}H) and 100.60 MHz (^{13}C), Bruker Avance III HD 500 spectrometer (av500) at 500.25 MHz (^{1}H) and 125.80 MHz (^{13}C), Varian Mercury Plus 300 spectrometer at 300.14 MHz (^{1}H), Varian INOVA 600 (I600) spectrometer at 599.74 MHz (^{1}H) and are reported in ppm. All ^{1}H and ^{13}C spectra are referenced to tetramethylsilane (δ = 0 ppm) using the residual signals of the solvents according to the values reported in literature (ref [8]). Multiplicities of signals are described as follows: s = singlet, d = doublet, t = triplet, q = quartet, p = pentet, m = multiplet or overlap of non-equivalent resonances; br = broad signal. Coupling constants (*J*) are given in Hz. ESI-MS were recorded on a Varian 500-MS spectrometer (Agilent). ESI-HRMS were recorded on a MICROTOF spectrometer (Bruker) equipped with ESI ion source (Apollo) and direct injector with LC autosampler Agilent RR 1200. Liquid chromatography: Analytical HPLC was performed on a Knauer Azura liquid chromatography system with a binary P 6.1L pump (Article No. EPH35, Knauer), UV diode array detector DAD 6.1L (Article No. ADC11, Knauer), an injection valve with a 20 µL loop and two electrical switching valves V 2.1S with 6-port multiposition valve head (Article No. EWA10, Knauer). Analytical columns: Knauer Eurospher II 100-5 C18, 5 µm, 150×4 mm (Article No. 15DE181E2J, Knauer) or Interchim Uptisphere Strategy C18-HQ, 10 µm, 250×4.6 mm (Article No. US10C18HQ-250/P46, Interchim), typical flow rate: 1.2 mL/min, unless stated otherwise. Preparative HPLC was performed on an Interchim puriFlash 4250 2X preparative HPLC/Flash hybrid system (Article No. 1I5140, Interchim) with a 2 mL / 5 mL injection loop, a 200-600 nm UV-Vis detector and an integrated ELSD detector (Article No. 1A3640, Interchim). Preparative column: Eurospher II 100-5 C18 5 µm, 250×20.0 mm (Article No.: 25PE181E2J, Knauer), typical flow rate: 25 mL/min, unless specified otherwise. Analytical TLC was performed on Merck Millipore ready-to-use plates with silica gel 60 (F254) (Cat. No. 1.05554.0001). Flash chromatography was performed on Biotage Isolera flash purification system using the type of cartridge and solvent gradient indicated.

2,2'-(4-bromo-1,3-phenylene)bis(4,4-dimethyl-4,5-dihydro-1,3-oxazole) (SI-1)

4-Bromoisophthalic acid (3.0 g, 12.24 mmol) was suspended in $SOCl_2$ (15 mL), 3 drops of DMF were added and the mixture was refluxed for 3 h. The solution was evaporated to dryness and the residue was dissolved in CH_2Cl_2. The resulting solution of acyl chloride was added dropwise to the mixture of 2-amino-2-methyl-1-propanol (3.27 g, 36.72 mmol, 3 equiv) and DIPEA (6.4 mL, 36.72 mmol, 3 equiv) in CH_2Cl_2 (50 mL), cooled in ice-water bath. The resulting suspension was stirred overnight at r.t., quenched by addition of sat. aq. $NaHCO_3$ (50 mL), extracted with EtOAc (3×50 mL), the combined organic layers were washed with water, brine and dried over Na_2SO_4. After filtration, the solvent was evaporated to give the

crude solid diamide, which was used directly in the next step. The solid was suspended in CH_2Cl_2 (100 mL) and 1,8-diazabicyclo[5.4.0]undec-7-ene (DBU; 5.47 mL, 36.72 mmol, 3 equiv) was added to the suspension, turning it into a clear solution. The solution was cooled in ice-water bath, followed by addition of perfluoro-1-butanesulfonyl fluoride (nonaflyl fluoride, NfF; 5.27 mL, 4.44 g, 14.69 mmol, 2.4 equiv) dropwise over 5 min. The resulting solution was stirred at r.t. for 3 h, quenched with sat. aq. $NaHCO_3$ (50 mL), extracted with CH_2Cl_2 (3×50 mL), the organic extracts were washed with brine and dried over Na_2SO_4. The product was isolated by flash column chromatography (Teledyne Isco RediSep Rf 120g, gradient 20% to 80% EtOAc – CH_2Cl_2), fractions containing the product were evaporated to give 2.88 g of viscous slightly yellow oil (yield 67% over 2 steps).

^{1}H NMR (400 MHz, $CDCl_3$) δ 8.19 (d, *J* = 2.2 Hz, 1H), 7.79 (dd, *J* = 8.4, 2.2 Hz, 1H), 7.64 (d, *J* = 8.4 Hz, 1H), 4.11 (s, 2H), 4.09 (s, 2H), 1.39 (s, 6H), 1.35 (s, 6H).

^{13}C NMR (101 MHz, $CDCl_3$) δ 161.0, 160.8, 133.8, 131.0, 130.9, 130.7, 127.4, 125.1, 79.6, 79.4, 68.5, 68.0, 28.5, 28.4.

ESI-MS, positive mode: m/z = 351.1 $[M+H]^+$.

HRMS (ESI) calcd for $C_{16}H_{20}N_2O_2Br$ $[M+H]^+$ 353.0683, found 353.0683.

5-SiR-COOH

To a solution of bis-oxazoline **SI-1** (606 mg, 1.73 mmol, 2 equiv) in THF (15 mL), cooled to -78°C, *t*-BuLi (2.04 mL of 1.7 M in pentane, 3.46 mmol, 4 equiv) was added, and the mixture was stirred at -78°C for 1 h. A solution of ketone **SI-2** [9] (280 mg, 0.863 mmol) in THF (20 mL) was added dropwise, and the mixture was allowed to warm up to r.t. and stirred for 3 h. Acetic acid (2 mL) was added in to the brownish reaction mixture which immediately turned dark blue. Mixture was evaporated on a rotary evaporator to a viscous residue, which was then dissolved in 6 N HCl (40 mL), and the resulting orange-brown solution was stirred at 80°C overnight. The yellow mixture was cooled and pH was adjusted to 1-2 by careful addition of $NaHCO_3$. The mixture was extracted with CH_2Cl_2 (5×50 mL), the poorly soluble dye was dissolved by addition of methanol to the combined organic layers, which were then dried over Na_2SO_4, filtered and evaporated. The product was isolated by flash column chromatography (Büchi Reveleris HP silica 40 g, gradient 20% to 100% hexane – EtOAc with constant 1% v/v AcOH adittive), the fractions containing the product were evaporated, the residue was redissolved in 1,4-dioxane – water (1:1), microfiltered through a 0.45 µm PTFE membrane filter and lyophilized. Yield 175 mg (43%), blue solid.

^{1}H NMR (400 MHz, CD_3CN) δ 8.47 (d, *J* = 1.4 Hz, 1H), 8.27 (dd, *J* = 8.0, 1.4 Hz, 1H), 7.28 (d, *J* = 8.0 Hz, 1H), 7.05 (d, *J* = 2.9 Hz, 2H), 6.74 (d, *J* = 9.0 Hz, 2H), 6.63 (dd, *J* = 9.0, 2.9 Hz, 2H), 2.95 (s, 12H), 0.65 (s, 3H), 0.55 (s, 3H).

^{13}C NMR (101 MHz, CD_3CN) δ 170.5, 166.6, 160.3, 150.7, 137.1, 136.3, 132.4, 131.3, 129.0, 127.8, 127.4, 125.3, 117.5, 114.7, 40.4, 0.1, -1.0.
HRMS (ESI) calcd for $C_{27}H_{29}N_2O_4Si$ $[M+H]^+$ 473.1891, found 473.1883.

5-610CP-COOH

To a solution of bis-oxazoline **SI-1** (233 mg, 0.65 mmol, 2 equiv) in THF (5 mL), cooled to -78°C, t-BuLi (0.8 mL of 1.7 M in pentane, 1.36 mmol, 4 equiv) was added, and the mixture was stirred at -78°C for 1 h, gradually turning light orange. A solution of ketone **SI-3** (ref.[10]) (101 mg, 0.33 mmol) in THF (5 mL) was added dropwise, and the mixture was allowed to warm up to r.t. and stirred for 2 h. The resulting brownish solution was cooled in ice-water bath, and acetic acid (1 mL) was added. The dark blue reaction mixture was evaporated on a rotary evaporator to a viscous residue, which was dissolved in 6 N HCl (20 mL), and the resulting dark orange solution was stirred at 80°C overnight. The yellow mixture was cooled and pH was adjusted to 1-2 by careful addition of $NaHCO_3$. The mixture was extracted with CH_2Cl_2 (5×50 mL), the poorly soluble dye was dissolved by addition of methanol to the combined organic layers, which were then dried over Na_2SO_4, filtered and evaporated. The product was isolated by flash column chromatography (Büchi Reveleris HP silica 24 g, gradient 40% to 100% hexane – EtOAc with constant 1% v/v AcOH adittive), the fractions containing the product were evaporated, the residue was redissolved in 1,4-dioxane – water (1:1), microfiltered through a 0.45 µm PTFE membrane filter and lyophilized. Yield 105 mg (68%), dark blue solid.
^1H NMR (400 MHz, CD_3OD) δ 8.62 (d, *J* = 1.6 Hz, 1H), 8.25 (dd, *J* = 8.0, 1.6 Hz, 1H), 7.03 (d, *J* = 2.6 Hz, 2H), 7.15 (d, *J* = 8.0 Hz, 1H), 7.03 (d, *J* = 2.6 Hz, 2H), 6.70 (d, *J* = 9.0 Hz, 2H), 6.63 (dd, *J* = 9.0, 2.6 Hz, 2H), 3.07 (s, 12H), 1.84 (s, 3H), 1.73 (s, 3H).
^{13}C NMR (101 MHz, CD_3OD) δ 171.2, 169.2, 154.5, 153.8, 151.8, 135.4, 135.0, 132.7, 131.3, 129.1, 127.4, 120.6, 113.3, 111.0, 40.9, 40.7, 35.7, 33.1
ESI-MS, positive mode: m/z = 457.2 $[M+H]^+$.
HRMS (ESI) calcd for $C_{28}H_{29}N_2O_4$ $[M+H]^+$ 457.2122, found 457.2123.

Di-*tert*-butyl 4-bromoisophtalate (SI-4)

In a 100 mL round-bottom flask, equipped with a reflux condenser, 4-bromoisophthalic acid (2 g, 8.16 mmol) and 4-(dimethylamino)pyridine (DMAP; 199 mg, 1.63 mmol, 0.2 equiv) were mixed with DMF (2 mL). Di-tert-butyl dicarbonate (Boc_2O; 5.34 g, 24.48 mmol, 3 equiv) was dissolved in toluene (20 mL) and added in one portion. The resulting suspension was stirred at 80 °C (bath temperature) for 30 min. TLC (10% ethyl acetate – hexane) showed incomplete conversion, so another portion of Boc_2O (1.78 g, 8.16 mmol, 1 equiv) in toluene (7 mL) was added, and the heating resumed for further 30 min. A cloudy solution eventually formed. Upon cooling to room temperature, sat. aq. $NaHCO_3$ (40 mL) was added, the mixture was extracted

with ethyl acetate – hexane (2:1, 3×30 mL), the combined organic layers were washed with water (2×30 mL), brine and dried over Na_2SO_4. The product was isolated by flash column chromatography (Teledyne Isco RediSep Rf 40g, gradient 5% to 60% Hexane – [hexane:EtOAc 10%]). The fractions containing the product were evaporated and dried on a rotary evaporator at 60°C to ensure low residual Boc_2O content. Yield 1.83 g (63%) of viscous colourless oil.

^{1}H NMR (400 MHz, $CDCl_3$) δ 8.23 (d, *J* = 2.2 Hz, 1H), 7.84 (dd, *J* = 8.4, 2.2 Hz, 1H), 7.65 (d, *J* = 8.4 Hz, 1H), 1.60 (s, 9H), 1.58 (s, 9H).

^{13}C NMR (101 MHz, $CDCl_3$) δ 165.1, 164.3, 134.4, 134.0, 132.2, 131.5, 131.1, 125.6, 83.0, 81.9, 28.1.

***Tert*-butyl 3,6-bis{[*tert*-butyl(dimethyl)silyl]oxy}-10,10-dimethyl-3'-oxo-3'*H*,10*H*-spiro[anthracene-9,1'-[2]benzofuran]-5'-carboxylate (SI-6)**

In a 25 mL round-bottom flask, a degassed solution of di-*tert*-butyl 4-bromoisophtalate (**SI-4**) (740 mg, 2.07 mmol, 2 eq.) in anhydrous THF (6 mL) and pentane (4mL) was cooled to -100 °C (bath temperature, diethyl ether – liquid N_2). n-Butyllithium (1.3 mL of 1.6 M solution in hexanes, 2.07 mmol, 2 eq.) was carefully introduced through a needle. Clear solution quickly turned orange and then deep brown; it was stirred at -100 °C for 10 min, and the solution of ketone **SI-5** [11] (500 mg, 1.04 mmol 1 eq.) in THF (3 mL) was injected along the wall of the flask. The flask was then placed into a -78°C bath (dry ice – acetone) and stirred for 10 min. The cooling bath was removed, the mixture was allowed to warm up to room temperature and stirred for further 30 min. The reaction mixture was quenched with water (10 mL), adjusted to pH ~ 5 with acetic acid, extracted with ethyl acetate (3×30 mL), the combined organic layers were washed with brine and dried over Na_2SO_4. The product was isolated by flash column chromatography (Büchi Reveleris HP silica; gradient 0% to 20% ethyl acetate – hexane) as white solid, yield 292 mg (41%).

^{1}H NMR (600 MHz, $CDCl_3$) δ 8.58 (dd, *J* = 1.6, 0.8 Hz, 1H), 8.22 (dd, *J* = 8.0, 1.6 Hz, 1H), 7.06 (dd, *J* = 8.0, 0.8 Hz, 1H), 7.04 – 7.06 (m, 2H), 6.56 – 6.57 (m, 4H), 1.78 (s, 3H), 1.69 (s, 3H), 1.61 (s, 9H), 0.96 (s, 19H), 0.19 (s, 12H).

^{13}C NMR (126 MHz, $CDCl_3$) δ 169.6, 164.1, 158.6, 156.3, 146.8, 135.6, 133.4, 129.1, 126.7, 126.4, 123.8, 123.7, 118.9, 117.5, 86.7, 82.1, 38.2, 35.1, 32.7, 28.3, 25.8, 18.3, -4.2.

ESI-MS, positive mode: m/z = 687.4 $[M+H]^+$.

HRMS (ESI) calcd for $C_{40}H_{55}O_6Si_2$ $[M+H]^+$ 687.3532, found 687.3522.

***Tert*-butyl 3,6-dihydroxy-10,10-dimethyl-3'-oxo-3'*H*,10*H*-spiro[anthracene-9,1'-[2]benzofuran]-5'-carboxylate (SI-7)**

To a cooled (ice-water bath) solution of **SI-6** (292 mg, 0.41 mmol) in THF (15 mL) tetrabutylammonium fluoride trihydrate (517 mg, 1.64 mmol, 4 equiv) solution in THF (5 mL) was added. The resulting intense red solution was stirred at 0 °C for 1 h. Sat. aq. NH_4Cl (20

mL) was added followed by minimal amount of water necessary to dissolve the solids, the mixture was extracted with ethyl acetate (3×30 mL), the combined organic layers were washed with brine and dried over Na_2SO_4. The product was isolated by flash column chromatography (Teledyne Isco RediSep Rf 24 g; gradient 0% to 30% ethyl acetate – CH_2Cl_2) and evaporated to give **SI-7** as viscous yellow oil. Yield 186 mg (99%).

^{1}H NMR (300 MHz, $CDCl_3$) δ 8.59 (dd, *J* = 1.5, 0.8 Hz, 1H), 8.23 (dd, *J* = 8.1, 1.5 Hz, 1H), 7.05 (dd, *J* = 8.1, 0.8 Hz, 1H), 7.01 (d, *J* = 2.4 Hz, 2H), 6.85 (s, 2H), 6.54 (dd, *J* = 8.6, 2.4 Hz, 2H), 6.44 (d, *J* = 8.6 Hz, 2H), 1.61 (s, 9H), 1.53 (s, 3H), 1.35 (s, 3H).

^{13}C NMR (126 MHz, $CDCl_3$) δ 170.9, 164.3, 158.6, 156.7, 147.4, 135.9, 133.5, 129.2, 126.62, 126.56, 123.9, 122.3, 114.8, 112.9, 82.6, 38.2, 34.8, 32.3, 28.2.

ESI-MS, positive mode: m/z = 459.2 $[M+H]^+$.

HRMS (ESI) calcd for $C_{28}H_{27}O_6$ $[M+H]^+$ 459.1802, found 459.1804.

***Tert*-butyl 10,10-dimethyl-3'-oxo-3,6-bis[(trifluoromethanesulfonyl)oxy]-3'*H*,10*H*-spiro[anthracene-9,1'-[2]benzofuran]-5'-carboxylate (SI-8)**

Trifluoromethanesulfonic anhydride 1M solution in DCM (1.62 mL, 1.62 mmol, 4 eq.) was added dropwise to a solution of **SI-7** (186 mg, 0.406 mmol) and pyridine (261 µL, 3.25 mmol, 8 eq.) in dry CH_2Cl_2 (10 mL), cooled in ice-water bath. The flask was then removed from the cooling bath, and the mixture was stirred at rt for 1 h. Afterwards, the mixture was diluted with water (30 mL), extracted with CH_2Cl_2 (3×20 mL), the combined extracts were washed with water, brine and dried over Na_2SO_4. The product was isolated by flash column chromatography (Teledyne Isco RediSep Rf 24 g; gradient 5% to 40% ethyl acetate – hexane) as white solid, yield 217 mg (74%).

^{1}H NMR (500 MHz, $CDCl_3$) δ 8.64 (dd, *J* = 1.5, 0.7 Hz, 1H), 8.29 (dd, *J* = 8.0, 1.5 Hz, 1H), 7.54 (d, *J* = 2.6 Hz, 2H), 7.03 – 7.11 (m, 3H), 6.86 (d, *J* = 8.9 Hz, 2H), 1.88 (s, 3H), 1.78 (s, 3H), 1.61 (s, 9H).

^{13}C NMR (126 MHz, $CDCl_3$) δ 168.7, 163.6, 157.0, 150.2, 147.0, 136.5, 134.6, 130.9, 130.1, 127.2, 126.0, 123.6, 120.4, 119.7, 83.9, 82.6, 38.9, 34.8, 32.7, 28.1.

ESI-MS, positive mode: m/z = 723.1 $[M+H]^+$.

HRMS (ESI) calcd for $C_{30}H_{25}F_6O_{10}S_2$ $[M+H]^+$ 723.0788, found 723.0784.

***Tert*-butyl 3,6-bis[(*tert*-butoxycarbonyl)(methyl)amino]-10,10-dimethyl-3'-oxo-3'*H*,10*H*-spiro[anthracene-9,1'-[2]benzofuran]-5'-carboxylate SI-9**

A mixture of **SI-8** (217 mg, 0.3 mmol), tert-butyl N-methylcarbamate (98 mg, 0.75 mmol, 2.5 eq.), $Pd_2(dba)_3$ (27 mg, 0.03 mmol, 10 mol%), Xantphos (52 mg, 0.09 mmol, 30 mol%) and Cs_2CO_3 (293 mg, 0.09 mmol, 3 eq.) in dry dioxane (2.5 mL) was degassed on a Schlenk line and stirred at 100 °C under argon (bath temperature) in a sealed flask for 18 h. Upon cooling, the resulting brown mixture was diluted with water (30 mL), extracted with ethyl acetate (3×30 mL), the combined organic layers were washed with brine and dried over Na_2SO_4. The filtrate

was evaporated and the product was isolated by flash column chromatography (Büchi Reveleris HP silica 24 g; gradient 5% to 60% hexane– EtOAc) as viscous yellowish oil, yield 154 mg (75%).

^{1}H NMR (400 MHz, $CDCl_3$) δ 8.61 (dd, *J* = 1.5, 0.8 Hz, 1H), 8.23 (dd, *J* = 8.0, 1.5 Hz, 1H),. 7.54 (d, *J* = 2.2 Hz, 2H), 7.07 (dd, *J* = 8.0, 0.8 Hz, 1H), 6.98 (dd, *J* = 8.6, 2.2 Hz, 2H), 6.67 (d, *J* = 8.6 Hz, 2H), 3.26 (s, 6H), 1.85 (s, 3H), 1.75 (s, 3H), 1.61 (s, 9H), 1.45 (s, 18H).

^{13}C NMR (101 MHz, $CDCl_3$) δ 169.5, 164.1, 158.2, 154.4, 145.4, 144.7, 135.8, 133.8, 128.0, 127.3, 126.7, 126.5, 123.8, 123.5, 123.6, 85.8, 82.3, 80.7, 38.3, 37.1, 35.0, 32.8, 28.4, 28.2.

ESI-MS, positive mode: m/z = 685.4 $[M+H]^+$.

HRMS (ESI) calcd for $C_{40}H_{49}N_2O_8$ $[M+H]^+$ 685.3483, found 685.3474.

5-580CP-COOH

Trifluoroacetic acid (1 mL) was added dropwise to a solution of **SI-9** (154 mg, 0.225 mmol) in CH_2Cl_2 (4 mL). The resulting bright red solution was stirred at room temperature overnight. The reaction mixture was then evaporated to dryness, the residue was re-evaporated three times with toluene to remove excess trifluoroacetic acid. The residue was lyophilized from aqueous dioxane. Product obtained as trifluoroacetic acid salt, yield 120 mg (98%), purple-blue solid.

^{1}H NMR (400 MHz, CD_3OD) δ 8.86 (d, *J* = 1.6 Hz, 1H)., 8.36 (dd, *J* = 8.0, 1.6 Hz, 1H), 7.44 (d, *J* = 8.0 Hz, 1H), 7.10 (d, *J* = 2.3 Hz, 2H), 6.94 (d, *J* = 9.2 Hz, 2H), 6.60 (dd, *J* = 9.2, 2.3 Hz, 2H), 3.03 (s, 6H), 1.81 (s, 3H), 1.70 (s, 3H).

^{13}C NMR (101 MHz, CD_3OD) δ 166.6, 165.9, 165.3, 157.9, 141.9, 136.9, 132.6, 132.0, 131.9, 131.5, 130.7, 128.6, 128.2, 120.3, 41.3, 34.0, 30.4, 28.6.

ESI-MS, positive mode: m/z = 429.2 $[M+H]^+$.

HRMS (ESI) calcd for $C_{26}H_{25}N_2O_4$ $[M+H]^+$ 429.1809, found 429.1809.

5-GeR-COOH

To a solution of bis-orthoester **SI-10** [12] (111 mg, 0.27 mmol, 2 equiv) in THF (10 mL), cooled to -78°C, *t*-BuLi (320 µL of 1.7 M in pentane, 0.54 mmol, 4 equiv) was added, and the mixture was stirred at -78°C for 1 h. A solution of ketone **SI-11** [13] (50 mg, 0.135 mmol) in THF (5 mL) was added dropwise, and the mixture was allowed to warm up to r.t. and stirred for 2 h. The resulting solution was cooled in ice-water bath, and acetic acid (1 mL) was added and stirred for 10 minutes. The dark blue reaction mixture was evaporated on a rotary evaporator to a viscous residue, which was dissolved in 6 N HCl (20 mL), and the resulting orange-brown solution was stirred at 80°C overnight. The yellow mixture was cooled and pH was adjusted to 1-2 by careful addition of $NaHCO_3$. The mixture was extracted with CH_2Cl_2 (5×50 mL), the poorly soluble dye was dissolved by addition of methanol to the combined organic layers, which were then dried over Na_2SO_4, filtered and evaporated. The product was isolated by flash

column chromatography (Büchi Reveleris HP silica 24 g, gradient 20% to 80% hexane – EtOAc with constant 1% v/v AcOH adittive), the fractions containing the product were evaporated, the residue was redissolved in acetonitrile – water (1:1), microfiltered through a 0.45 µm PTFE membrane filter and lyophilized. Yield 35 mg (68%), blue solid.

^{1}H NMR (400 MHz, CD_3OD) δ 8.52 (d, *J* = 1.5 Hz, 1H), 8.42 (dd, *J* = 8.0, 1.5 Hz, 1H), 7.55 (d, *J* = 8.0 Hz, 1H), 6.99 (d, *J* = 2.9 Hz, 2H), 6.72 (d, *J* = 8.9 Hz, 2H), 6.53 (dd, *J* = 8.9, 2.9 Hz, 2H), 2.94 (s, 12H), 0.74 (s, 3H), 0.70 (s, 3H).

^{13}C NMR (101 MHz, CD_3OD) δ 169.9, 166.8, 156.6, 149.9, 140.4, 134.3, 132.6, 130.1, 127.7, 127.6, 126.8, 125.7, 116.8, 112.1, 39.0, -1.0, -4.0.

ESI-MS, positive mode: m/z = 519.1 $[M+H]^+$.

HRMS (ESI) calcd for $C_{27}H_{29}N_2O_4Ge$ $[M+H]^+$ 519:1339, found 519:1333.

Tert-butyl (3-{4-[5-(4-methylpiperazin-1-yl)-1H,1'H-[2,5'-bibenzimidazol]-2'-yl]phenoxy}butyl)carbamate

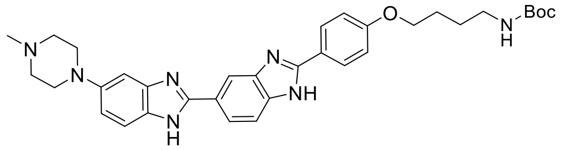

The free base of Hoechst 33258 was prepared by dissolving commercial Hoechst 33258 trihydrochloride (500 mg, 0.936 mmol) in H_2O (30 mL) and adding a solution of K_2CO_3 (388 mg, 2.81 mmol, 3eq.) in H_2O (10 mL) . The precipitate thus formed was isolated by filtration, washed with H_2O and lyophilised from water 1,4-dioxane mixture. The resulting Hoechst 33258 base (397 mg, 0.936 mmol, 1 eq.) was suspended in dry DMF (1 mL). K_2CO_3 (388 mg, 2.81 mmol, 3 eq.) was added followed by 4-(Boc-amino)butyl bromide (283 mg, 1.12 mmol, 1.2 eq.). The reaction was heated at 60 °C for 14 h. The reaction mixture was then cooled to room temperature, DMF was evaporated under reduced pressure. The residue was suspended in DCM and deposited on celite by evaporating the solvent. Product was isolated by flash column chromatography (Büchi Reveleris HP silica 40 g; gradient 20% to 90% CH_2Cl_2 – CH_2Cl_2:MeOH: $NH_{3(aq)}$ [9:1:0.2]) as yellow solid, yield 306 mg (55%).

^{1}H NMR (600 MHz, CD_3OD) δ 8.22 (d, *J* = 1.6 Hz, 1H), 8.01 (d, *J* = 8.8 Hz, 2H), 7.92 (dd, *J* = 8.4, 1.6 Hz, 1H), 7.66 (d, *J* = 8.4 Hz, 1H), 7.50 (d, *J* = 8.7 Hz, 1H), 7.13 (d, *J* = 2.3 Hz, 1H), 7.01 – 7.07 (m, 3H), 4.03 (t, *J* = 6.4 Hz, 2H), 3.23 – 3.33 (m, 4H), 3.11 (t, *J* = 7.0 Hz, 2H), 2.79 – 2.84 (m, 4H), 2.48 (s, 3H), 1.80 (p, *J* = 7.2 Hz, 2H), 1.65 (p, *J* = 7.2 Hz, 2H), δ 1.43 (s, 9H).

^{13}C NMR (126 MHz, CD_3OD) δ 169.6, 162.4, 158.4, 155.2, 153.7, 149.2, 148.3, 140.4, 136.0, 129.5, 125.5, 122.8, 122.4, 116.5, 116.3, 116.0, 113.8, 102.5, 79.9, 68.9, 56.0, 51.4, 45.6, 41.1, 28.8, 27.7.

HRMS (ESI) calcd for $C_{34}H_{42}N_7O_3$ $[M+H]^+$ 596.3344, found 596.3340.

4-{4-[5-(4-methylpiperazin-1-yl)-1*H*,1'*H*-[2,5'-bibenzimidazol]-2'-yl]phenoxy}butan-1-amine (SI-13)

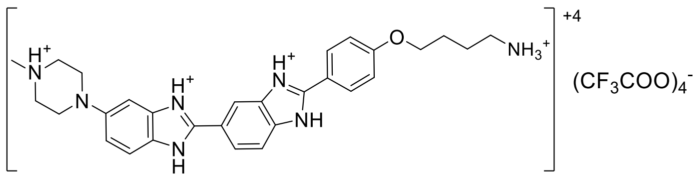

Trifluoroacetic acid (1 mL) was added dropwise to a solution of tert-butyl (3-{4-[5-(4-methylpiperazin-1-yl)-1H,1'H-[2,5'-bibenzimidazol]-2'-yl]phenoxy}butyl)carbamate (306 mg, 0.515 mmol) in CH_2Cl_2 (4 mL). The resulting intense yellow solution was stirred at room temperature for 3h. The reaction mixture was then evaporated to dryness, the residue was re-evaporated three times with MeOH to remove excess trifluoroacetic acid. The residue was lyophilized from aqueous dioxane. Product obtained as trifluoroacetic acid salt ([Hoechst-$(CH_2)_4NH_3]^{4+}[CF_3COO^-]_4$), yield 485 mg (99%), yellow solid. Compound used in further steps without additional purification.

^{1}H NMR (500 MHz, MeOD) δ 8.20 (d, *J* = 1.6 Hz, 1H,), 7.99 (d, *J* = 8.4 Hz, 2H), 7.92 (dd, *J* = 8.4, 1.6 Hz, 1H), 7.77 (d, *J* = 8.5 Hz, 1H), 7.59 (d, *J* = 9.0 Hz, 1H), 7.25 (dd, *J* = 9.0, 2.3 Hz, 1H), 7.19 (d, *J* = 2.3 Hz, 1H), 7.06 (d, *J* = 8.5 Hz, 2H), 4.06 (t, *J* = 6.4 Hz, 2H), 3.97 – 3.64 (m, 4H), 3.30 – 3.07 (m, 4H), 3.03 (t, *J* = 7.2 Hz, 2H), 2.98 (s, 3H), 1.96 – 1.81 (m, 4H).

^{13}C NMR (126 MHz, MeOD) δ 163.6, 155.4, 150.4, 150.1, 140.6, 138.3, 134.6, 130.4, 128.2, 123.9, 119.7, 119.4, 119.1, 116.6, 116.4, 115.5, 115.0, 100.9, 68.7, 54.5, 48.3, 43.5, 40.5, 27.1, 25.5.

HRMS (ESI) calcd for $C_{29}H_{34}N_7O$ $[M+H]^+$ 496.2819, found 496.2820.

General procedure for the Dye-Hoechst conjugates:

The corresponding dye (0.03 mmol, 1 eq.), DIPEA (52 µL, 0.3 mmol, 10 eq.), TSTU (14 mg, 0.046 mmol, 1.5 eq.) were dissolved in 200 µL DMF and stirred at room temperature for 2 hours. A solution of **SI-13** (43mg, 0.045 mmol, 1.5eq.) in 150 µL DMF were added to the reaction mixture and stirring continued for 6-12 h. Reaction was monitored by HPLC analysis. The solvent was evaporated *in vacuo* at room temperature and the products were purified by preparative HPLC (preparative column: Eurospher 100 C18, 5 µm, 250 × 20 mm; solvent A: acetonitrile, solvent B: H_2O + 0.2% v/v HCOOH; temperature 25 °C, gradient A:B - 5 min 20:80 isocratic, 5-30 min 20:80 to 70:30 gradient) and lyophilised from 1,4-dioxane and water mixtures.

6-580CP-Hoechst

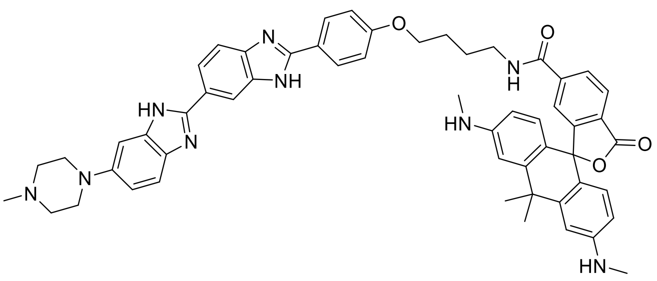

Was prepared from 16 mg of **6-580CP-COOH** (0.03 mmol) [14] (trifluoroacetic acid salt). Product yield 10.3 mg (38%) of violet solid. Reaction was carried out in DMSO instead of DMF.
Analytical HPLC: t_R = 9.10 min (Knauer Eurospher II 100-5 C18, 5 µm, 150×4 mm (Article No. 15DE181E2J, Knauer) analytical column, solvent A: acetonitrile, solvent B: H_2O + 0.2% v/v TFA, temperature 20 °C, gradient A:B 3 min 20:80 isocratic, 3-16 min 20:80 to 70:30 gradient, 1.2 mL/min flow).
^{1}H NMR (400 MHz, CD_3OD) δ 8.34 (d, *J* = 8.2 Hz, 1H), 8.31 (d, *J* = 1.7 Hz, 1H), 8.14 (dd, *J* = 8.2, 1.8 Hz, 1H), 8.04 (d, *J* = 8.8 Hz, 2H), 7.98 (dd, *J* = 8.6, 1.7 Hz, 1H), 7.83 (d, *J* = 8.6 Hz, 1H), 7.76 (d, *J* = 1.8 Hz, 1H), 7.67 (d, *J* = 9.0 Hz, 1H), 7.34 (dd, *J* = 9.0, 2.2 Hz, 1H), 7.27 (d, *J* = 2.2 Hz, 1H), 7.11 (d, *J* = 2.3 Hz, 2H), 7.09 (d, *J* = 8.8 Hz, 2H), 6.97 (d, *J* = 9.2 Hz, 2H), 6.61 (dd, *J* = 9.2, 2.2 Hz, 2H), 4.09 (t, *J* = 5.9 Hz, 2H), 3.73 – 4.05 (m, 4H), 3.49 (t, *J* = 6.6 Hz, 2H), 3.33 – 3.44 (m, 2H), 3.18 – 3.27 (m, 2H), 3.04 (s, 6H), 3.01 (s, 3H), 1.82 – 1.94 (m, 4H), 1.82 (s, 3H), 1.71 (s, 3H).
^{13}C NMR (101 MHz, CD_3OD) δ 168.1, 167.5, 166.6, 163.6, 163.0, 162.7, 159.3, 158.7, 156.0, 150.9, 150.5, 139.3, 138.9, 138.5, 135.0, 132.5, 130.24, 130.22, 129.2, 128.7, 123.7, 122.0, 120.5, 119.5, 119.1, 116.5, 116.4, 115.6, 115.4, 113.9, 112.2, 101.2, 69.0, 54.6, 43.6, 42.7, 40.9, 35.3, 32.1, 30.0, 27.7, 27.0.
ESI-MS, positive mode: m/z = 906.4 $[M+H]^+$.
HRMS (ESI) calcd for $C_{55}H_{56}N_9O_4$ $[M+H]^+$ 906.4450, found 906.4454.

5-580CP-Hoechst

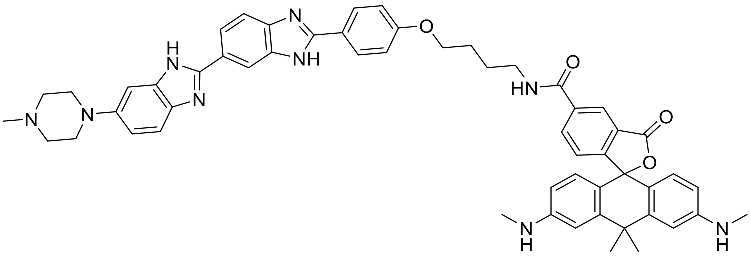

Was prepared from 16 mg (0.03 mmol) of **5-580CP-COOH** (trifluoroacetic acid salt). Product yield 18 mg (66%) of violet solid. Reaction was carried out in DMSO instead of DMF. Analytical HPLC: t_R = 9.28 min (Knauer Eurospher II 100-5 C18, 5 µm, 150×4 mm (Article No. 15DE181E2J, Knauer) analytical column, solvent A: acetonitrile, solvent B: H_2O + 0.2% v/v

TFA, temperature 20 °C, gradient A:B 3 min 20:80 isocratic, 3-16 min 20:80 to 70:30 gradient, 1.2 mL/min flow).

^{1}H NMR (400 MHz, CD_3OD) δ 8.73 (d, *J* = 1.8 Hz, 1H). 8.28 (d, *J* = 1.7 Hz, 1H), 8.18 (dd, *J* = 7.9, 1.9 Hz, 1H), 8.03 – 8.09 (m, 2H), 7.96 (dd, *J* = 8.5, 1.8 Hz, 1H), 7.80 (d, *J* = 8.5 Hz, 1H), 7.67 (d, *J* = 9.0 Hz, 1H), 7.42 (d, *J* = 8.0 Hz, 1H), 7.33 (dd, *J* = 9.0, 2.2 Hz, 1H), 7.27 (d, *J* = 2.2 Hz, 1H), 7.11 – 7.15 (m, 2H), 7.09 (d, *J* = 2.3 Hz, 2H), 6.95 (d, *J* = 9.2 Hz, 2H), 6.59 (dd, *J* = 9.2, 2.3 Hz, 2H), 4.15 (t, *J* = 5.8 Hz, 2H), 3.68 – 4.04 (m, 4H), 3.58 (d, *J* = 6.6 Hz, 2H), 3.33 – 3.54 (m, 4H), 3.03 (s, 6H), 3.01 (s, 3H), 1.88 – 1.99 (m, 4H), 1.80 (s, 3H), 1.69 (s, 3H).

^{13}C NMR (101 MHz, CD_3OD) δ 168.5, 167.6, 166.5, 163.4, 163.2, 162.8, 159.2, 156.2, 151.2, 150.3, 142.0, 138.3, 137.0, 135.3, 133.1, 132.0, 131.7, 131.0, 130.1, 129.1, 123.4, 121.8, 121., 119.6, 119.0, 116.5, 116.4, 115.6, 115.4, 113.9, 112.0, 101.3, 69.0, 54.7, 48.7, 43.6, 42.7, 40.9, 35.4, 31.9, 30.0, 27.8, 27.1.

ESI-MS, positive mode: m/z = 906.4 $[M+H]^+$.

HRMS (ESI) calcd for $C_{55}H_{56}N_9O_4$ $[M+H]^+$ 906.4450, found 906.4456.

6-TMR-Hoechst

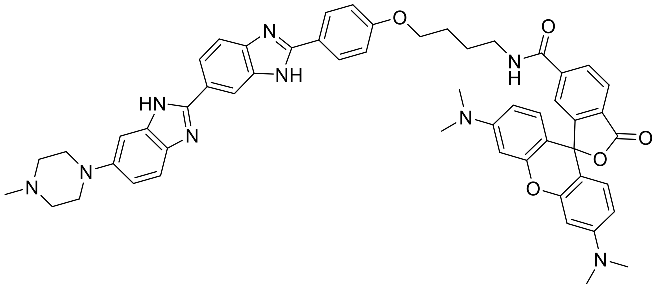

Was prepared from 13 mg (0.03 mmol) of **6-TMR-COOH.** Product yield 17.5 mg (65%) of deep red solid. Analytical HPLC: t_R = 9.28 min (Knauer Eurospher II 100-5 C18, 5 µm, 150×4 mm (Article No. 15DE181E2J, Knauer) analytical column, solvent A: acetonitrile, solvent B: H_2O + 0.2% v/v TFA, temperature 20 °C, gradient A:B 3 min 20:80 isocratic, 3-16 min 20:80 to 70:30 gradient, 1.2 mL/min flow).

^{1}H NMR (400 MHz, CD_3OD + CF_3COOD) δ 8.48 (s, 1H), 8.39 (d, *J* = 8.2 Hz, 1H), 8.19 (dd, *J* = 8.2, 1.8 Hz, 1H), 8.15 (d, *J* = 8.6 Hz, 1H), 8.10 (d, *J* = 8.6 Hz, 2H), 7.98 (d, *J* = 8.5 Hz, 1H), 7.83 (d, *J* = 1.7 Hz, 1H), 7.72 (d, *J* = 8.9 Hz, 1H), 7.39 (d, *J* = 9.0 Hz, 1H), 7.32 (s, 1H), 7.18 (d, *J* = 8.6 Hz, 2H), 7.12 (d, *J* = 9.5 Hz, 2H), 7.01 (dd, *J* = 9.5, 2.4 Hz, 2H), 6.93 (d, *J* = 2.4 Hz, 2H), 4.14 (t, *J* = 6.0 Hz, 2H), 3.90 – 3.98 (m, 2H), 3.65 – 3.72 (m, 2H), 3.49 (t, *J* = 6.8 Hz, 2H), 3.31 – 3.38 (m, 2H), 3.27 (s, 12H), 3.14 – 3.22 (m, 2H), 3.00 (s, 3H), 1.79 – 1.93 (m, 4H).

^{13}C NMR (101 MHz, CD_3OD+ CF_3COOD) δ 168.0, 167.3, 165.0, 160.5, 159.0, 158.9, 154.6, 150.9, 149.6, 139.5, 137.9, 135.5, 134.8, 134.6, 132.8, 132.0, 131.1, 130.3, 130.0, 128.0, 125.6, 121.0, 119.7, 116.9, 116.8, 116.5, 115.7, 115.5, 115.0, 114.9, 101.0, 97.4, 69.3, 54.6, 48.4, 43.6, 40.9, 40.8, 27.6, 27.0.

ESI-MS, positive mode: m/z = $[M+H]^+$ 908.5.

HRMS (ESI) calcd for $C_{54}H_{54}N_9O_5$ $[M+H]^+$ 908.4242, found 908.4282.

5-TMR-Hoechst

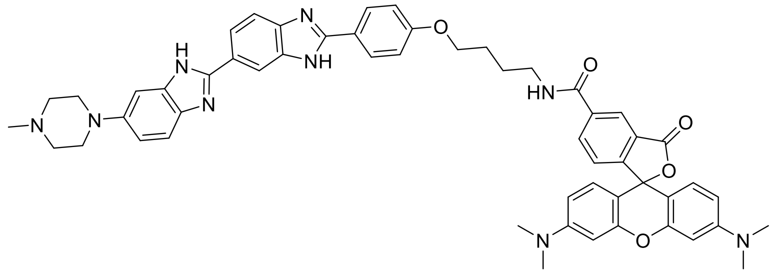

Was prepared from 13 mg (0.03 mmol) of **5-TMR-COOH.** Product yield 16.8 mg (62%) of deep red solid. Analytical HPLC: t_R = 9.62 min (Knauer Eurospher II 100-5 C18, 5 µm, 150×4 mm (Article No. 15DE181E2J, Knauer) analytical column, solvent A: acetonitrile, solvent B: H_2O + 0.2% v/v TFA, temperature 20 °C, gradient A:B 3 min 20:80 isocratic, 3-16 min 20:80 to 70:30 gradient, 1.2 mL/min flow).

^{1}H NMR (400 MHz, CD_3OD+ CF_3COOD) δ 8.79 (d, J = 1.8 Hz, 1H) 8.43 (d, J = 1.6 Hz, 1H), 8.26 (dd, J = 7.9, 1.9 Hz, 1H), 8.07 – 8.16 (m, 3H), 7.94 (d, J = 8.6 Hz, 1H), 7.72 (d, J = 9.0 Hz, 1H), 7.52 (d, J = 8.0 Hz, 1H), 7.39 (dd, J = 9.1, 2.2 Hz, 1H), 7.32 (d, J = 2.2 Hz, 1H), 7.21 (d, J = 9.0 Hz, 2H), 7.12 (d, J = 9.5 Hz, 2H), 7.02 (dd, J = 9.5, 2.5 Hz, 2H), 6.94 (d, J = 2.4 Hz, 2H), 4.20 (t, J = 5.9 Hz, 2H), 3.90 – 4.03 (m, 2H), 3.64 – 3.75 (m, 2H), 3.59 (t, J = 6.5 Hz, 2H), 3.32 – 3.43 (m, 2H), 3.28 (s, 12H), 3.11 – 3.24 (m, 2H), 3.02 (s, 3H), 1.88 – 2.01 (m, 4H).

^{13}C NMR (101 MHz, CD_3OD+ CF_3COOD) δ 168.2; 167.4; 165.3; 160.6, 159.0, 158.9, 154.4, 150.9, 149.5, 138.1, 137.8, 134.6, 132.9, 132.3, 132.0, 131.9, 131.3, 131.3, 128.0, 125.9, 121.3, 119.8, 118.5, 117.1, 116.5, 115.7, 115.6, 115.5, 114.9, 114.7, 100.9, 97.4, 69.4, 54.6, 48.4, 43.6, 40.9, 40.8, 27.7, 27.1.

ESI-MS, positive mode: m/z = 908.5 $[M+H]^+$.

HRMS (ESI) calcd for $C_{54}H_{54}N_9O_5$ $[M+H]^+$ 908.4242, found 906.4250.

6-SiR-Hoechst

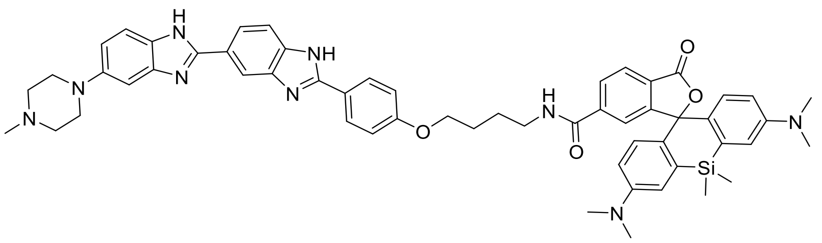

Was prepared from 14 mg (0.03 mmol) of **6-SiR-COOH.** Product yield 13.4 mg (47%) of light blue solid. Analytical HPLC: t_R = 10.70 min (Knauer Eurospher II 100-5 C18, 5 µm, 150×4 mm (Article No. 15DE181E2J, Knauer) analytical column, solvent A: acetonitrile, solvent B: H_2O +

0.2% v/v TFA, temperature 20 °C, gradient A:B 3 min 20:80 isocratic, 3-16 min 20:80 to 70:30 gradient, 1.2 mL/min flow).

^{1}H NMR (400 MHz, CD_3OD+ CF_3COOD) δ 8.46 (d, *J* = 1.7 Hz, 1H), 8.28 (d, *J* = 8.2 Hz, 1H), 8.15 (dd, *J* = 8.7, 1.7 Hz, 1H), 8.06 – 8.12 (m, 3H), 7.98 (d, *J* = 8.7 Hz, 1H), 7.69 – 7.74 (m, 2H), 7.39 (dd, *J* = 9.1, 2.2 Hz, 1H), 7.33 (d, *J* = 2.8 Hz, 2H), 7.31 (d, *J* = 2.2 Hz, 1H), 7.17 (d, *J* = 9.0 Hz, 2H), 6.97 (d, *J* = 9.5 Hz, 2H), 6.75 (dd, *J* = 9.6, 2.8 Hz, 2H), 4.12 (t, *J* = 6.0 Hz, 2H), 3.90 – 3.99 (m, 2H), 3.63 – 3.72 (m, 2H), 3.46 (t, *J* = 6.8 Hz, 2H), 3.30 – 3.40 (m, 2H), 3.28 (s, 12H), 3.13 – 3.23 (m, 2H), 3.00 (s, 3H), 1.77 – 1.91 (m, 4H), 0.63 (s, 3H), 0.57 (s, 3H).

^{13}C NMR (101 MHz, CD_3OD+ CF_3COOD) δ 168.1, 167.8, 165.1, 163.3, 154.8, 154.5, 150.9, 149.5, 148.4, 140.7, 139.0, 137.6, 135.4, 134.5, 134.2, 132.0, 131.2, 130.1, 129.8, 128.9, 127.9, 125.6, 121.8, 121.0, 119.7, 116.9, 116.6, 116.5, 115.7, 115.4, 114.9, 100.9, 69.3, 54.6, 48.4 (from HSQC, overlapped with solvent peak), 43.6, 41.1, 40.8, 27.6, 27.0, -0.8, -1.7.

HRMS (ESI) calcd for $C_{56}H_{60}N_9O_4Si$ $[M+H]^+$ 950.4532, found 950.4567.

5-SiR-Hoechst

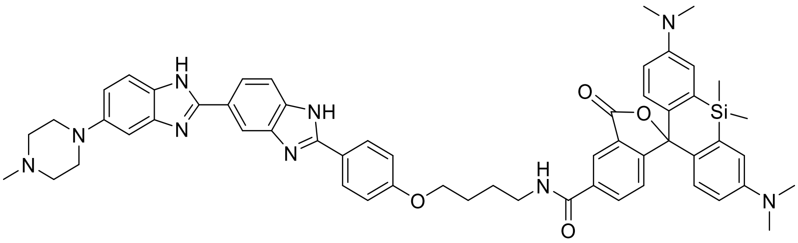

Was prepared from 14 mg (0.03 mmol) of **5-SiR-COOH.** Product yield 12.2 mg (43%) of light blue solid. Analytical HPLC: t_R = 10.05 min (Knauer Eurospher II 100-5 C18, 5 µm, 150×4 mm (Article No. 15DE181E2J, Knauer) analytical column, solvent A: acetonitrile, solvent B: H_2O + 0.2% v/v TFA, temperature 20 °C, gradient A:B 3 min 20:80 isocratic, 3-16 min 20:80 to 70:30 gradient, 1.2 mL/min flow).

^{1}H NMR (400 MHz, CD_3OD+CF_3COOD) δ 8.64 (d, *J* = 1.8 Hz, 1H), 8.25 (d, *J* = 1.6 Hz, 1H), 8.17 (dd, *J* = 8.0, 1.8 Hz, 1H), 8.00 (d, *J* = 8.8 Hz, 2H), 7.96 (dd, *J* = 8.6, 1.6 Hz, 1H), 7.80 (d, *J* = 8.5 Hz, 1H), 7.64 (d, *J* = 9.0 Hz, 1H), 7.37 (d, *J* = 8.0 Hz, 1H), 7.31 (dd, *J* = 9.1, 2.2 Hz, 1H), 7.20 – 7.28 (m, 3H), 7.08 (d, *J* = 8.9 Hz, 2H), 6.90 (d, *J* = 9.4 Hz, 2H), 6.69 (dd, *J* = 9.5, 2.8 Hz, 2H), 4.09 (t, *J* = 5.8 Hz, 2H), 3.85 – 3.98 (m, 2H), 3.64 – 3.78 (m, 2H), 3.54 (t, *J* = 6.4 Hz, 2H), 3.30 – 3.41 (m, 2H), 3.21 (s, 12H), 3.09 – 3.20 (m, 2H), 3.00 (s, 3H), 1.83 – 1.94 (m, 4H), 0.61 (s, 3H), 0.54 (s, 3H).

^{13}C NMR (101 MHz, CD_3OD+ CF_3COOD) δ 168.4, 164.4, 163.9, 155.5, 154.3, 150.5, 150.4, 148.1, 146.9, 140.5, 138.9, 138.4, 136.8, 134.8, 132.3, 131.7, 130.8, 130.4, 130.0, 129.8, 128.4, 126.1, 124.0, 121.1, 119.6, 119.5, 119.2, 116.5, 116.4, 115.6, 115.1, 101.0, 69.1, 54.6, 48.5, 43.6, 40.9, 40.8, 27.7, 27.1, -0.6, -1.8.

ESI-MS, positive mode: m/z = 950.4 $[M+H]^+$.

HRMS (ESI) calcd for $C_{56}H_{60}N_9O_4Si$ $[M+H]^+$ 950.4532, found 950.4532.

6-CP610-Hoechst

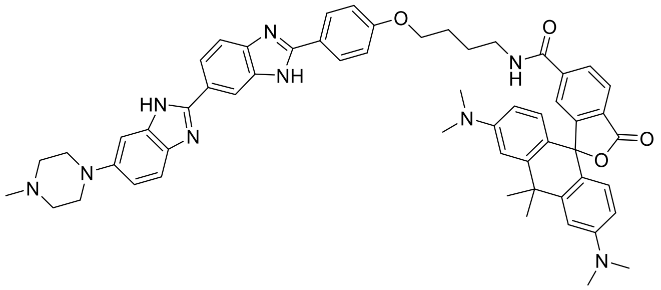

Was prepared from 13.7 mg (0.03 mmol) of **6-610CP-COOH** [14]**.** Product yield 15.7 mg (56%) of purple-blue solid. Analytical HPLC: t_R = 9.93 min (Knauer Eurospher II 100-5 C18, 5 µm, 150×4 mm (Article No. 15DE181E2J, Knauer) analytical column, solvent A: acetonitrile, solvent B: H_2O + 0.2% v/v TFA, temperature 20 °C, gradient A:B 3 min 20:80 isocratic, 3-16 min 20:80 to 70:30 gradient, 1.2 mL/min flow).

^{1}H NMR (400 MHz, CD_3OD+ CF_3COOD) δ 8.56 (d, *J* = 1.5 Hz, 1H), 8.36 (d, *J* = 8.3 Hz, 1H), 8.23 (dd, *J* = 8.6, 1.5 Hz, 1H), 8.12 – 8.20 (m, 3H), 8.05 (d, *J* = 8.7 Hz, 1H), 7.73 – 7.80 (m, 2H), 7.44 (dd, *J* = 9.1, 2.2 Hz, 1H), 7.36 (d, *J* = 2.2 Hz, 1H), 7.20 – 7.28 (m, 4H), 7.03 (d, *J* = 9.4 Hz, 2H), 6.81 (dd, *J* = 9.4, 2.5 Hz, 2H), 4.19 (t, *J* = 6.0 Hz, 2H), 3.93 – 4.02 (m, 2H), 3.66 – 3.73 (m, 2H), 3.50 (t, *J* = 6.8 Hz, 2H), 3.34 – 3.41 (m, 2H), 3.33 (s, 12H), 3.18 – 3.25 (m, 2H), 3.01 (s, 3H), 1.90 – 1.97 (m, 2H), 1.88 (s, 3H), 1.81 – 1.87 (m, 2H), 1.77 (s, 3H).

^{13}C NMR (101 MHz, CD_3OD+ CF_3COOD) δ 168.1, 167.4, 165.4, 158.4, 157.9, 154.3, 151.0, 149.4, 139.3, 138.9, 137.9, 135.0, 134.5, 132.6, 131.4, 130.3, 129.2, 127.9, 126.1, 121.9, 121.5, 119.9, 118.3, 117.1, 116.5, 115.9, 115.7, 115.5, 115.0, 114.0, 112.0, 101.0, 69.4, 54.6, 48.4, 43.6, 43.2, 41.0, 40.8, 35.7, 32.6, 27.6, 27.0.

ESI-MS, positive mode: m/z = 934.5 $[M+H]^+$.

HRMS (ESI) calcd for $C_{57}H_{60}N_9O_4$ $[M+H]^+$ 934.4763, found 934.4765.

5-CP610-Hoechst

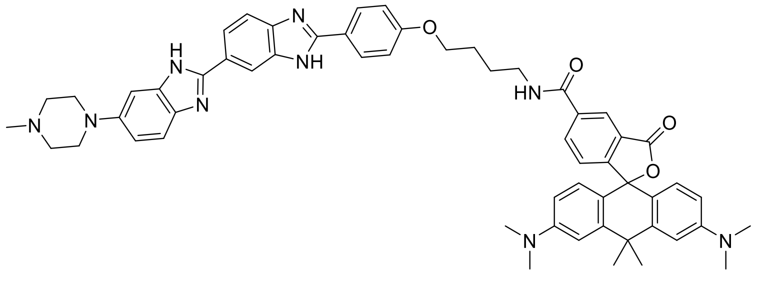

Was prepared from 13.7 mg (0.03 mmol) of **5-CP610-COOH.** Product yield 14.3 mg (50%) of purple-blue solid. Analytical HPLC: t_R = 10.02 min (Knauer Eurospher II 100-5 C18, 5 µm, 150×4 mm (Article No. 15DE181E2J, Knauer) analytical column, solvent A: acetonitrile,

solvent B: H_2O + 0.2% v/v TFA, temperature 20 °C, gradient A:B 3 min 20:80 isocratic, 3-16 min 20:80 to 70:30 gradient, 1.2 mL/min flow).

^{1}H NMR (400 MHz, CD_3OD+ CF_3COOD) δ 8.73 (d, J = 1.8 Hz, 1H), 8.53 (d, 1H), 8.19 – 8.23 (m, 2H), 8.16 (d, J = 8.5 Hz, 2H), 8.04 (d, J = 8.6 Hz, 1H), 7.75 (d, J = 9.1 Hz, 1H), 7.41 – 7.46 (m, 2H), 7.34 (d, J = 2.1 Hz, 1H), 7.28 (d, J = 8.6 Hz, 2H), 7.21 (d, J = 2.5 Hz, 2H), 7.00 (d, J = 9.4 Hz, 2H), 6.79 (dd, J = 9.4, 2.5 Hz, 2H), 4.24 (t, J = 6.0 Hz, 2H), 3.94 – 4.01 (m, 2H), 3.65 – 3.72 (m, 2H), 3.57 (t, J = 6.7 Hz, 2H), 3.34 – 3.44 (m, 2H), 3.31 (s, 12H), 3.17 – 3.24 (m, 2H), 3.00 (s, 3H), 1.89 – 2.00 (m, 4H), 1.86 (s, 3H), 1.74 (s, 3H).

^{13}C NMR (101 MHz, CD_3OD+ CF_3COOD) δ 168.4, 167.4, 165.4, 158.4, 157.9, 151.0, 149.5, 141.9, 137.7, 137.0, 134.9, 134.6, 133.0, 132.1, 131.8, 131.4, 131.1, 128.0, 126.0, 121.7, 121.5, 119.9, 117.5, 117.1, 116.5, 116.1, 115.7, 115.0, 114.6, 114.0, 112.0, 101.0, 69.5, 54.6, 48.4, 43.6, 43.2, 41.0, 40.8, 35.8, 32.3, 27.7, 27.1.

ESI-MS, positive mode: m/z = 934.4 $[M+H]^+$.

HRMS (ESI) calcd for $C_{57}H_{60}N_9O_4$ $[M+H]^+$ 934.4763, found 934.4765.

6-GeR-Hoechst

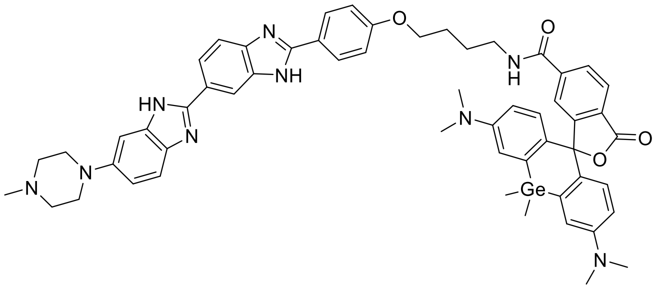

Was prepared from 15 mg (0.029 mmol) of **6-GeR-COOH [13].** Product yield 22 mg (76%) of light blue solid. Analytical HPLC: t_R = 9.83 min (Knauer Eurospher II 100-5 C18, 5 µm, 150×4 mm (Article No. 15DE181E2J, Knauer) analytical column, solvent A: acetonitrile, solvent B: H_2O + 0.2% v/v TFA, temperature 20 °C, gradient A:B 3 min 20:80 isocratic, 3-16 min 20:80 to 70:30 gradient, 1.2 mL/min flow).

^{1}H NMR (400 MHz, CD_3OD+ CF_3COOD) δ 8.47 (d, J = 2.0 Hz, 1H), 8.28 (d, J = 8.2 Hz, 1H), 8.14 (dd, J = 8.6, 2.0 Hz, 1H), 8.08 – 8.12 (m, 3H), 7.97 (d, J = 8.6 Hz, 1H), 7.70 – 7.74 (m, 2H), 7.39 (dd, J = 9.1, 2.2 Hz, 1H), 7.31 (d, J = 2.2 Hz, 1H), 7.28 (d, J = 2.8 Hz, 2H), 7.18 (d, J = 8.9 Hz, 2H), 6.97 (d, J = 9.6 Hz, 2H), 6.71 (dd, J = 9.6, 2.8 Hz, 2H), 4.13 (t, J = 6.0 Hz, 2H), 3.90 – 3.99 (m, 2H), 3.64 – 3.71 (m, 2H), 3.47 (t, J = 6.8 Hz, 2H), 3.30 – 3.38 (m, 2H), 3.27 (s, 12H), 3.15 – 3.24 (m, 2H), 2.99 (s, 3H), 1.79 – 1.91 (m, 4H), 0.77 (s, 3H), 0.73 (s, 3H).

^{13}C NMR (101 MHz, CD_3OD+ CF_3COOD) δ 168.1, 167.7, 165.0, 154.7, 154.6, 152.5, 150.9, 149.7, 141.1, 138.9, 138.1, 135.9, 134.6, 134.4, 132.1, 131.1, 129.9, 129.6, 128.8, 128.0, 125.5, 121.5, 120.9, 119.7, 118.8, 116.9, 116.5, 115.9, 115.7, 115.1, 115.0, 101.0, 69.3, 54.6, 48.4, 43.6, 41.0, 40.8, 27.6, 27.0, -0.6, -1.4.

ESI-MS, positive mode: m/z = 996.4 $[M+H]^+$.

HRMS (ESI) calcd for $C_{56}H_{60}N_9O_4Ge$ $[M+H]^+$ 996.3989, found 996.3973.

5-GeR-Hoechst

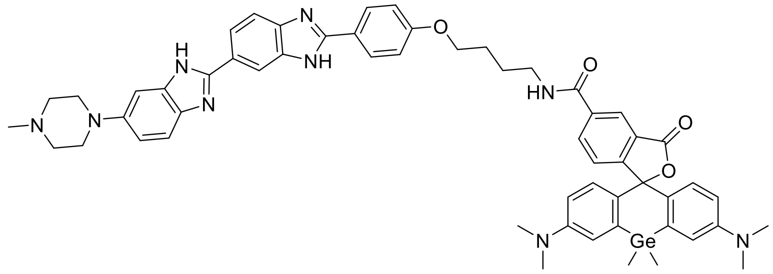

Was prepared from 15 mg (0.029 mmol) of **5-GeR-COOH.** Product yield 20 mg (69%) of light blue solid. Analytical HPLC: t_R = 10.03 min (Knauer Eurospher II 100-5 C18, 5 µm, 150×4 mm (Article No. 15DE181E2J, Knauer) analytical column, solvent A: acetonitrile, solvent B: H_2O + 0.2% v/v TFA, temperature 20 °C, gradient A:B 3 min 20:80 isocratic, 3-16 min 20:80 to 70:30 gradient, 1.2 mL/min flow).

^1H NMR (400 MHz, CD_3OD+ CF_3COOD) δ 8.70 (d, J = 1.8 Hz, 1H), 8.55 (d, J = 1.6 Hz, 1H), 8.19 – 8.26 (m, 2H), 8.17 (d, J = 9.0 Hz, 2H), 8.04 (d, J = 8.7 Hz, 1H), 7.76 (d, J = 9.1 Hz, 1H), 7.42 – 7.47 (m, 2H), 7.36 (d, J = 2.2 Hz, 1H), 7.31 (d, J = 2.8 Hz, 2H), 7.28 (d, J = 9.0 Hz, 2H), 7.00 (d, J = 9.6 Hz, 2H), 6.74 (dd, J = 9.6, 2.8 Hz, 2H), 4.23 (t, J = 6.0 Hz, 2H), 3.93 – 4.01 (m, 2H), 3.66 – 3.73 (m, 2H), 3.58 (t, J = 6.7 Hz, 2H), 3.33 – 3.42 (m, 2H), 3.29 (s, 12H), 3.17 – 3.26 (m, 2H), 3.02 (s, 3H), 1.88 – 2.00 (m, 4H), 0.78 (s, 3H), 0.74 (s, 3H).

^{13}C NMR (101 MHz, CD_3OD+ CF_3COOD) δ 168.4, 167.7, 165.4, 154.4, 154.4, 152.4, 151.0, 149.4, 140.9, 137.0, 136.7, 134.8, 134.6, 132.4, 131.9, 131.7, 131.4, 130.6, 128.0, 126.0, 121.6, 121.5, 119.9, 118.4, 117.1, 116.5, 116.1, 115.7, 115.6, 115.0, 115.0, 101.0, 69.4, 54.6, 48.4, 43.6, 41.1, 40.8, 27.7, 27.1, -0.5, -1.6.

ESI-MS, positive mode: m/z = 996.4 $[M+H]^+$.

HRMS (ESI) calcd for $C_{56}H_{60}N_9O_4Ge$ $[M+H]^+$ 996.3989, found 996.3958.

Supplementary references

1. Pjura PE, Grzeskowiak K, Dickerson RE. Binding of Hoechst 33258 to the minor groove of B-DNA. *J Mol Biol* 1987, **197**(2)**:** 257-271.
2. Breusegem SY, Clegg RM, Loontiens FG. Base-sequence specificity of Hoechst 33258 and DAPI binding to five (A/T)4 DNA sites with kinetic evidence for more than one high-affinity Hoechst 33258-AATT complex. *J Mol Biol* 2002, **315**(5)**:** 1049-1061.
3. Bach M, Grigat S, Pawlik B, Fork C, Utermohlen O, Pal S*, et al.* Fast set-up of doxycycline-inducible protein expression in human cell lines with a single plasmid based on Epstein-Barr virus replication and the simple tetracycline repressor. *FEBS J* 2007, **274**(3)**:** 783-790.
4. Lukinavicius G, Lavogina D, Orpinell M, Umezawa K, Reymond L, Garin N*, et al.* Selective chemical crosslinking reveals a Cep57-Cep63-Cep152 centrosomal complex. *Curr Biol* 2013, **23**(3)**:** 265-270.
5. Schindelin J, Arganda-Carreras I, Frise E, Kaynig V, Longair M, Pietzsch T*, et al.* Fiji: an open-source platform for biological-image analysis. *Nat Methods* 2012, **9**(7)**:** 676-682.
6. Carpenter AE, Jones TR, Lamprecht MR, Clarke C, Kang IH, Friman O*, et al.* CellProfiler: image analysis software for identifying and quantifying cell phenotypes. *Genome Biol* 2006, **7**(10)**:** R100.
7. Richardson WH. Bayesian-Based Iterative Method of Image Restoration*. *J Opt Soc Am* 1972, **62**(1)**:** 55-59.
8. Gottlieb HE, Kotlyar V, Nudelman A. NMR Chemical Shifts of Common Laboratory Solvents as Trace Impurities. *J Org Chem* 1997, **62**(21)**:** 7512-7515.
9. Lukinavicius G, Umezawa K, Olivier N, Honigmann A, Yang G, Plass T*, et al.* A near-infrared fluorophore for live-cell super-resolution microscopy of cellular proteins. *Nat Chem* 2013, **5**(2)**:** 132-139.
10. Pastierik T, Sebej P, Medalova J, Stacko P, Klan P. Near-infrared fluorescent 9-phenylethynylpyronin analogues for bioimaging. *J Org Chem* 2014, **79**(8)**:** 3374-3382.
11. Grimm JB, Sung AJ, Legant WR, Hulamm P, Matlosz SM, Betzig E*, et al.* Carbofluoresceins and carborhodamines as scaffolds for high-contrast fluorogenic probes. *ACS Chem Biol* 2013, **8**(6)**:** 1303-1310.
12. Grimm JB, Klein T, Kopek BG, Shtengel G, Hess HF, Sauer M*, et al.* Synthesis of a Far-Red Photoactivatable Silicon-Containing Rhodamine for Super-Resolution Microscopy. *Angew Chem Int Ed Engl* 2016, **55**(5)**:** 1723-1727.
13. Butkevich AN, Belov VN, Kolmakov K, Sokolov VV, Shojaei H, Sidenstein SC*, et al.* Hydroxylated Fluorescent Dyes for Live-Cell Labeling: Synthesis, Spectra and Super-Resolution STED. *Chemistry – A European Journal* 2017, **23**(50)**:** 12114-12119.
14. Butkevich AN, Mitronova GY, Sidenstein SC, Klocke JL, Kamin D, Meineke DN*, et al.* Fluorescent Rhodamines and Fluorogenic Carbopyronines for Super-Resolution STED Microscopy in Living Cells. *Angew Chem Int Ed Engl* 2016, **55**(10)**:** 3290-3294.

NMR copies

5-610CP-COOH

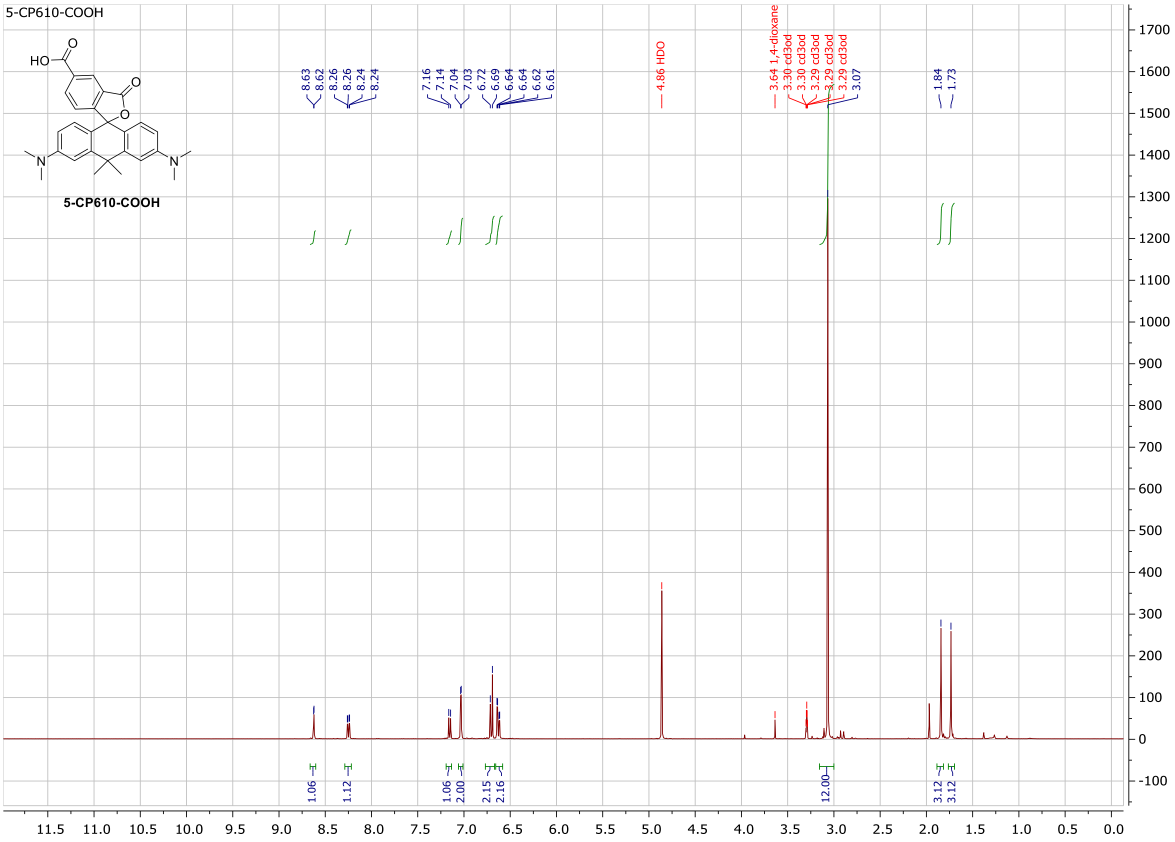

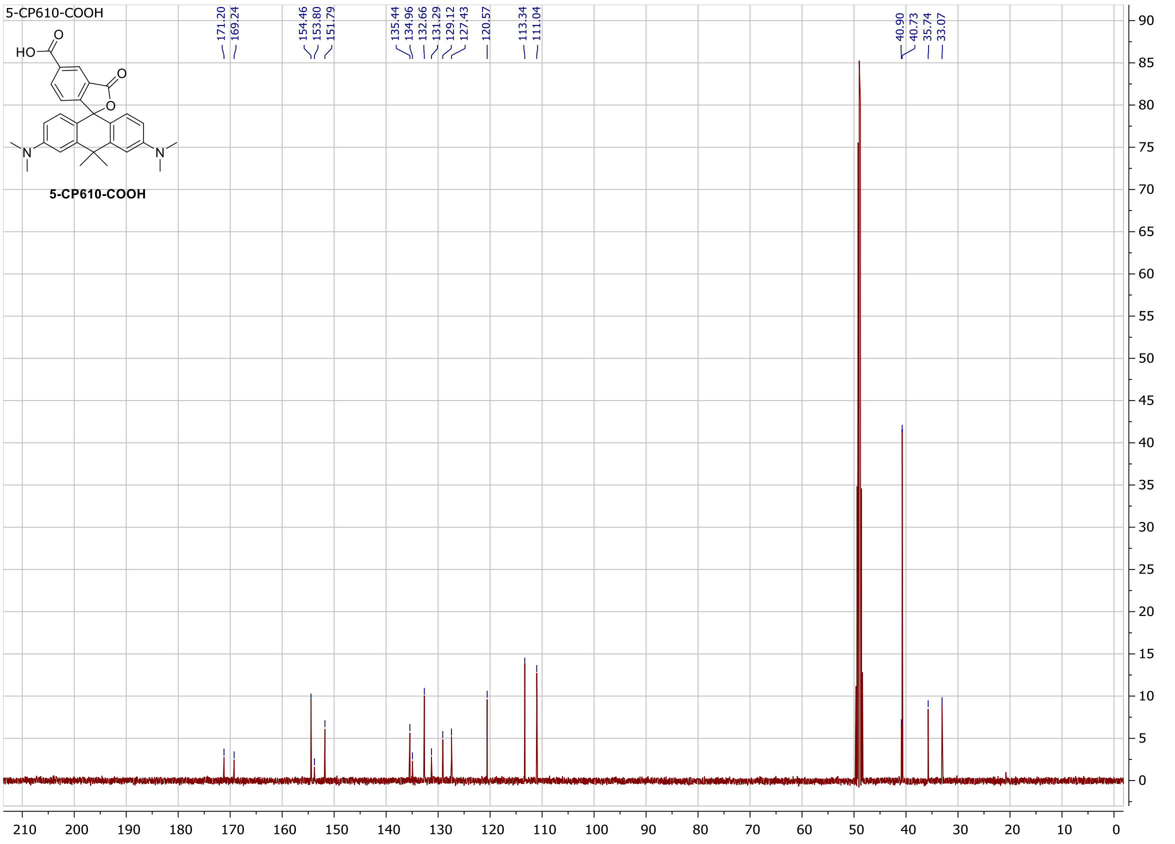

5-SiR-COOH

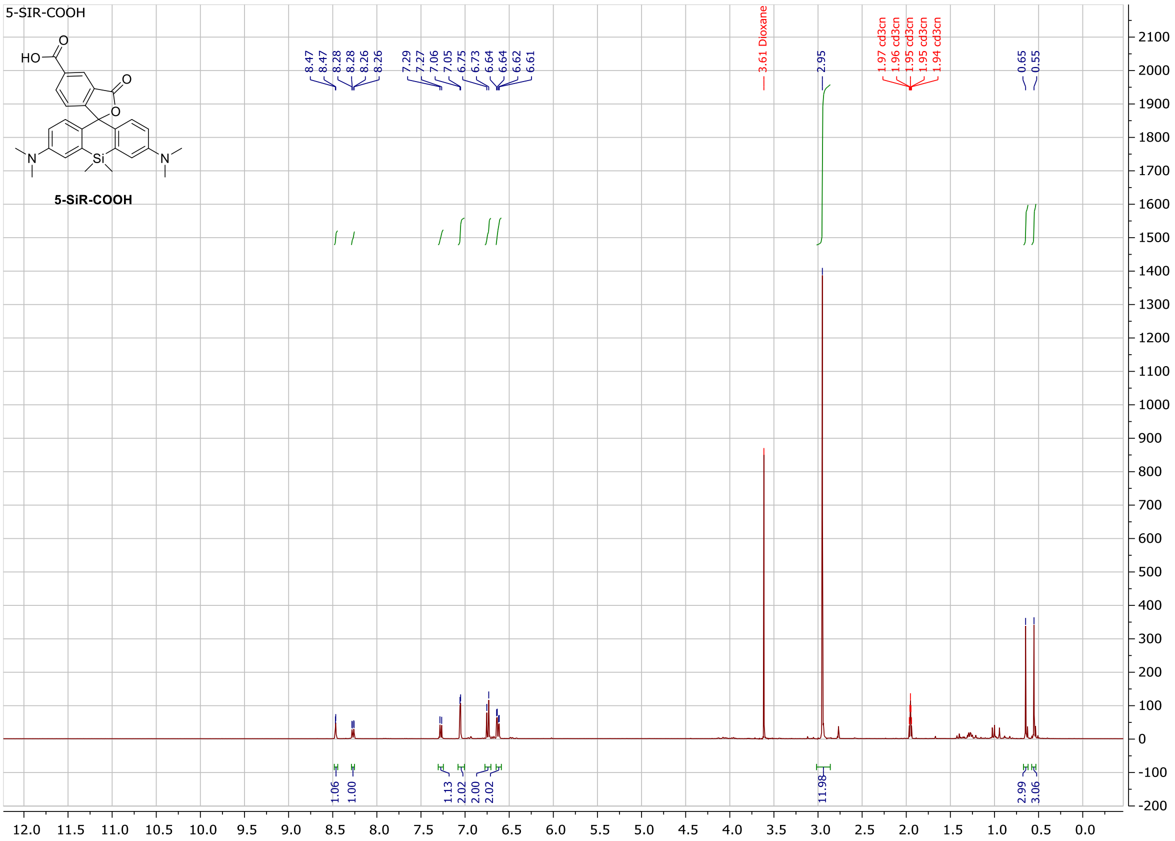
5-SIR-COOH
5-SiR-COOH
8.47 8.47 8.28 8.28 8.26 8.26 7.29 7.27 7.06 7.05 6.75 6.73 6.64 6.64 6.62 6.61
3.61 Dioxane
2.95
1.97 cd3cn 1.96 cd3cn 1.95 cd3cn 1.95 cd3cn 1.94 cd3cn
0.65 0.55
1.06 1.00 1.13 2.02 2.00 2.02 11.98 2.99 3.06

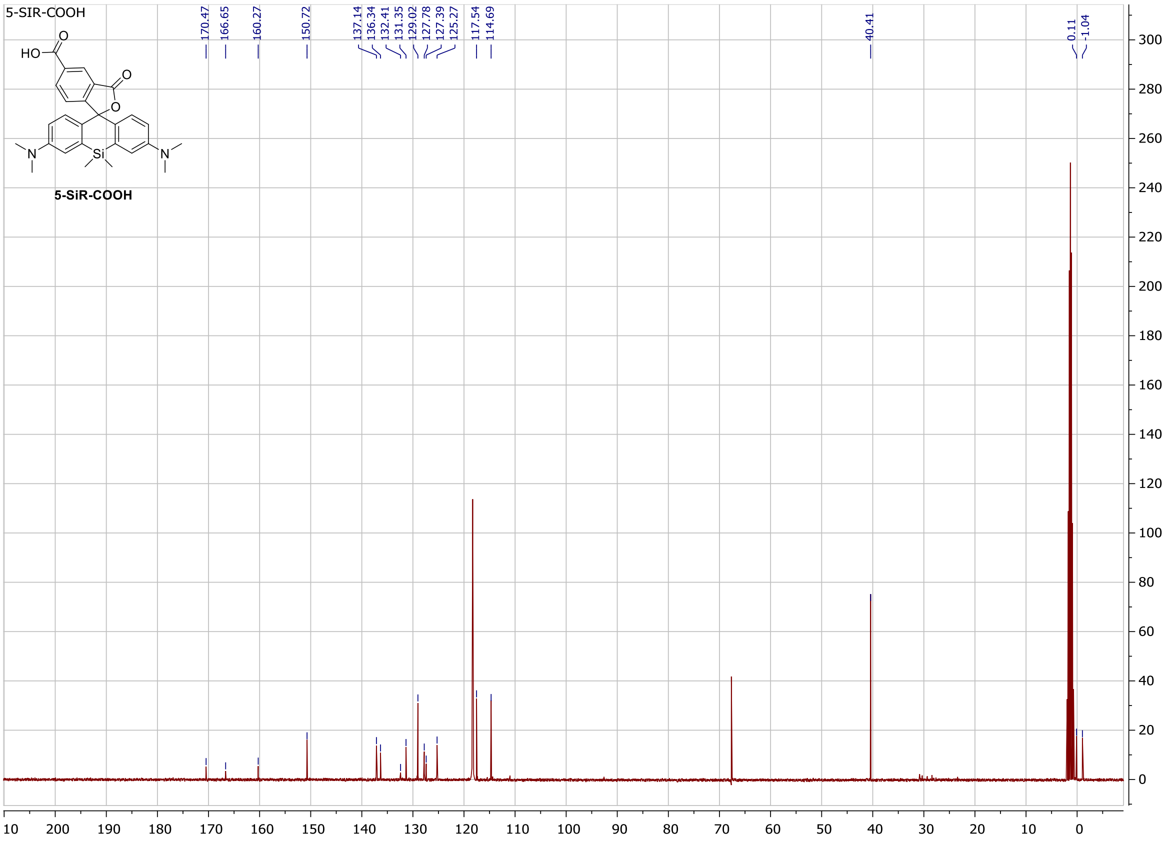
5-SIR-COOH
5-SiR-COOH
170.47 166.65 160.27 150.72 137.14 136.34 132.41 131.35 129.02 127.78 127.39 125.27 117.54 114.69
40.41
0.11 -1.04

2,2'-(4-bromo-1,3-phenylene)bis(4,4-dimethyl-4,5-dihydro-1,3-oxazole) SI-1

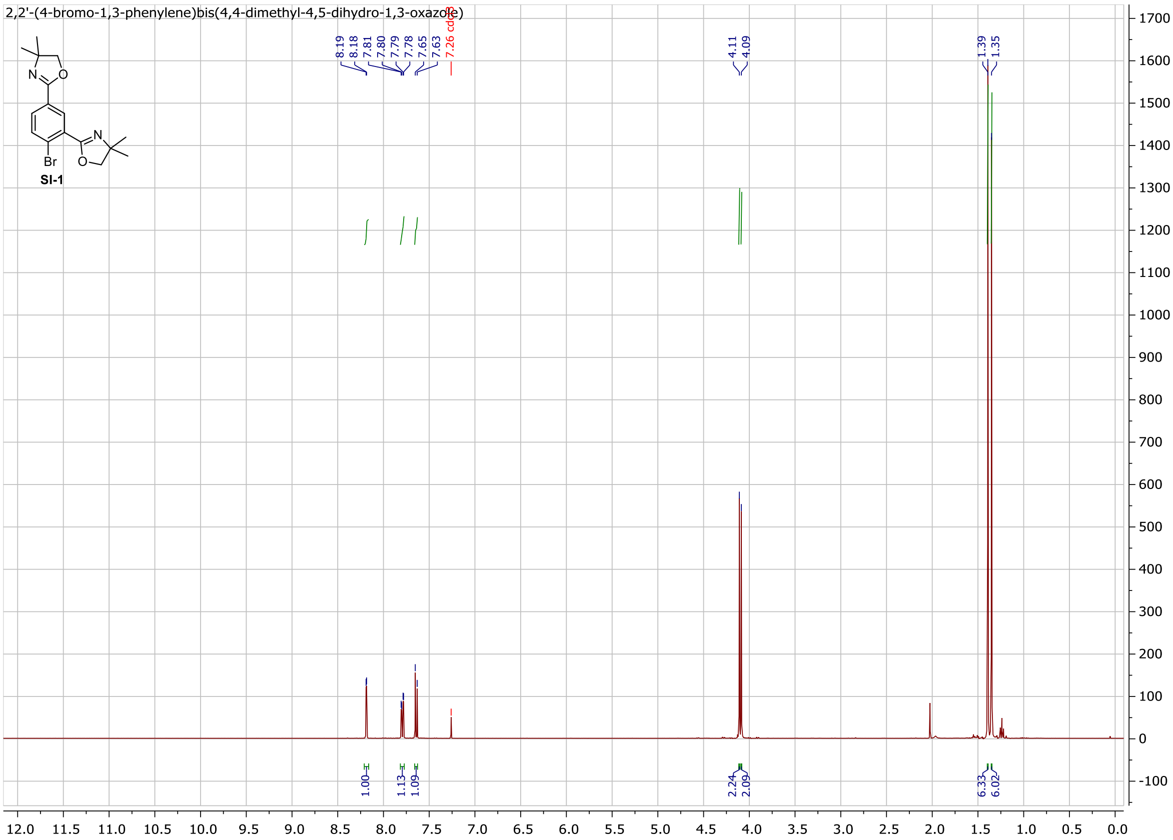

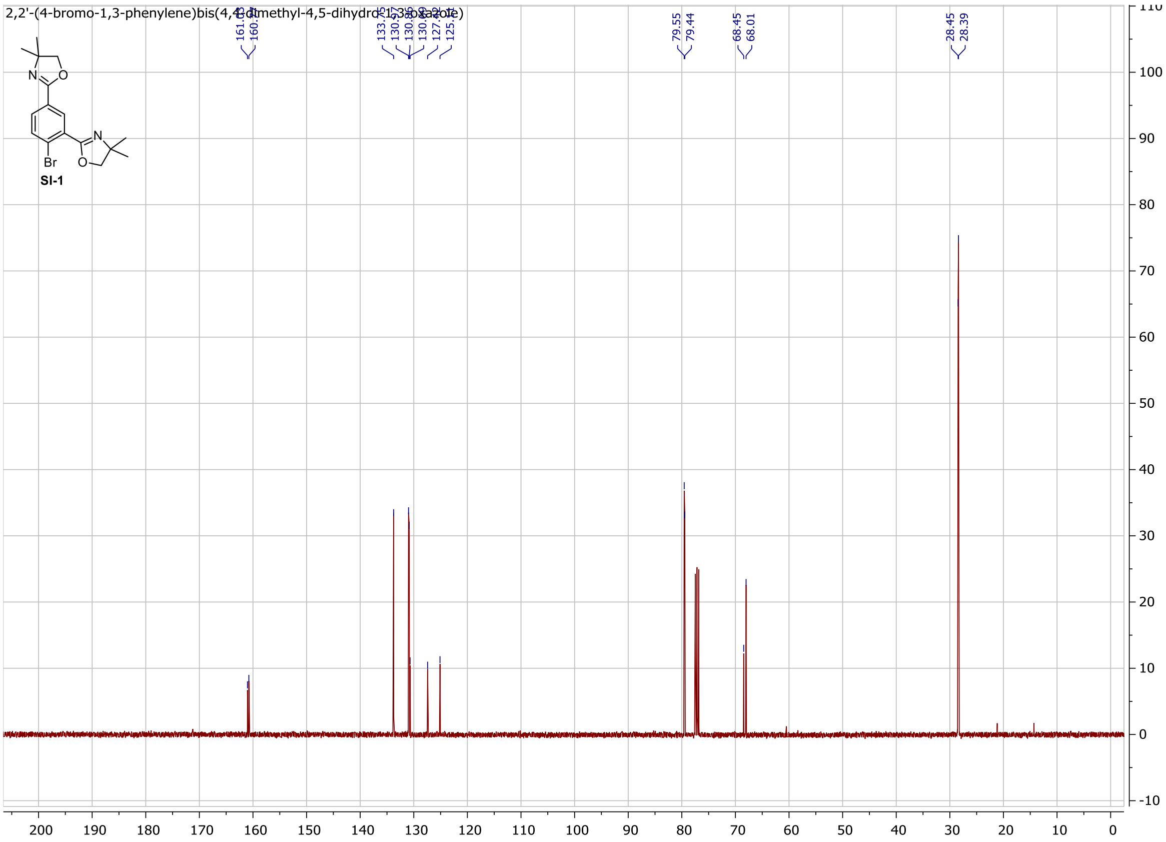

Di-tert-butyl 4-bromoisophtalate (SI-4)

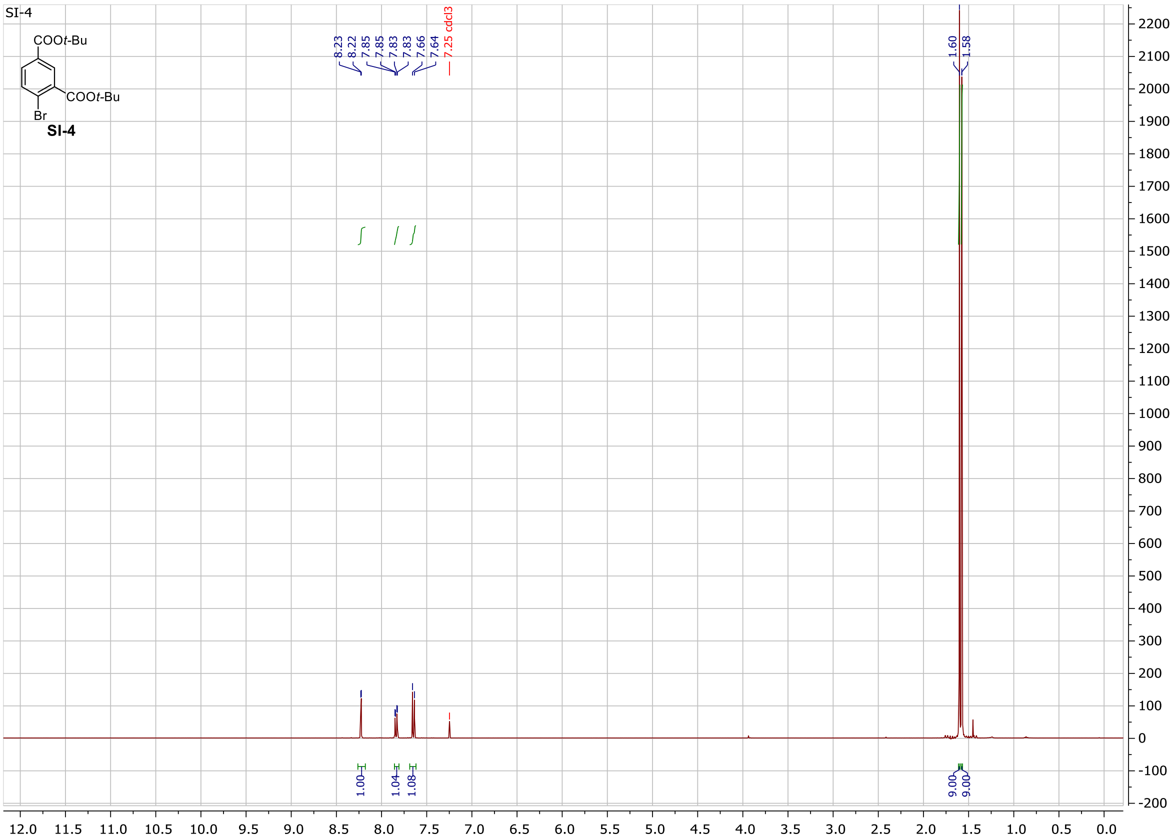

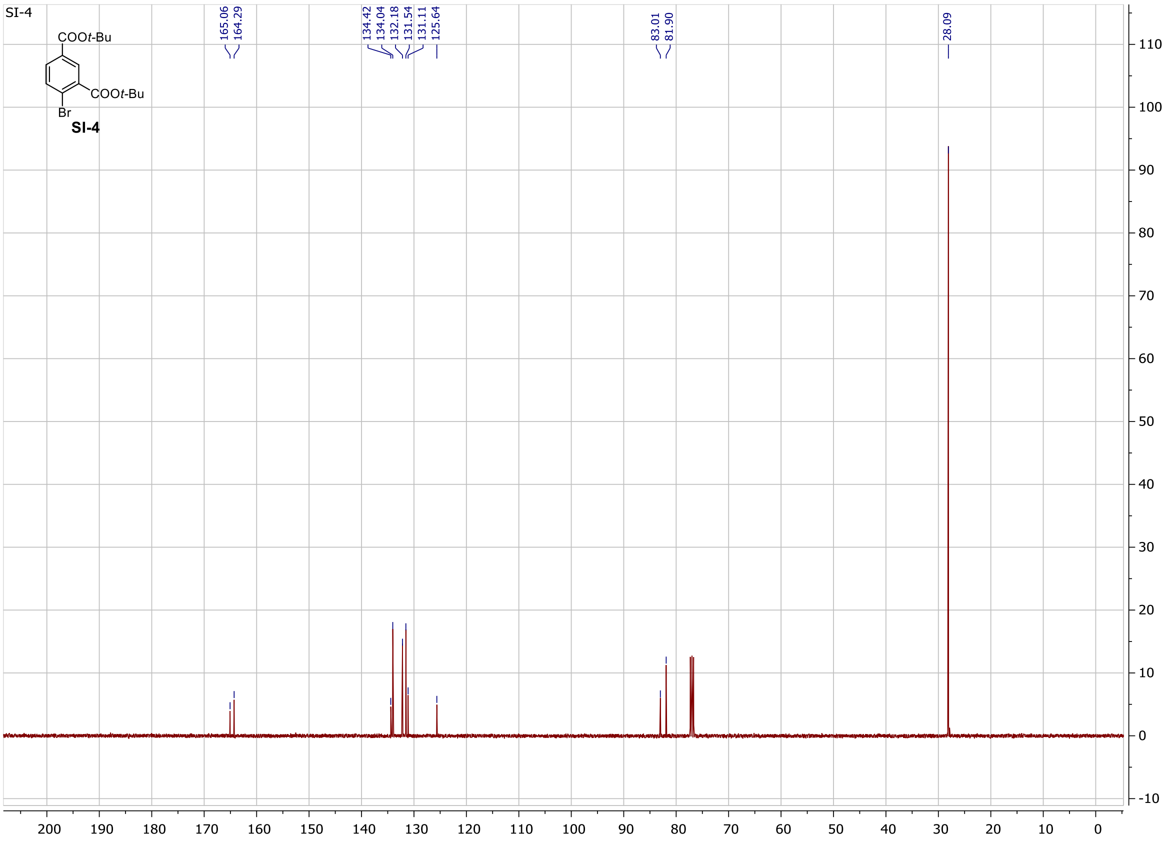

Tert-butyl 3,6-bis{[tert-butyl(dimethyl)silyl]oxy}-10,10-dimethyl-3'-oxo-3'H,10H-spiro[anthracene-9,1'-[2]benzofuran]-5'-carboxylate (SI-6)

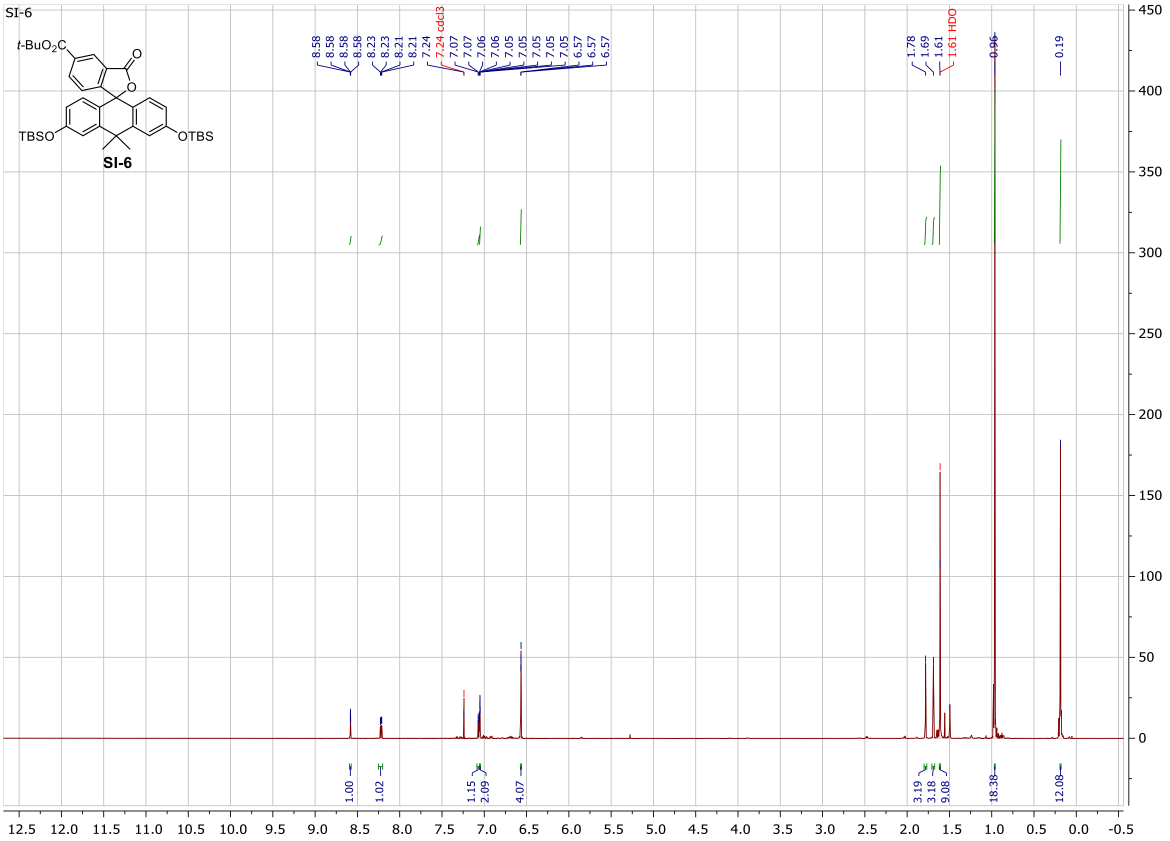

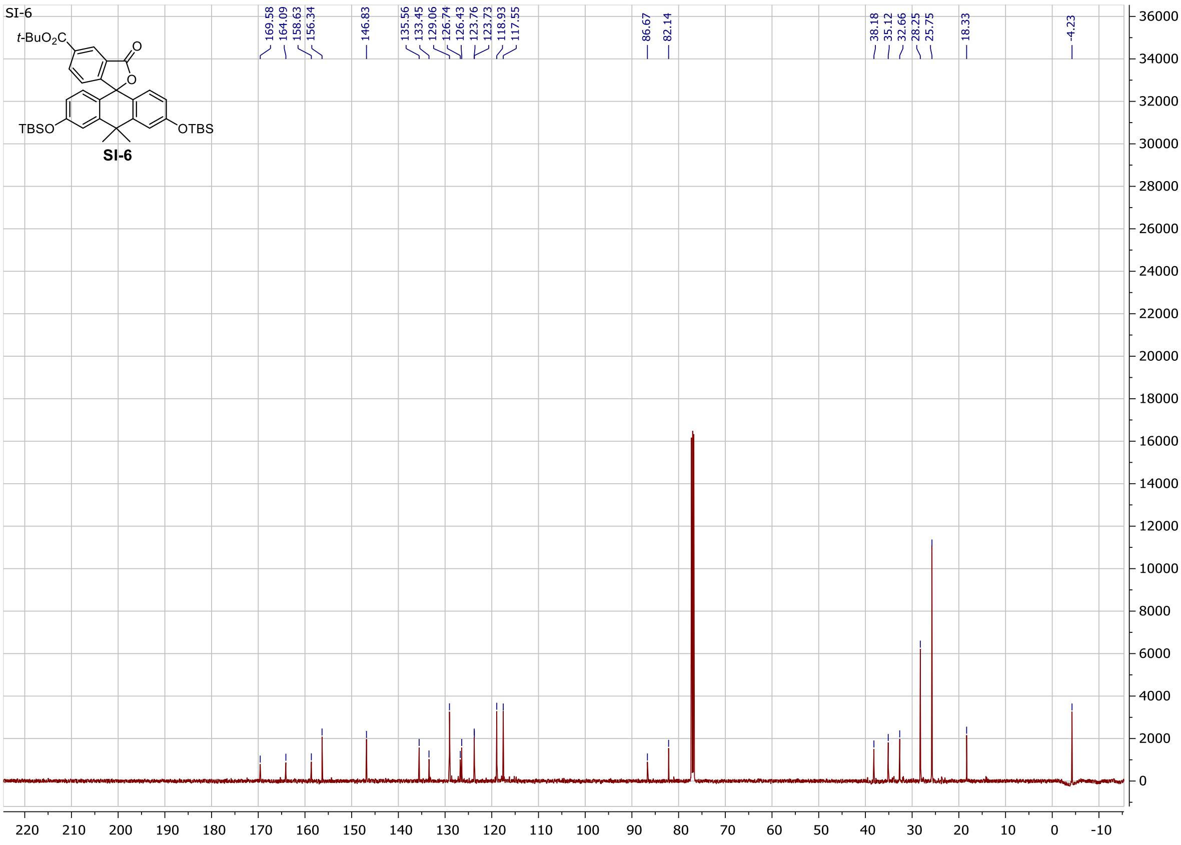

Tert-butyl 3,6-dihydroxy-10,10-dimethyl-3'-oxo-3'H,10H-spiro[anthracene-9,1'-[2]benzofuran]-5'-carboxylate (SI-7)

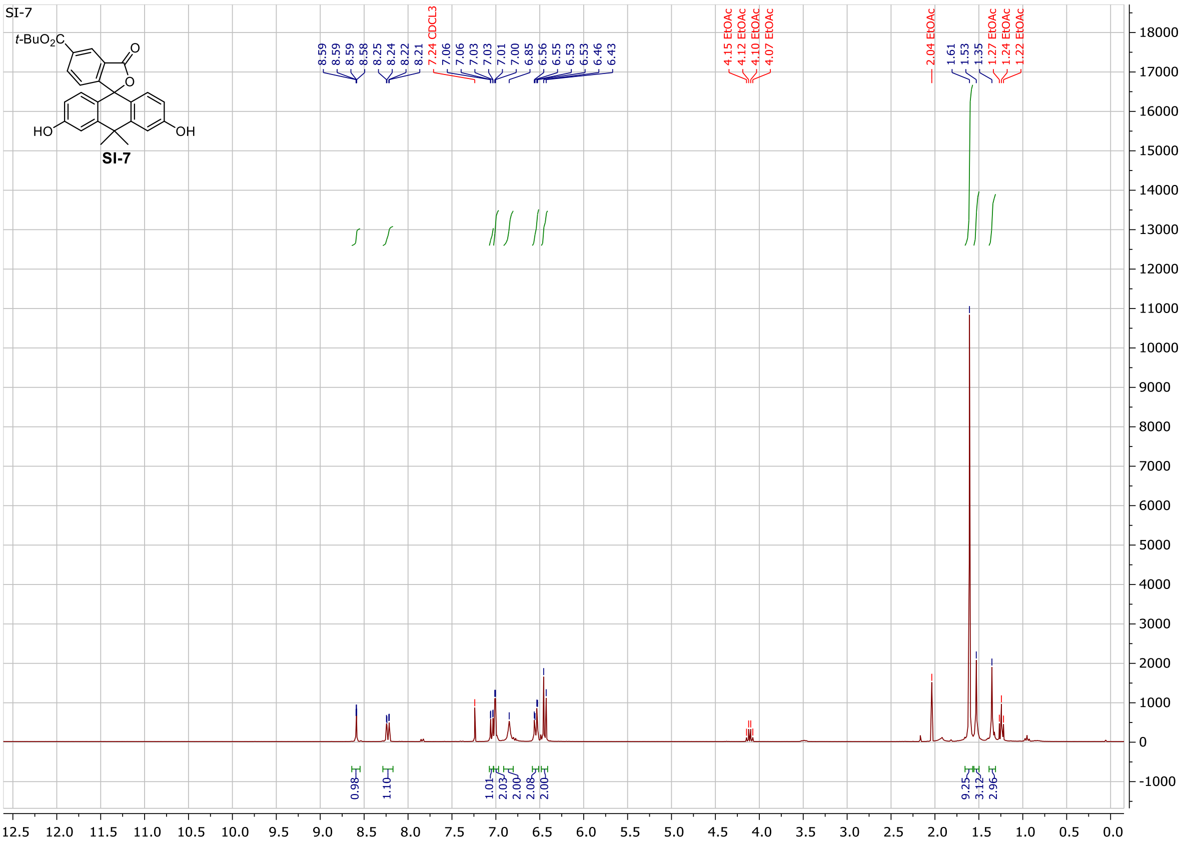

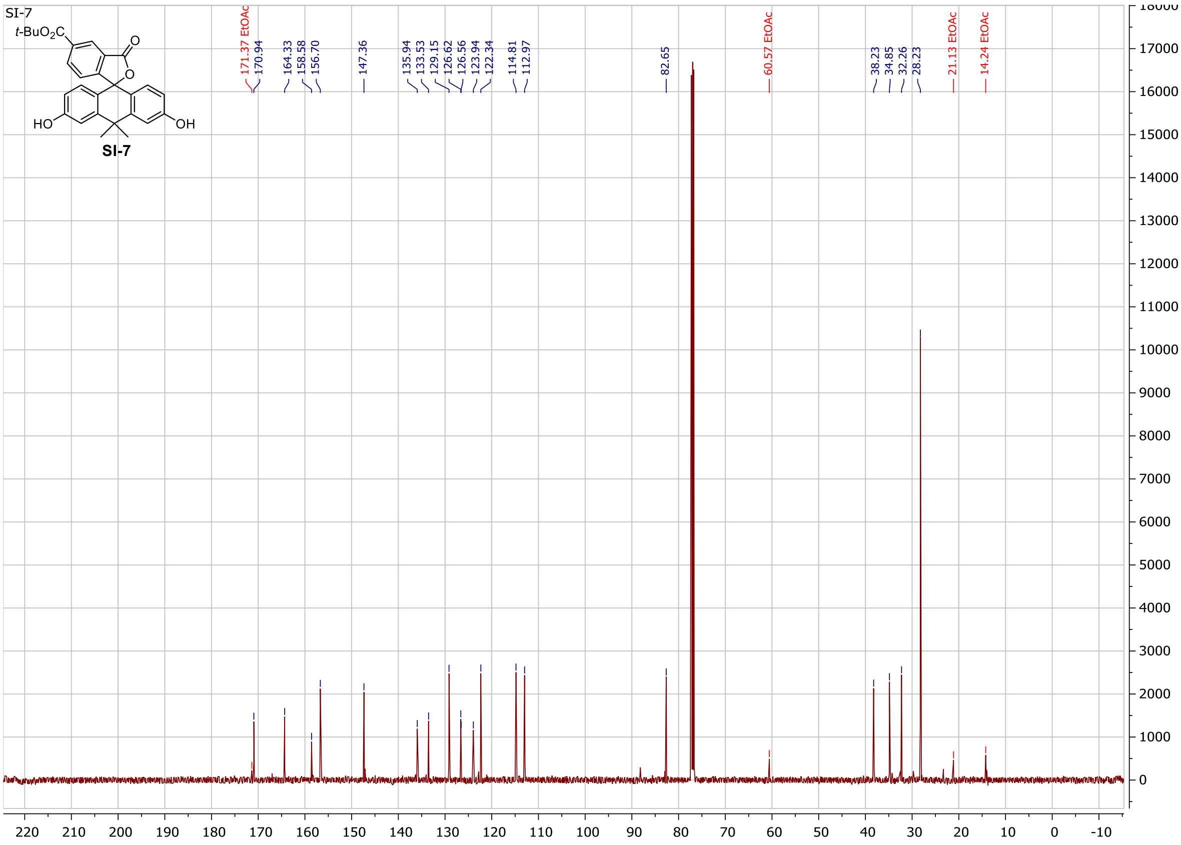

5-GeR-COOH

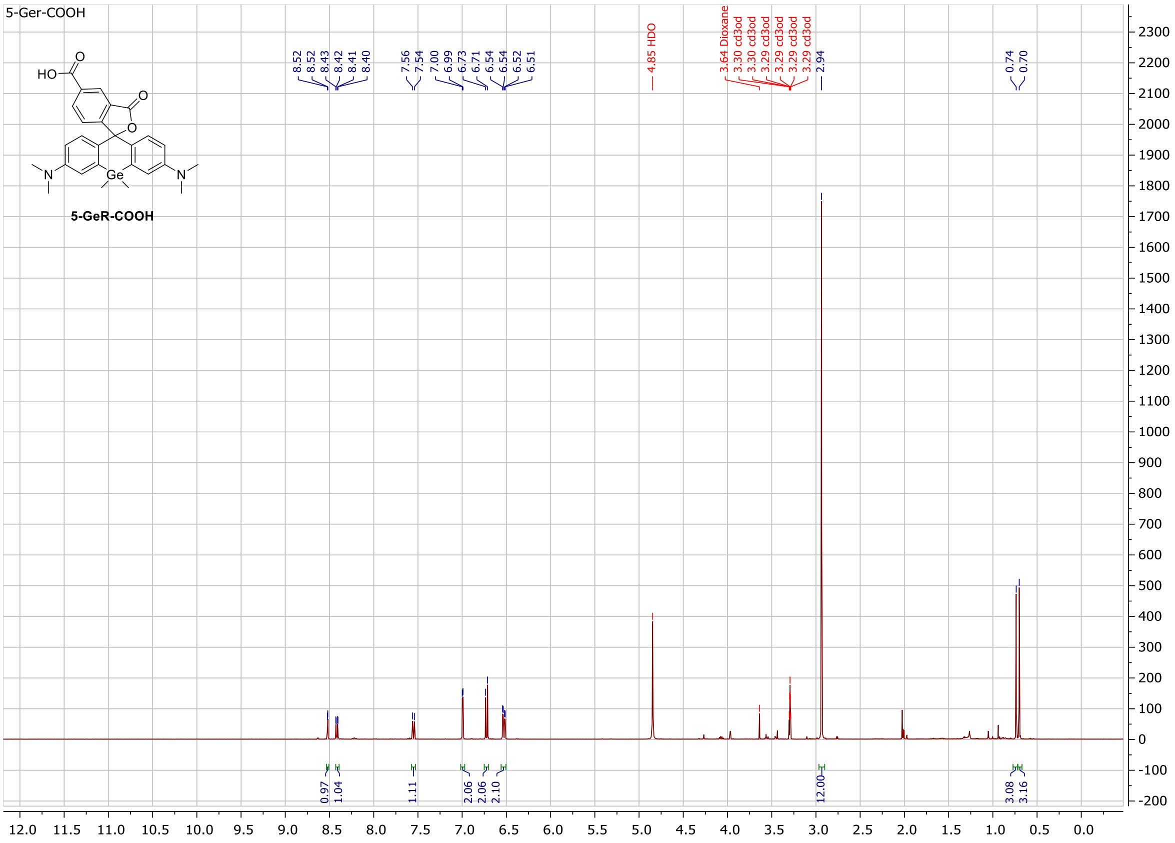
5-Ger-COOH
5-GeR-COOH
8.52 8.52 8.43 8.42 8.41 8.40
7.56 7.54
7.00 6.99 6.73 6.71 6.54 6.54 6.52 6.51
4.85 HDO
3.64 Dioxane
3.30 cd3od 3.30 cd3od 3.29 cd3od 3.29 cd3od 3.29 cd3od 3.29 cd3od
2.94
0.74 0.70
0.97 1.04 1.11 2.06 2.06 2.10 12.00 3.08 3.16

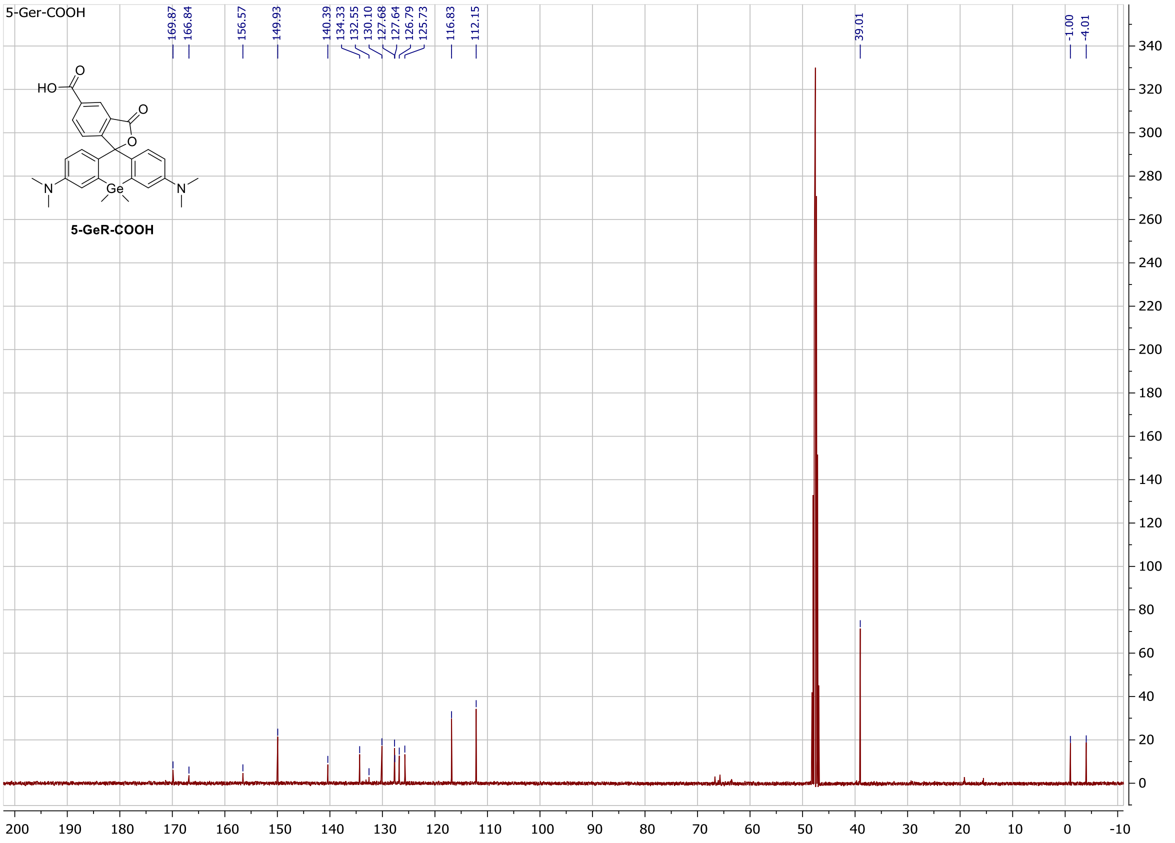
5-Ger-COOH
5-GeR-COOH
169.87 166.84 156.57 149.93 140.39 134.33 132.55 130.10 127.68 127.64 126.79 125.73 116.83 112.15
39.01
-1.00 -4.01

Tert-butyl 10,10-dimethyl-3'-oxo-3,6-bis[(trifluoromethanesulfonyl)oxy]-3'H,10H-spiro[anthracene-9,1'-[2]benzofuran]-5'-carboxylate (SI-8)

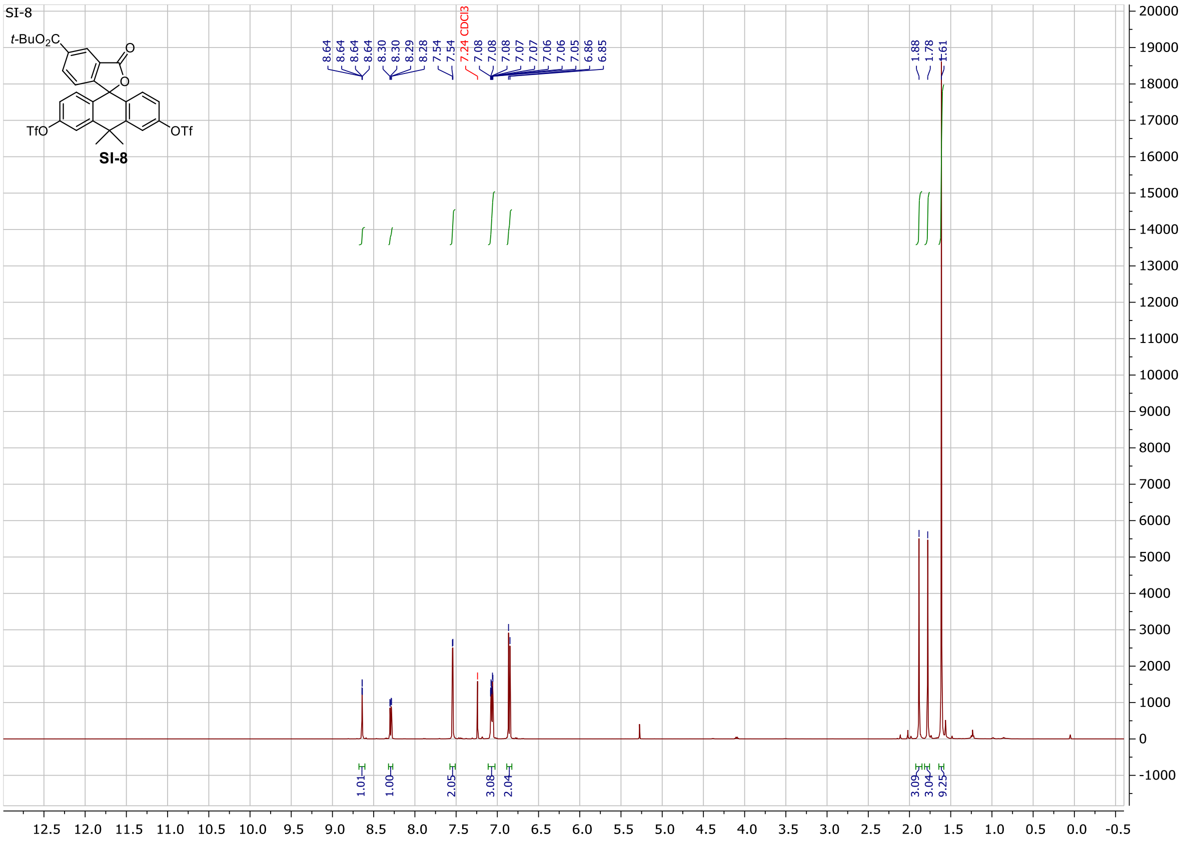

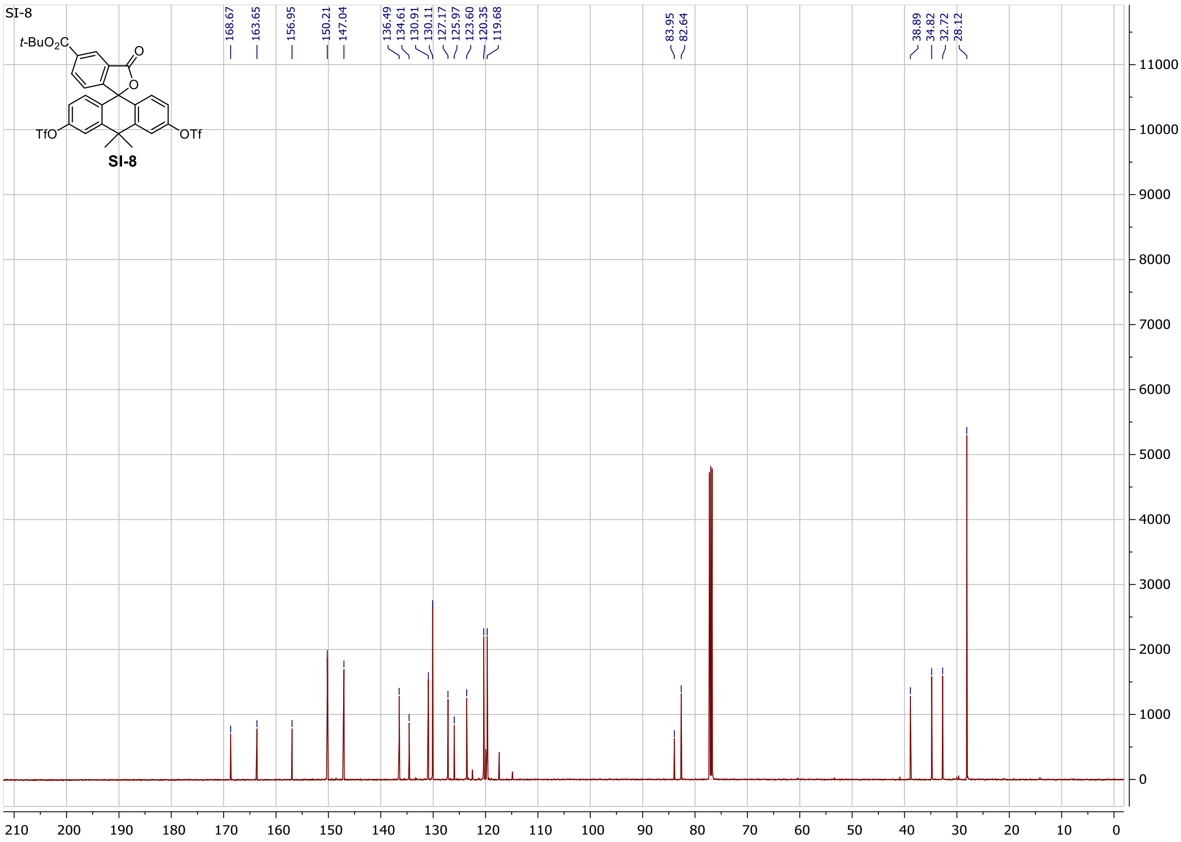

Tert-butyl 3,6-bis[(tert-butoxycarbonyl)(methyl)amino]-10,10-dimethyl-3'-oxo-3'H,10H-spiro[anthracene-9,1'-[2]benzofuran]-5'-carboxylate SI-9

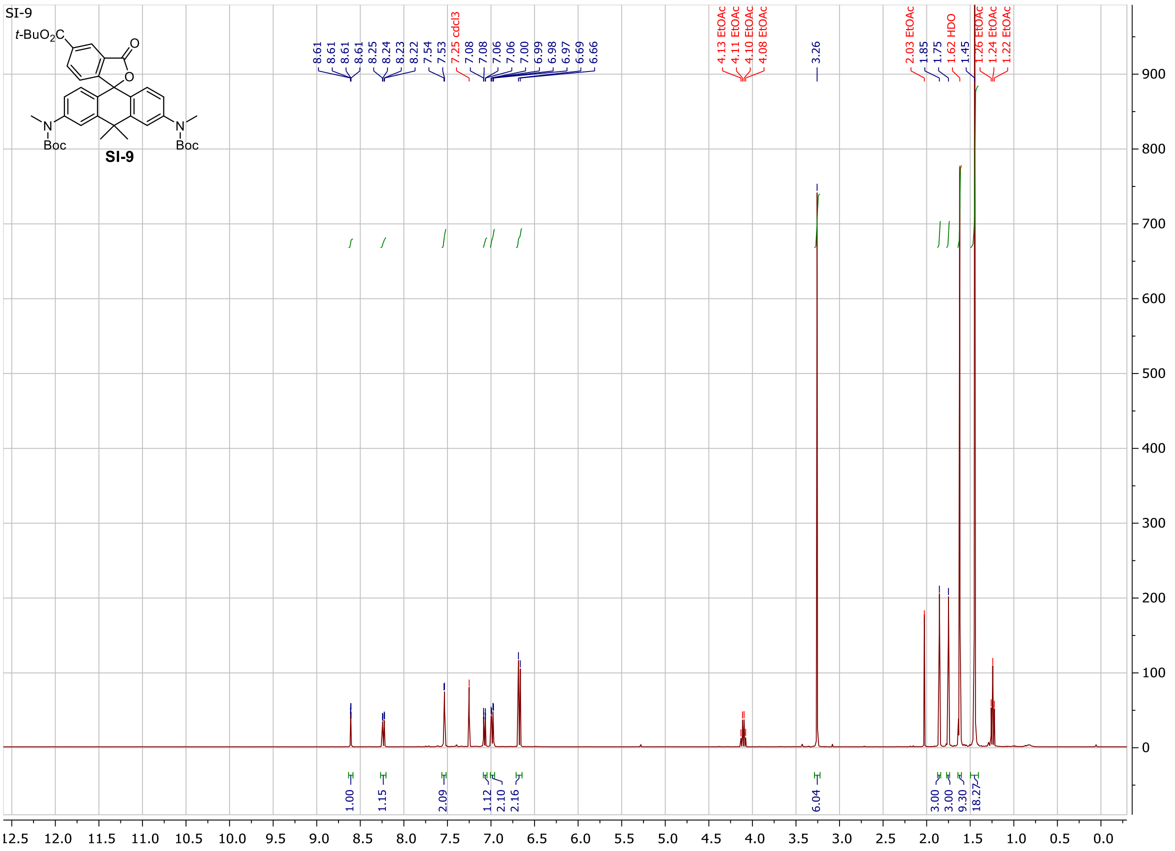

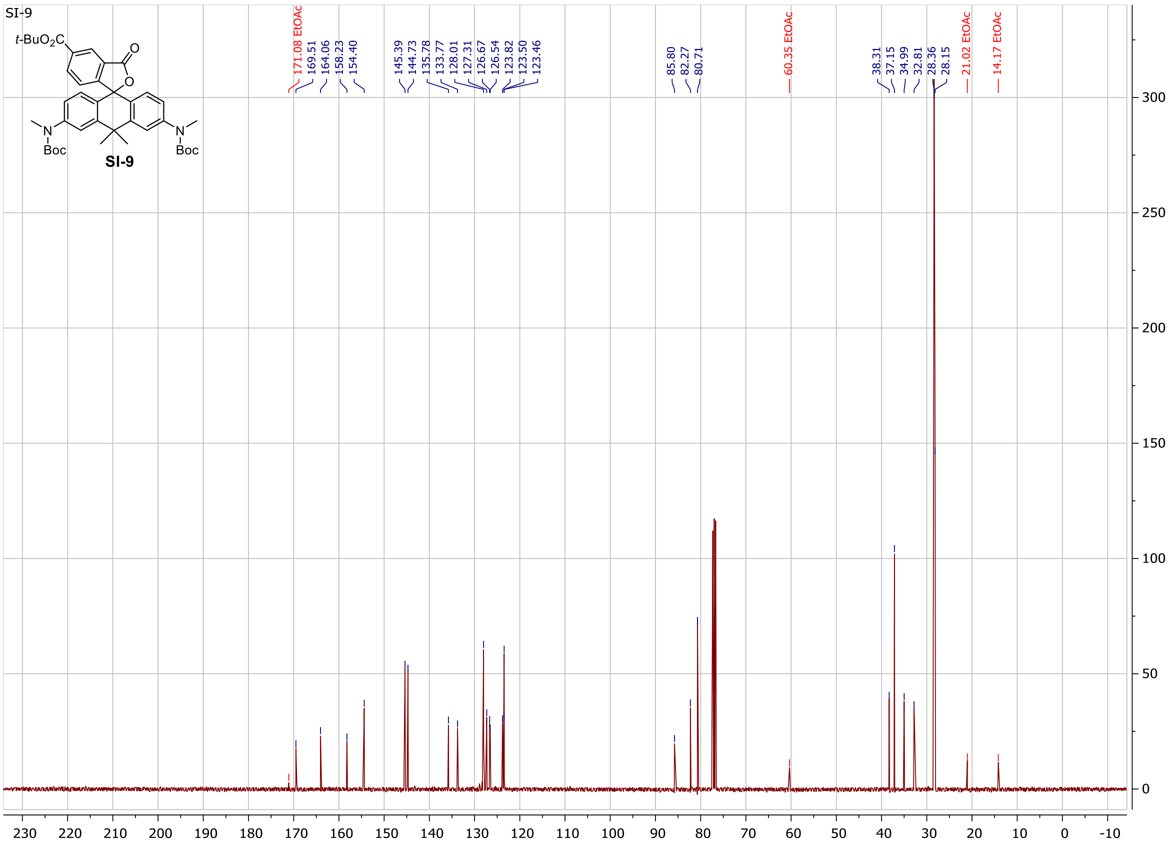

Tert-butyl (3-{4-[5-(4-methylpiperazin-1-yl)-1H,1'H-[2,5'-bibenzimidazol]-2'-yl]phenoxy}butyl)carbamate (SI-12)

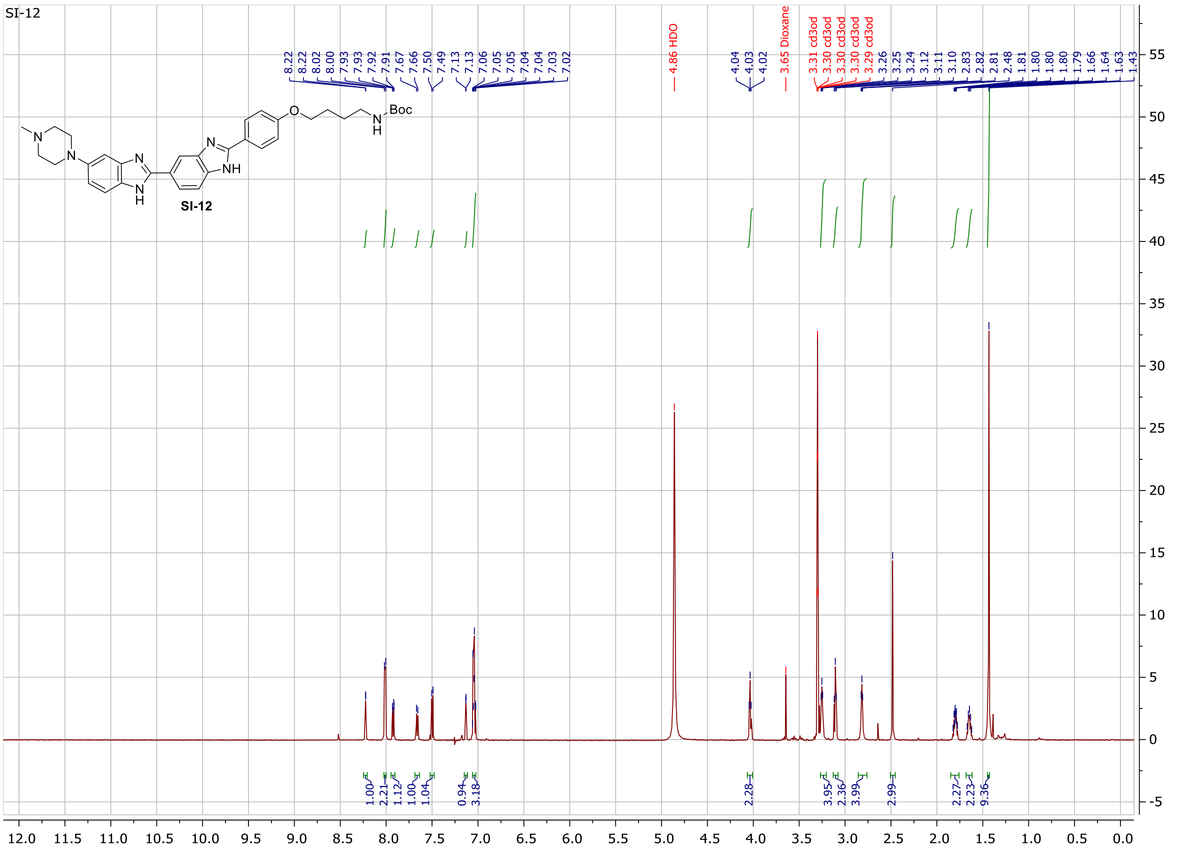

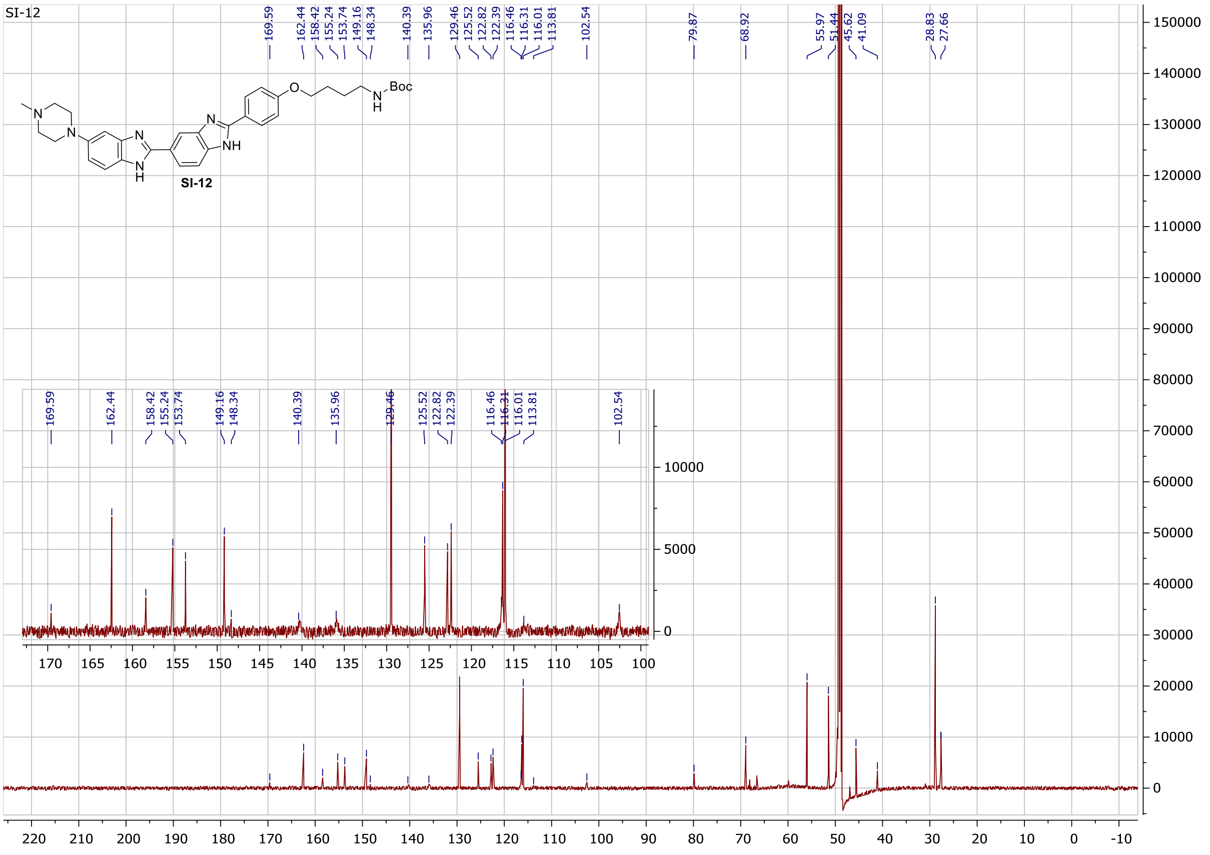

4-{4-[5-(4-methylpiperazin-1-yl)-1*H*,1'*H*-[2,5'-bibenzimidazol]-2'-yl]phenoxy}butan-1-amine (SI-13)

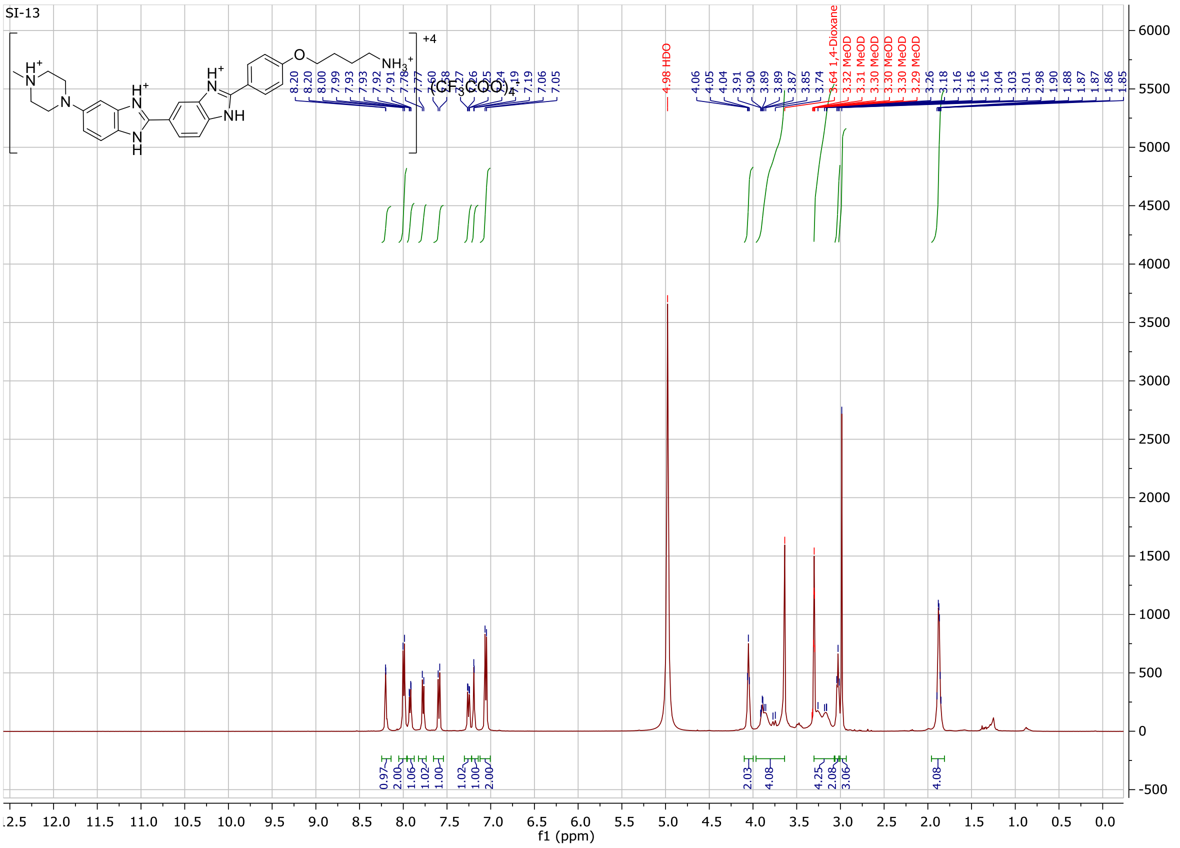

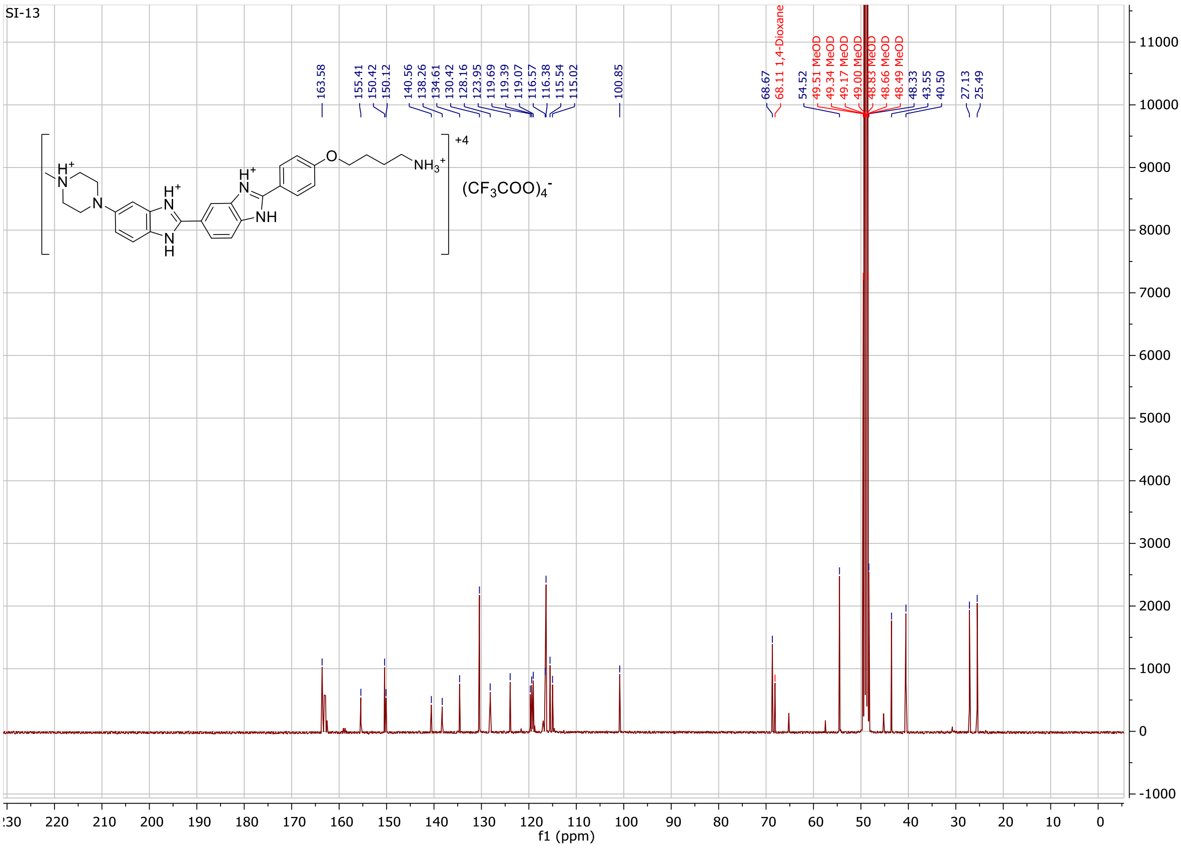

5-580CP-COOH

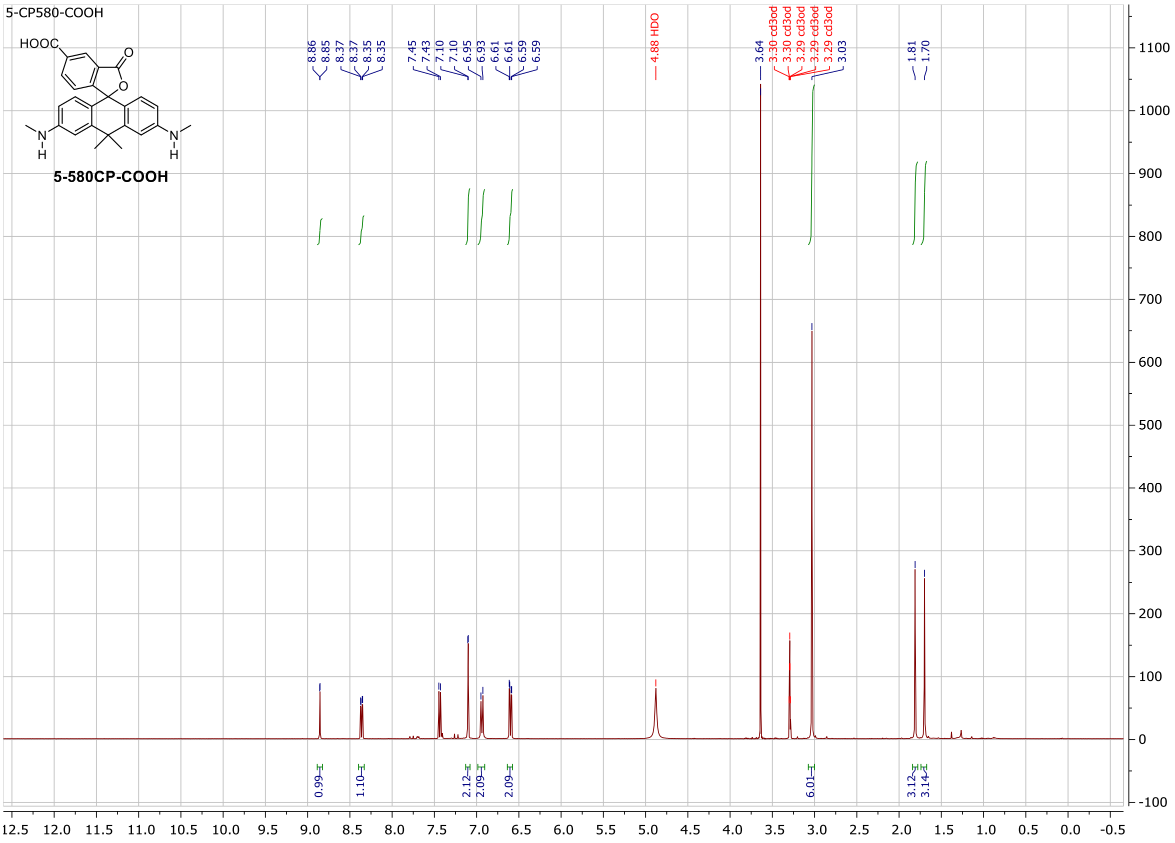
5-CP580-COOH
HOOC
O
O
N
H
N
H
5-580CP-COOH
8.86
8.85
8.37
8.37
8.35
8.35
7.45
7.43
7.10
7.10
6.95
6.93
6.61
6.61
6.59
6.59
4.88 HDO
3.64
3.30 cd3od
3.30 cd3od
3.29 cd3od
3.29 cd3od
3.29 cd3od
3.03
1.81
1.70
0.99
1.10
2.12
2.09
2.09
6.01
3.12
3.14

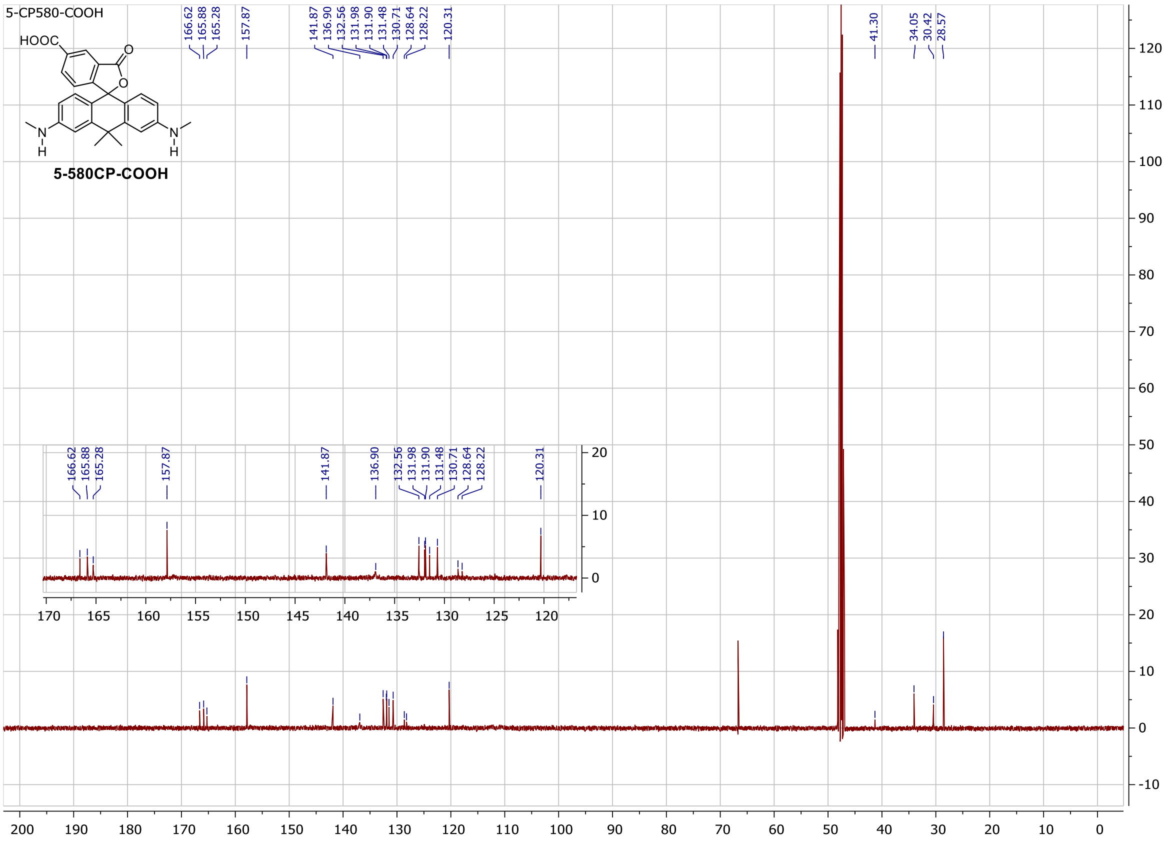
5-CP580-COOH
HOOC
O
O
N
H
N
H
5-580CP-COOH
166.62
165.88
165.28
157.87
141.87
136.90
132.56
131.98
131.90
131.48
130.71
128.64
128.22
120.31
41.30
34.05
30.42
28.57

6-580CP-Hoechst

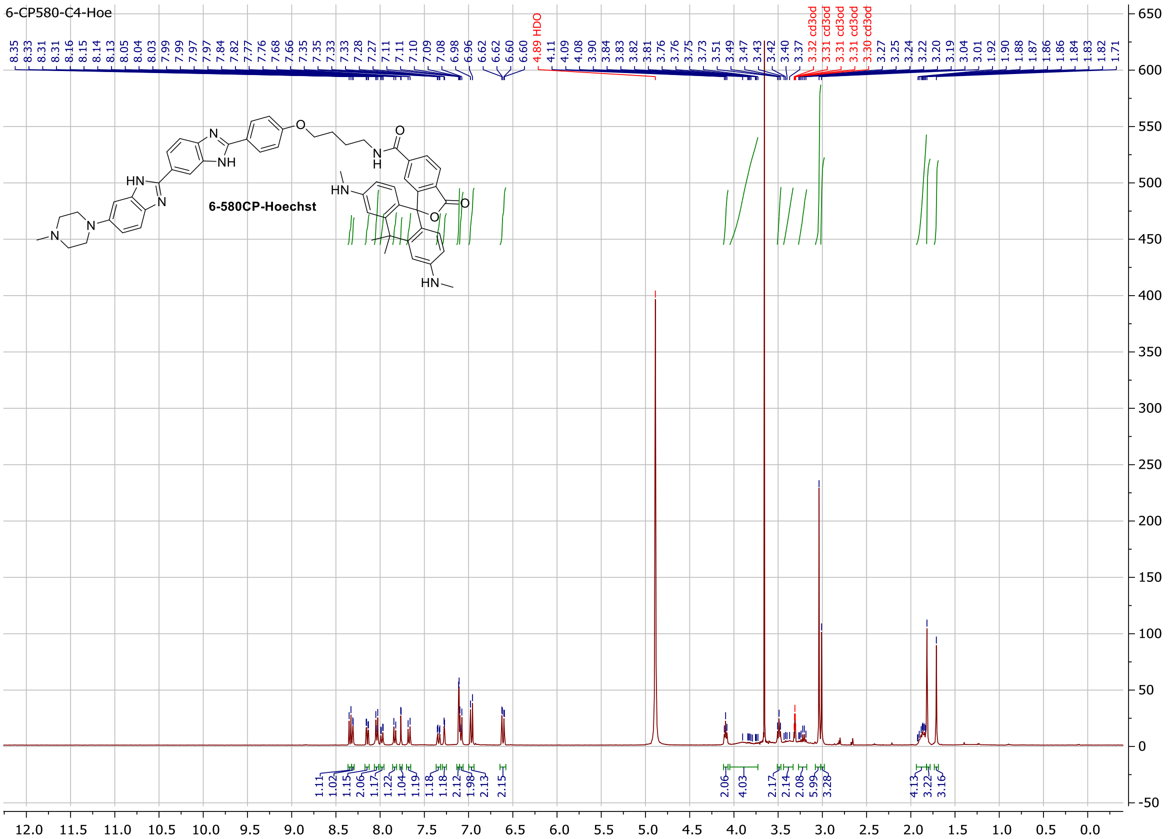

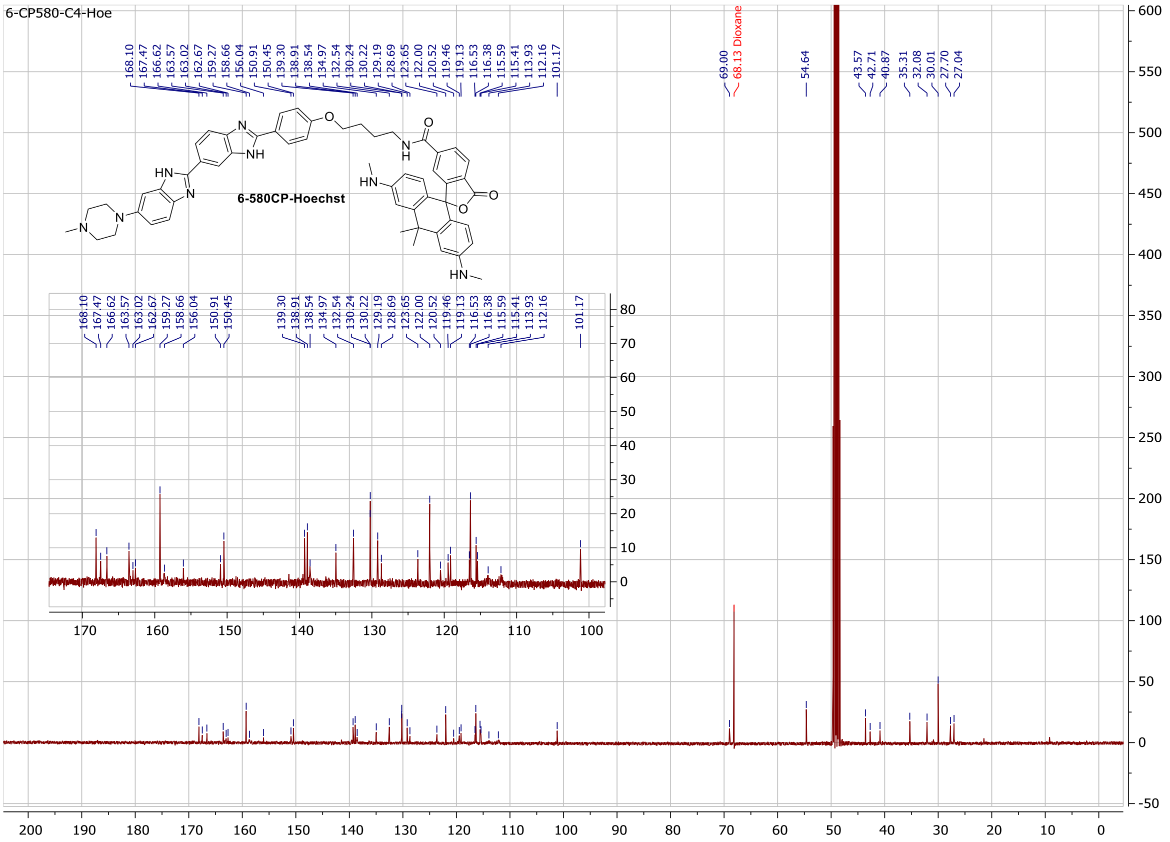

5-580CP-Hoechst

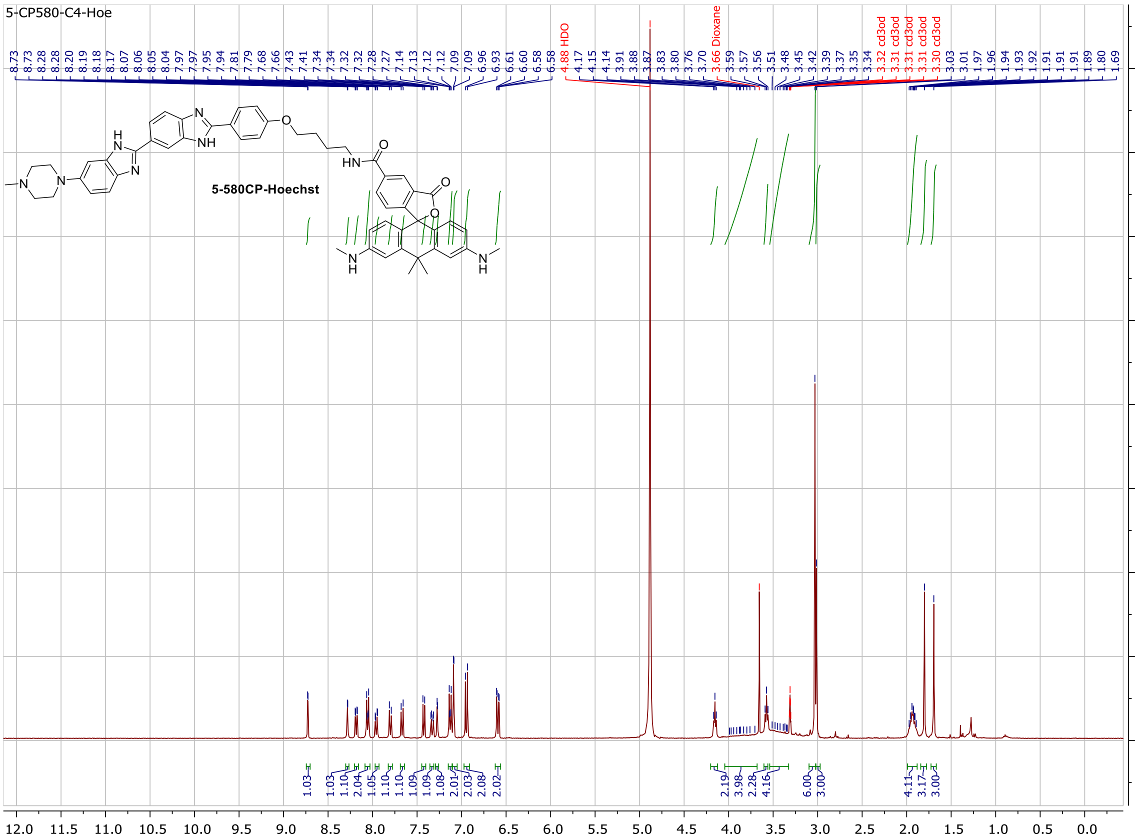

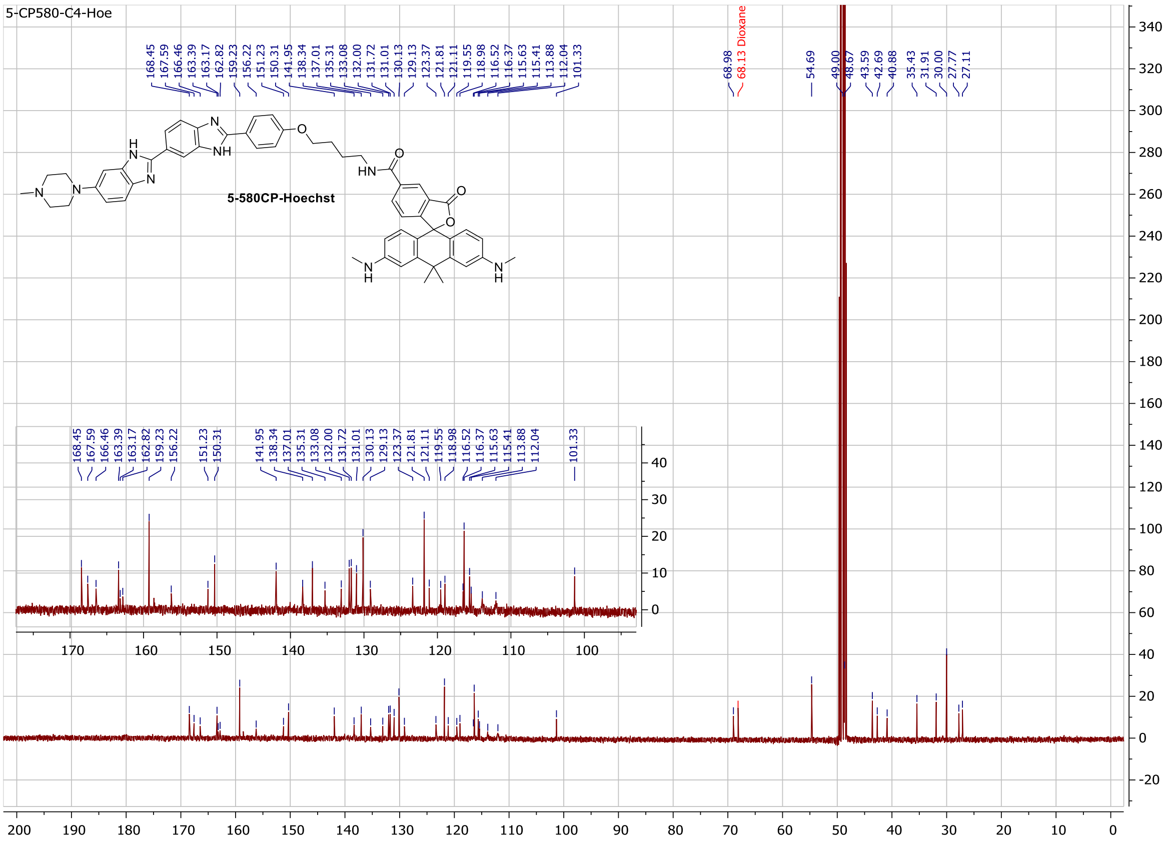

6-TMR-Hoechst

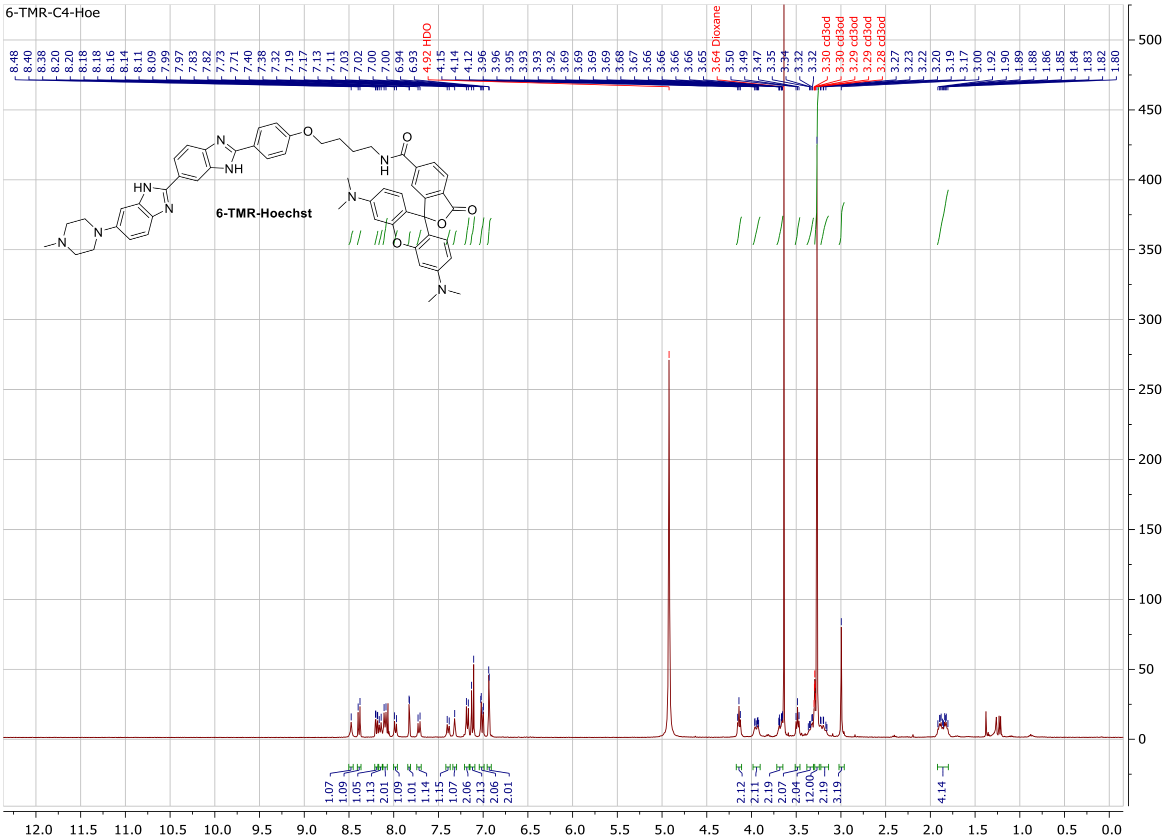

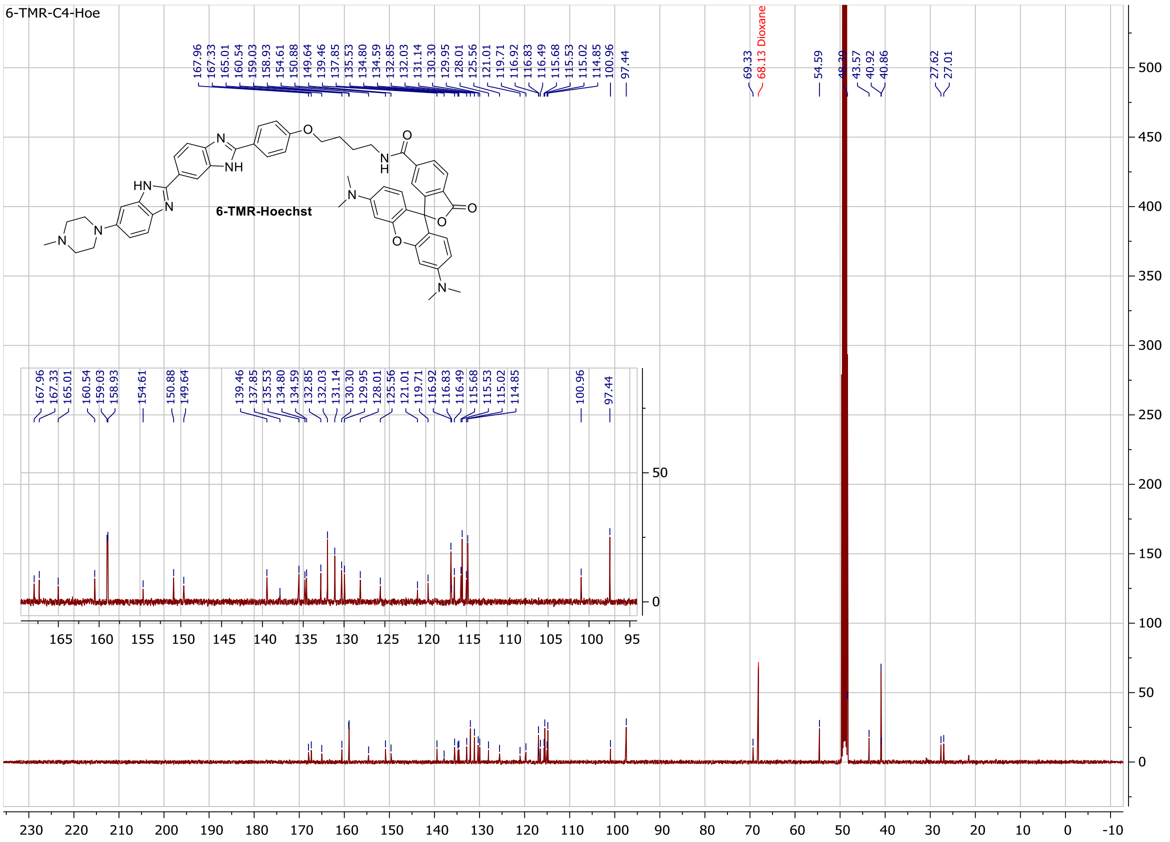

5-TMR-Hoechst

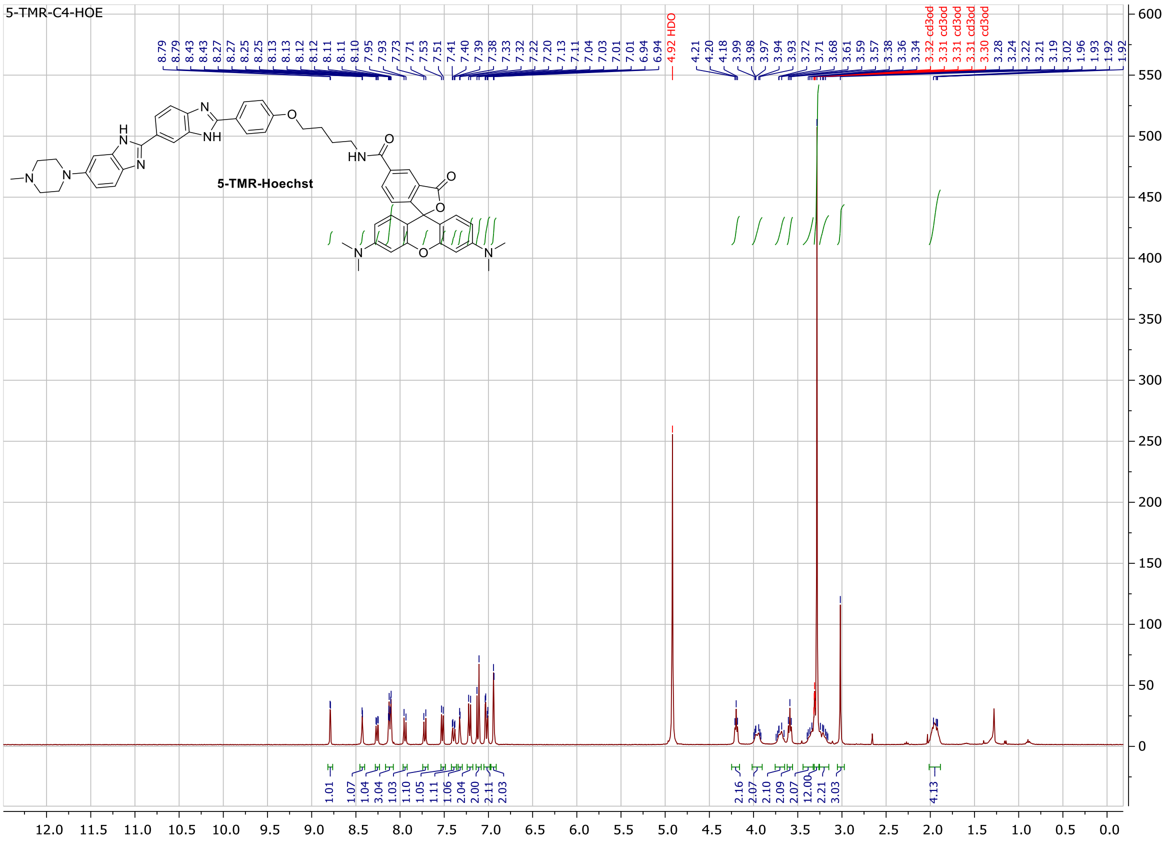

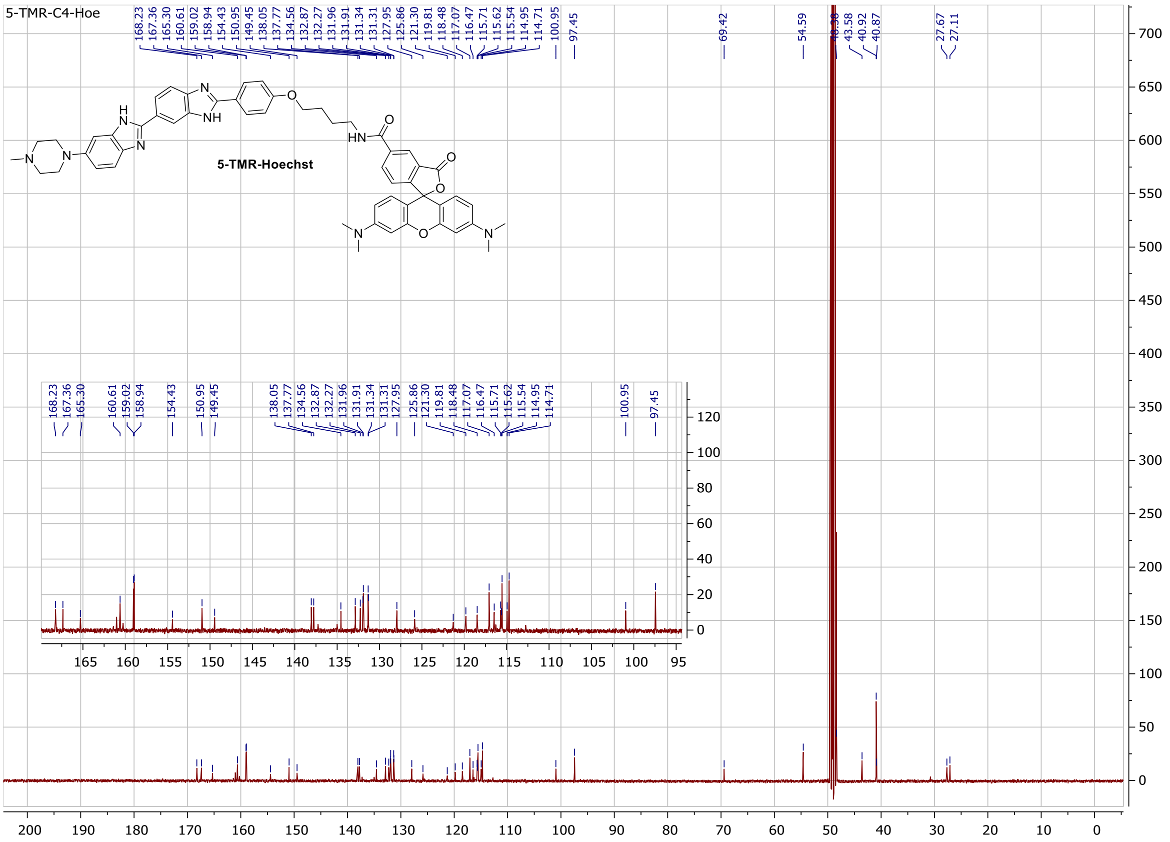

6-SiR-Hoechst

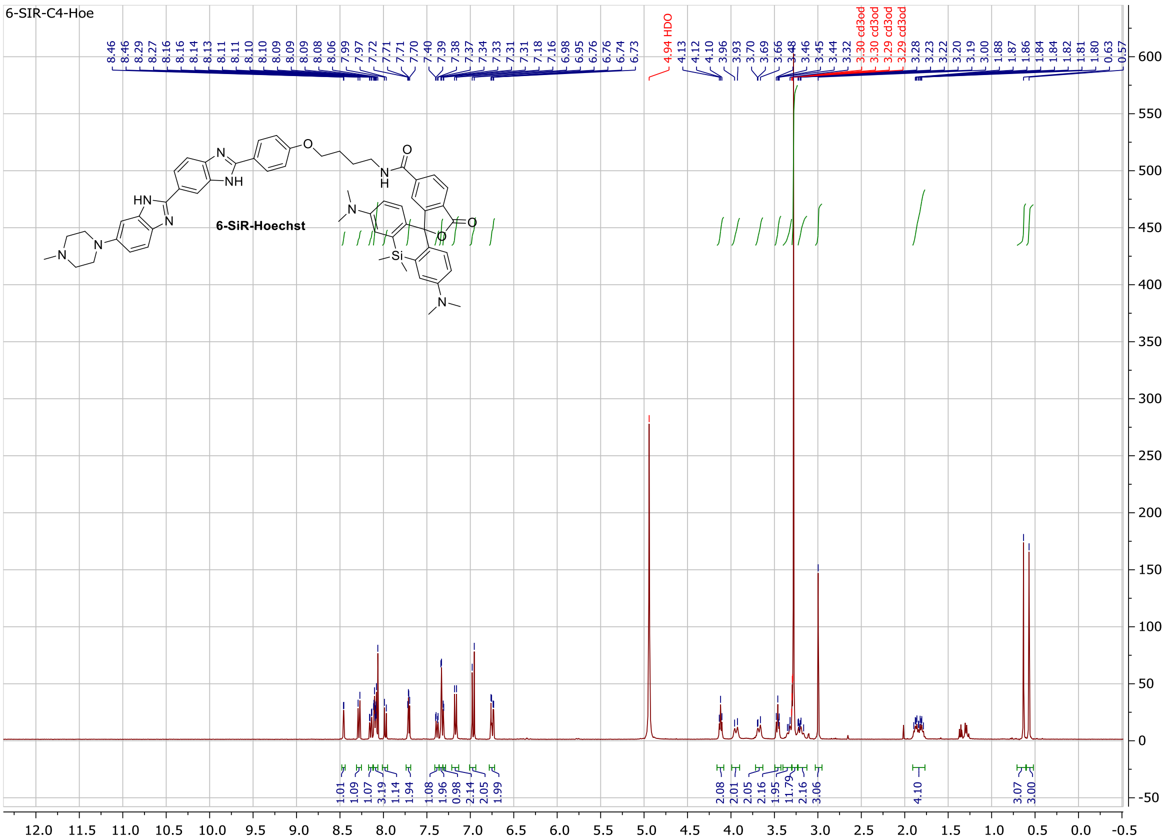

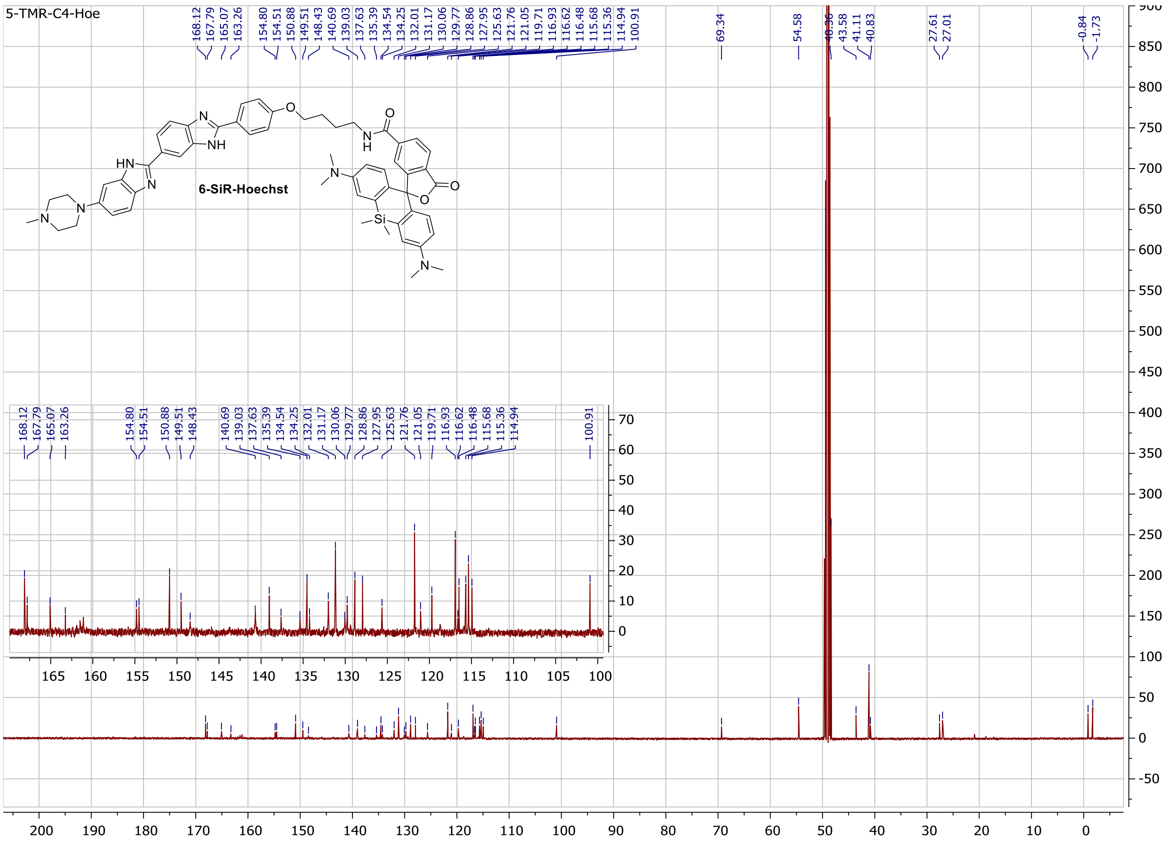

5-SiR-Hoechst

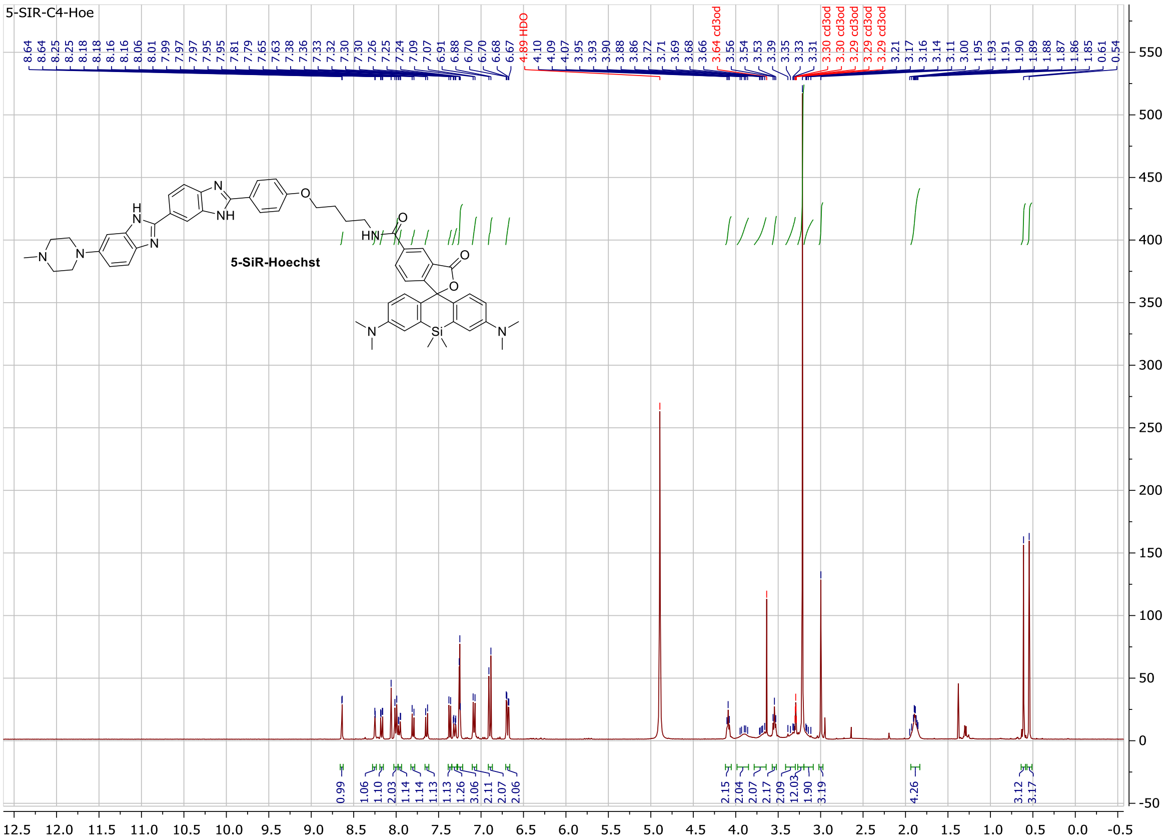

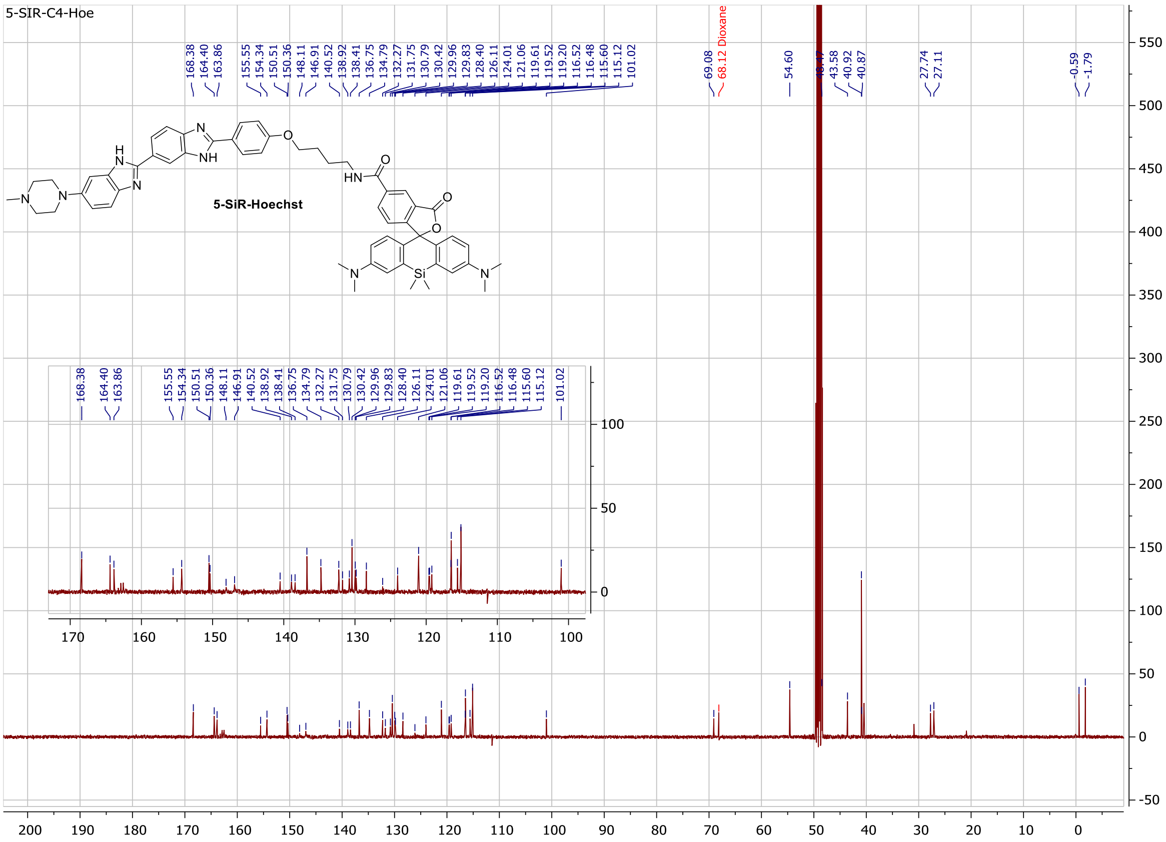

6-610CP-Hoechst

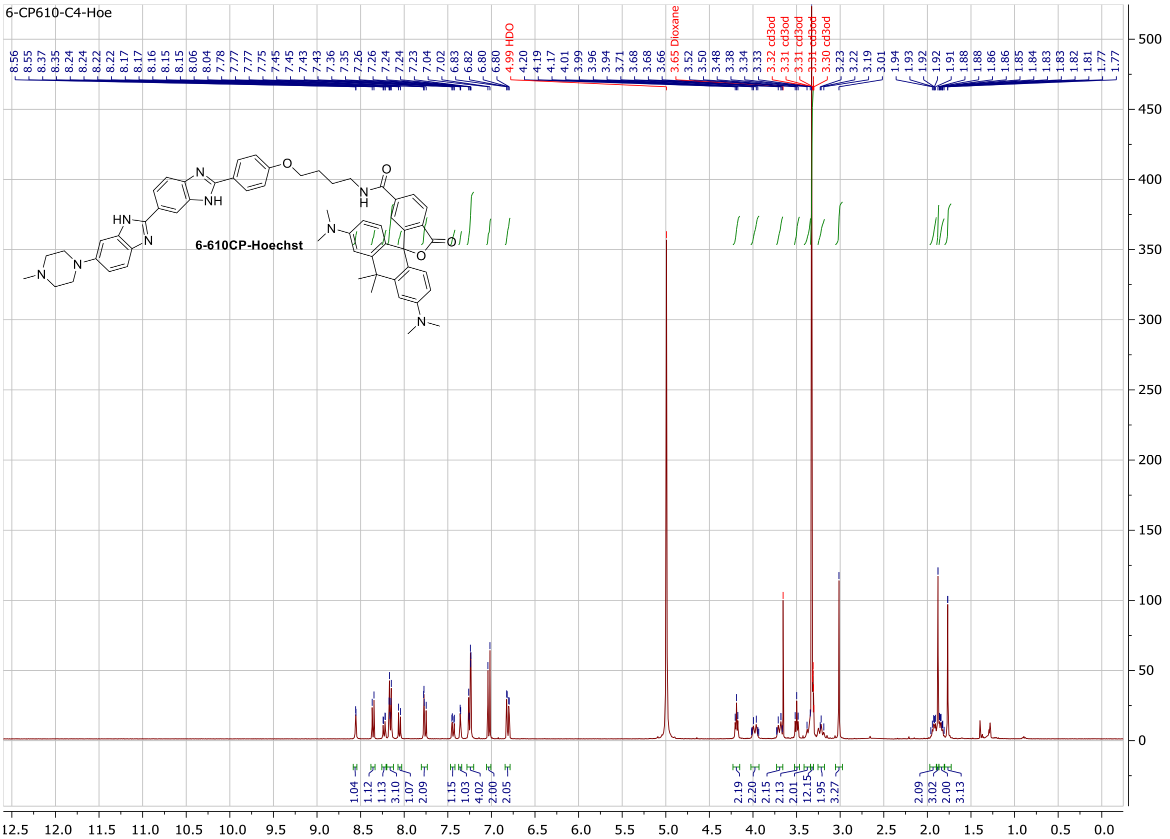

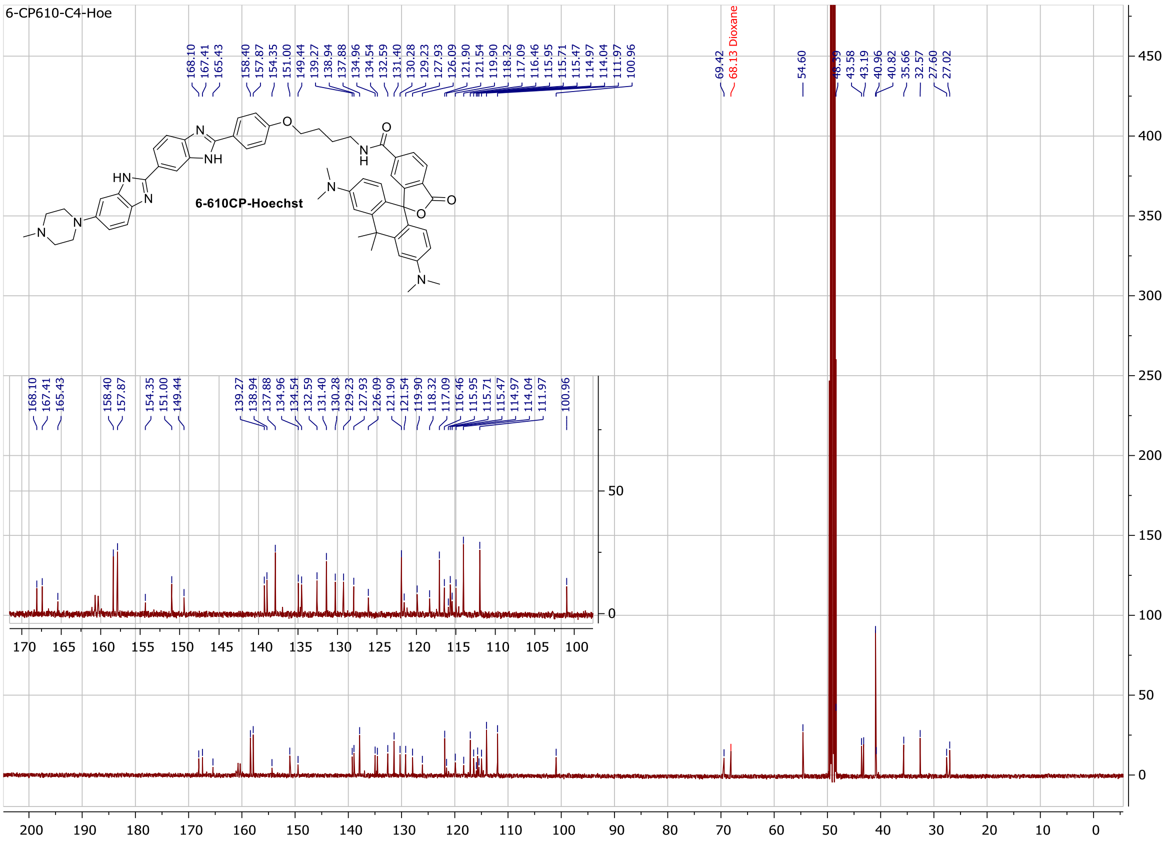

5-610CP-Hoechst

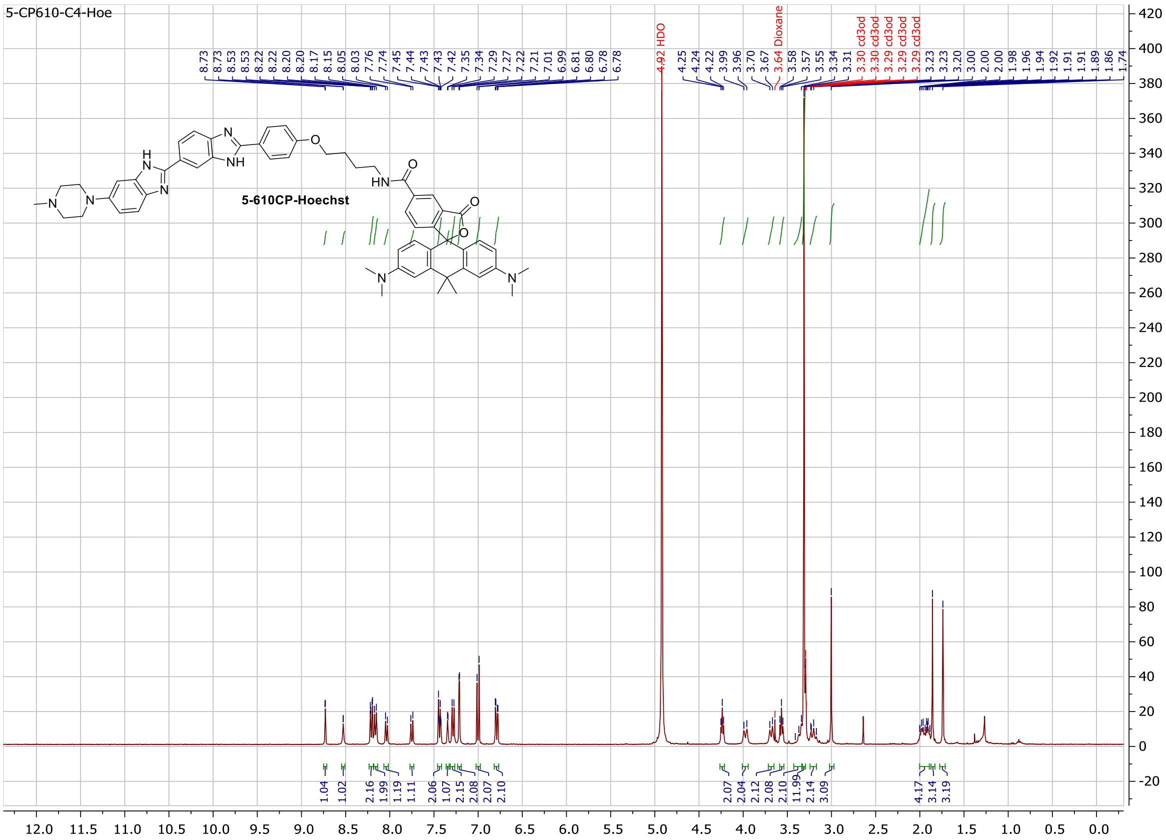

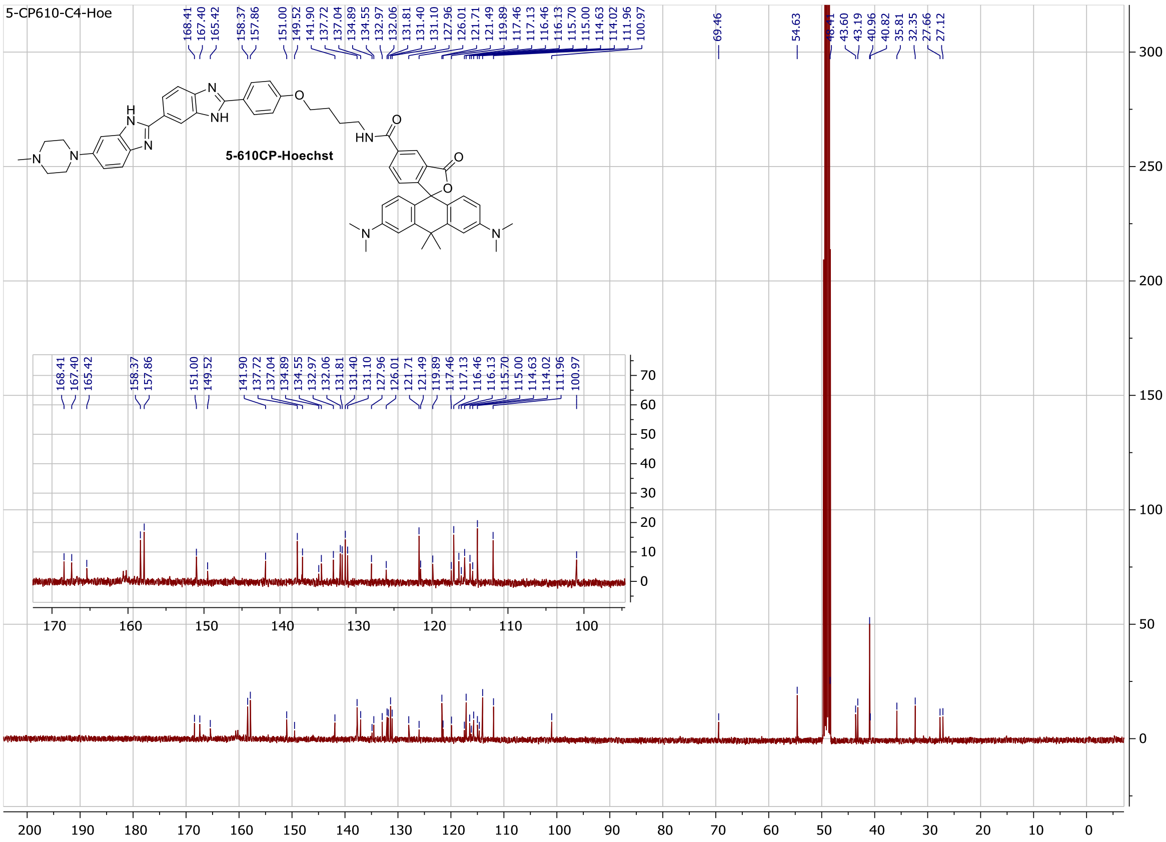

6-GeR-Hoechst

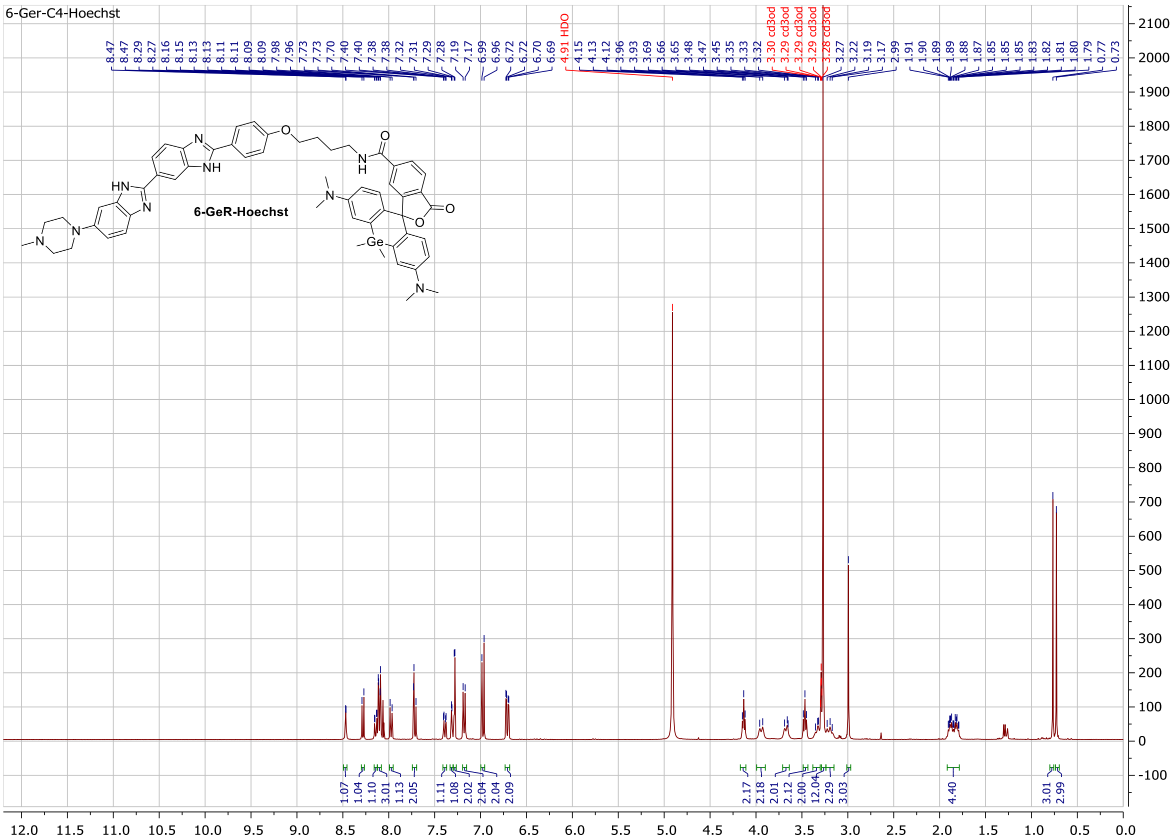

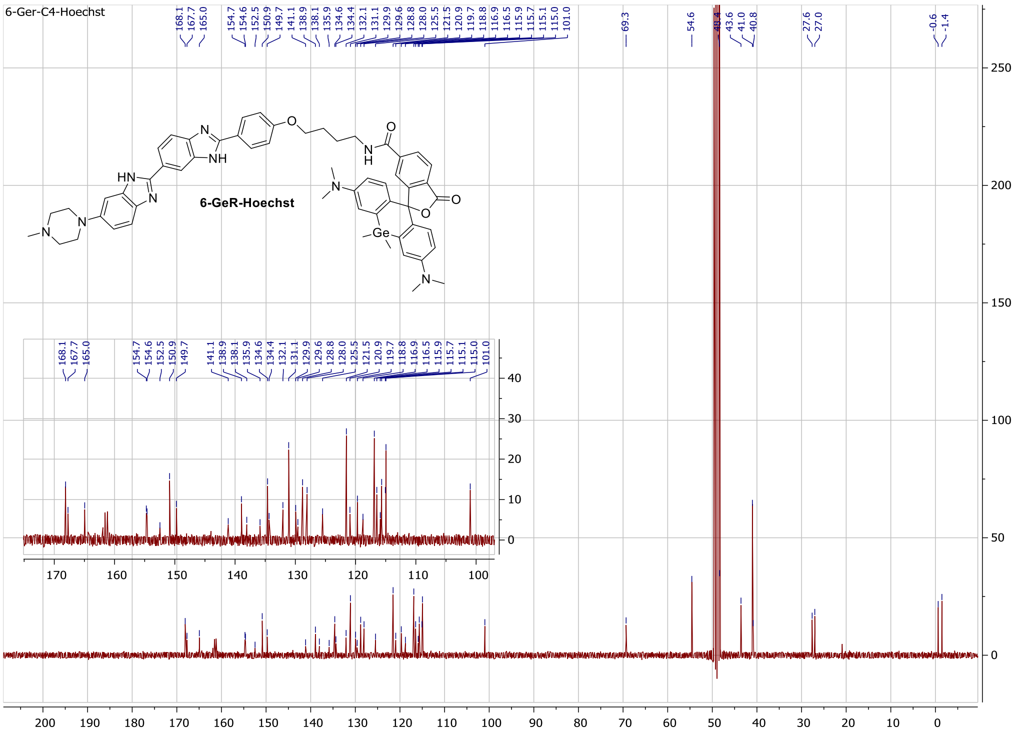

5-GeR-Hoechst

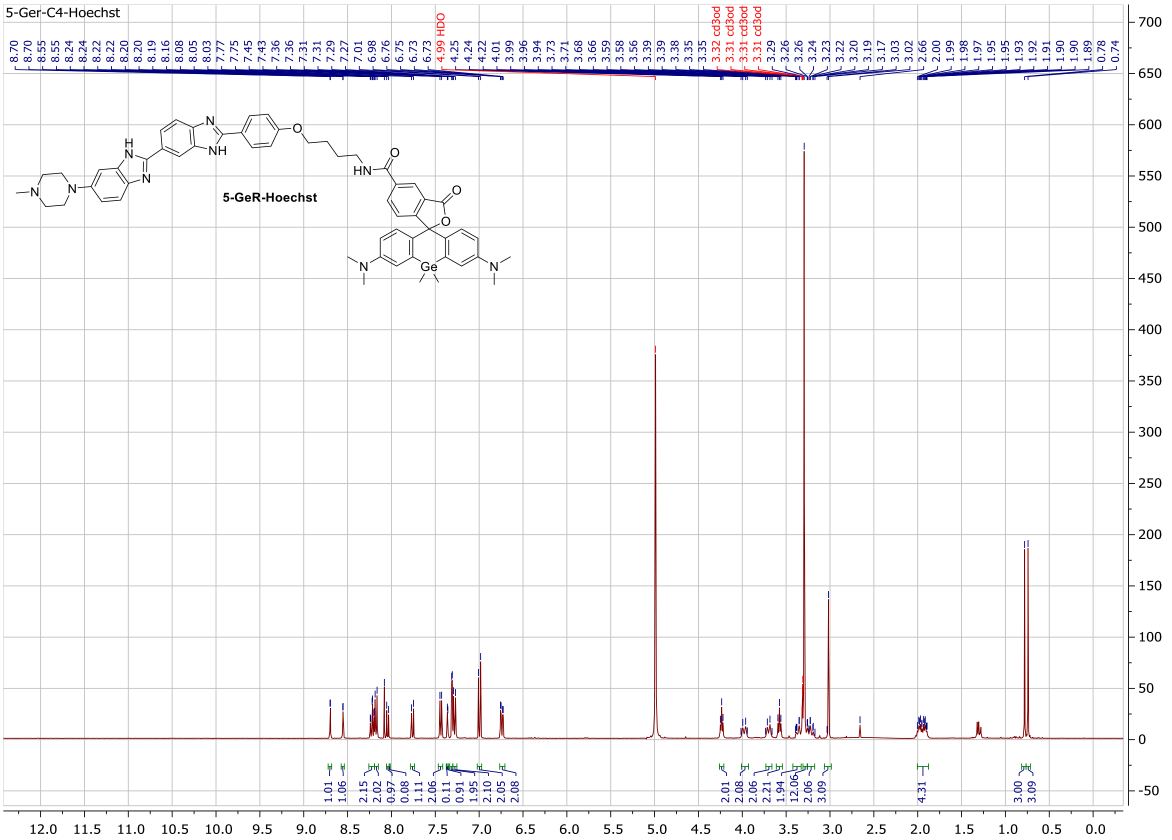

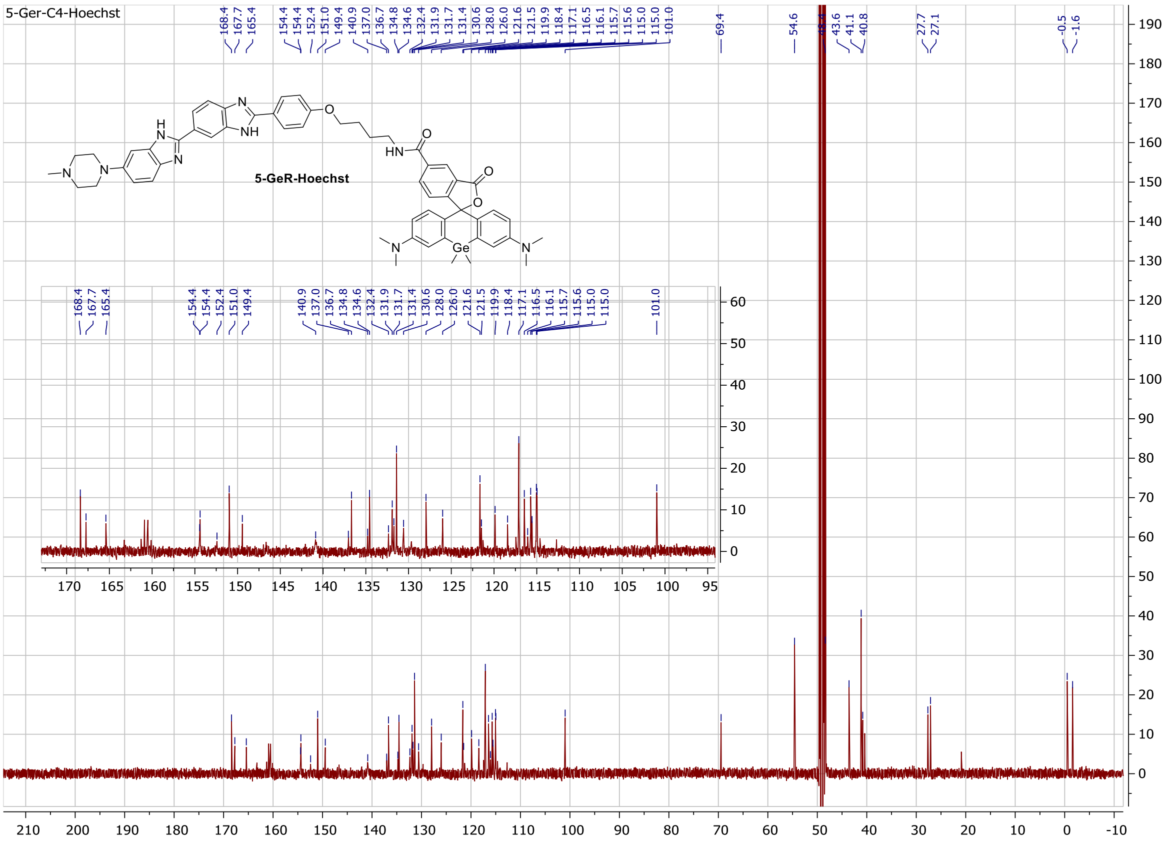

Supplement: Supplementary file 1 [file SC-010-C8SC05082A-s001.pdf]
